# A Column Chromatography-Free Protocol for the Synthesis of Water-Soluble Cavitands

**DOI:** 10.1021/acs.joc.5c00725

**Published:** 2025-07-02

**Authors:** Gabriele Zirpoli, Manuel Petroselli

**Affiliations:** † Department of Science and Technological Innovation, University of Eastern Piedmont, Via Michel 11, Alessandria 15121, Italy; ‡ Laboratorium für Organische Chemie, 27219 ETH Zürich, Vladimir-Prelog-Weg 3, Zürich 8093, Switzerland

## Abstract

An efficient synthetic protocol for the synthesis of a library of unprecedented resorcin[4]­arene-based water-soluble molecular containers is reported here. Key to this approach is the tetra-iodo derivative **3**, named “*Key Iodo Cavitand*” (KIC), which acts as a concave-shaped scaffold for the synthesis of deep-cavitands via Sonogashira coupling. Most of the deep-cavitands described in this study are synthesized without chromatographic processes, leading to a faster purification, ready accessibility, and easier scale-up, while also offering a different cavity in terms of size and shape compared to the current ones. Preliminary binding experiments on cyclodecane as a model guest are reported in D_2_O, while DFT calculations confirm the encapsulation of fullerene derivatives such as C_60_ and C_70_, highlighting the potential of the proposed molecular containers.

## Graphical abstract

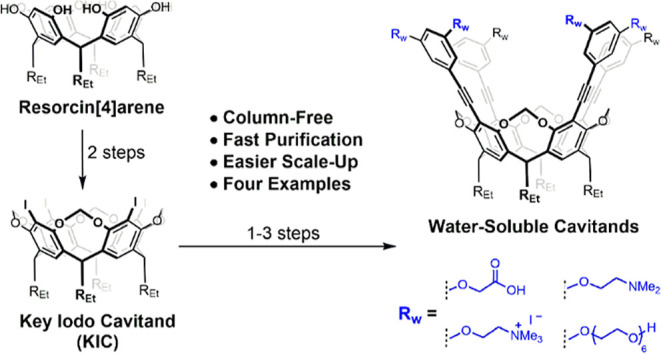

## Introduction

Enzymes use supramolecular interactions,^1^ such as hydrogen bonds,^2,3^ and the hydrophobic interactions,^4^ to encapsulate specific substrates in hydrophobic pockets called active sites, where reactions take place away from aqueous medium. Concave-shaped receptors ensure the formation of a cavity in which guests, exhibiting shape and size complementarity, can be encapsulated.^5,6^ Within this class of molecules, resorcin[4]­arene-based water-soluble deep-cavitands are key players, where “extended aromatic panels” create large lipophilic cavities that are structurally analogues to enzymatic pockets.^7–11^ In the past decade, they have been extensively studied as model receptors,^12^ contributing to the expansion of our understanding of the role of noncovalent interactions in chemical reactions within confined spaces^12–14^ and molecular recognition processes^15–18^ in aqueous solution. The synthesis of water-soluble deep-cavitands is often quite challenging due to (i) the low solubility of key intermediates, (ii) the chromatographic processes required to purify intermediates and final products, and (iii) the low-overall yield of the entire process (often <20%), which results from the mentioned issues.^19^

Few synthetic methodologies have been recently reported that provide easy access to cavitands with different binding properties and dynamic equilibria in solution. Rebek et al. reported the synthesis of the Octa-Methyl Urea Cavitand (OMC)^18^ (Scheme 1A) where each aromatic panel is independent and fully flexible, leading to the formation of a receptive structure, called vase form, stabilized by the presence of suitable guests.^20^ This high flexibility makes OMC extremely versatile toward a wide range of guests, allowing the study of molecular recognition processes^21^ and chemical reactions in its cavity, such as macrocyclizations^22^ and monofunctionalizations,^23–25^ even through radical mechanisms.^26,27^ Large guests, such as 1,3,5-trimethylbenzene (mesitylene) or fullerene derivatives, are not encapsulated in the OMC, highlighting its limitations. The water solubility is commonly ensured by the presence of water-solubilizing groups, such as imidazolium or pyridinium salts, on the lower rim,^28^ like in OMC, or through the functionalization of the upper rim.^29^ In contrast, Octa-Acid Cavitand (OA), reported by Gibb et al.,^30^ features covalently bound aromatic walls that force the host to adopt a more rigid concave-shaped structure compared to OMC (Scheme 1B). The lower flexibility restricts chemical-reactivity studies on OA to photoactivated processes^31,32^ and to guests suitable for encapsulation within capsules.^33^ However, its study in molecular recognition processes opens up a broad range of applications.^34–36^ The presence of water-solubilizing groups (R_w_) on the upper and lower rims of the host ensures high water solubility, as observed for OCM. It is worth mentioning that an updated protocol for the synthesis of OA has been recently reported, which avoids the use of chromatographic processes and improves the overall yield.^37^ The growing importance and impact of molecular receptors in medicinal chemistry^38^ prompted us to propose an alternative to current protocols.

**Scheme 1:**
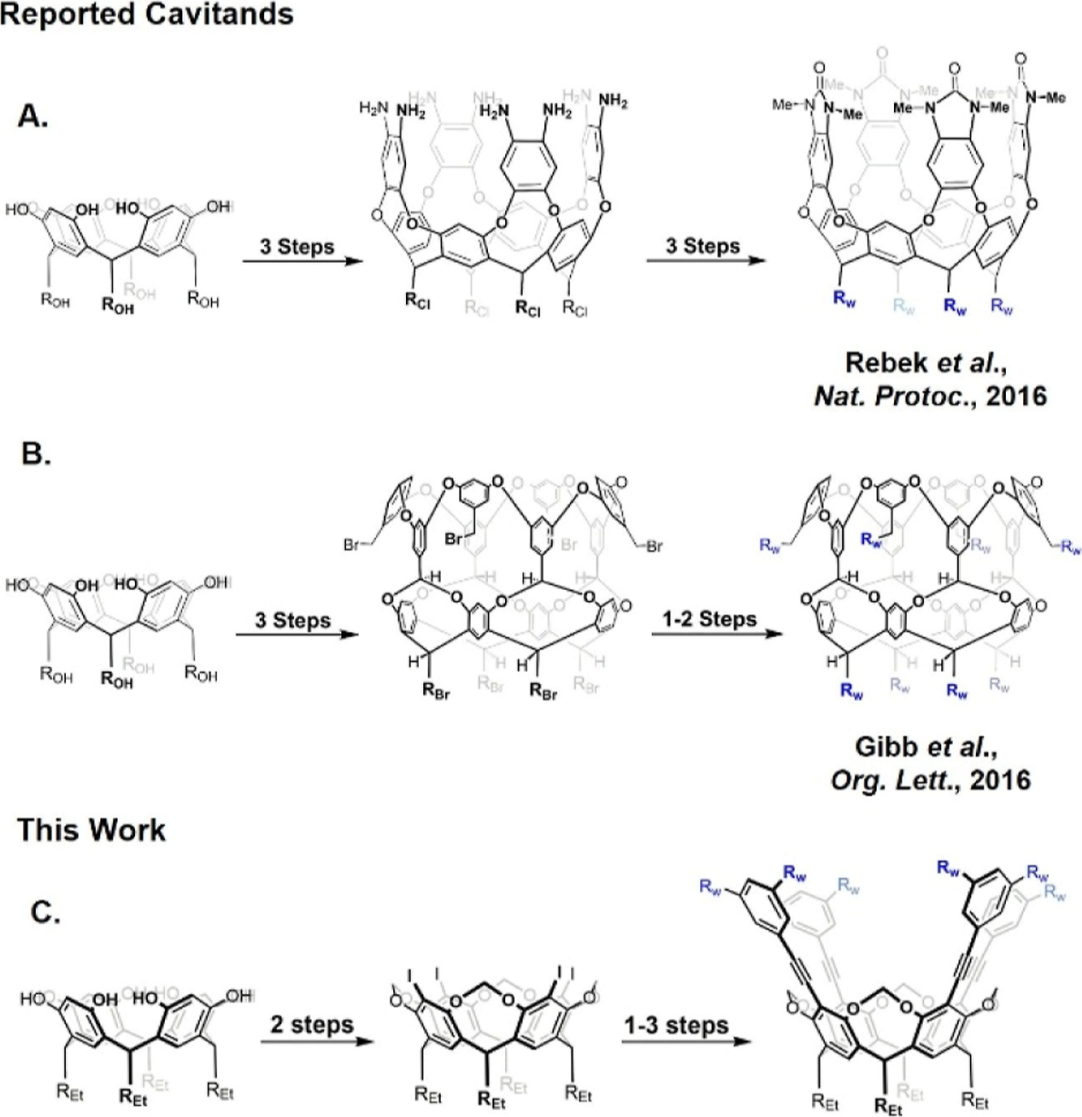
Schematic Methodology and Intermediates for the Synthesis of: (A) Octa-Methyl Urea Cavitand (OMC), (B) Octa-Acid Cavitand (OA), and (C) Hosts Proposed in This Study^a^

a R_w_ = water-solubilizing groups.

## Results and Discussion

We designed an efficient and versatile synthetic methodology (without chromatographic processes) for the synthesis of an unprecedented library of organic and water-soluble molecular containers, starting from the easily accessible resorcin[4]­arene **1**,^20^ with cationic, anionic, and neutral water-solubilizing groups, located in the upper rim of the host. The cavitands proposed in this study feature cavities with different sizes, shapes, and chemical environments compared to those of OMC and OA (Scheme 1C), thereby contributing to the expansion of the molecular receptor family capable of mimicking the action of biological enzymes (vide infra). Palladium-catalyzed cross-coupling reactions, such as the Sonogashira coupling^39^ between an activated resorcin[4]­arene halide (e.g., cavitand **2** or **3**) and an alkyne (e.g., alkyne **4**–**6**), are well-known protocols for forming deep-cavitands (Scheme S1).^40–42^ Taking inspiration from previous Cram results,^43^ we targeted the activated tetra-iodo resorcin[4]­arene **3**, namely, key iodo-cavitand (KIC) as a key intermediate to constitute the concave-shaped scaffold of the reported deep-cavitands. Cavitand **3** (KIC) was chosen for the following reasons: (i) higher reactivity toward alkyne derivatives under Sonogashira conditions compared to the corresponding bromide and tosylate derivatives, (ii) ease of functionalization, and (iii) formation of a preorganized and symmetric concave-shaped structure^44^ (Figure S67). The bowl-shaped conformation is also preserved in intermediates **1** and **2** due to the hydrogen bonding between the OH groups on the upper rim.^45–47^

The lipophilic version of the KIC precursor **2** has been reported and used as an intermediate for the development of organic-soluble cavitands.^29–31^ Diederich et al. reported the bromination of derivatives of **1** using *N*-bromosuccinimide (NBS) in butanone at room temperature for 24 h, affording the corresponding tetra-bromo cavitand in 52% yield.^42^ Treatment of the resulting cavitand^48^ with BuLi at −100 °C, followed by I_2_ quenching, affords the KIC derivative in 73% yield (Scheme S4). Purification on silica gel was required to obtain the pure cavitand.^42^ A single-step protocol, bypassing the formation of the tetra-bromo intermediate, was instead proposed by Dalcanale et al. where a derivative of **1** was treated with molecular I_2_ in the presence of NaHCO_3_ at room temperature for 12 h in a 1:1 mixture H_2_O/Et_2_O, yielding the derivative of **2** through precipitation in 36% yield (Scheme S3).^49^ The Dalcanale procedure introduced several advantages, such as a faster and safer protocol (single-step synthesis with no use of BuLi) and easier purification (no chromatographic processes), although a lower yield (≈27% less) was observed compared to the Diederich procedure. Inspired by the work of Diederich and Dalcanale, we aimed to develop a methodology for the synthesis of **2** by taking advantage of both synthetic strategies. Several conditions and iodine sources were tested to functionalize **1**, including the I_2_, I_2_/AgNO_3_, *N*-iodosuccinimide (NIS), and I_2_/urea hydrogen peroxide (UHP) complex. Most of these either led to no conversion or resulted in low yields with a mixture of iodinated byproducts. However, treatment of **1** with 2 equiv of molecular I_2_ in the presence of the UHP complex in acetonitrile (ACN) at 45 °C for 12 h afforded compound **2** in 78% yield (42 and 5% higher compared to the Dalcanale and Diederich procedures, respectively) (Scheme 2). Resorcin[4]­arene **1** shows a low solubility in ACN, and a simple filtration followed by washing with water yielded compound **2** in acceptable purity (>92%) (see the Supporting Information).^50^ Ring-closing reactions on derivatives of **2**, leading to the formation of bridged cavitands, are reported in the literature with several electrophiles, such as alkyl halides^51^ or benzylic halides as observed in the OA synthesis.^30^ The nature and length of the electrophile (e.g., CH_2_X_2_ vs (CH_2_)_2_X_2_ with X = Cl, Br or I) modulate the size and shape of the cavity, affecting the binding properties of the resulting host (Figure S68–S71). It is worth mentioning that the preorganization of **2**, due to the hydrogen bonding network on the upper rim (vide supra), provides proper orientation and good reactivity for the formation of bridged cavitands. For practical reasons, we focused on the methylene-bridged cavitands, although the same methodology has also been successfully applied to an ethylene-bridged variant (Figures S59 and S60). Diederich et al. reported a ring closing reaction on a derivative of **2** using TsO­(CH_2_)_*n*_OTs (with *n* = 1 or 2) as electrophiles in the presence of Cs_2_CO_3_ in DMSO at 65 °C for 40 h, yielding the KIC derivative in 34% yield after column chromatography.^42^ Similarly, Aakeröy et al. reported a ring-closing reaction on a derivative of **2** using bromochloromethane (CH_2_BrCl) and K_2_CO_3_ in DMF, affording the KIC derivative in 49% yield after 4 days and column chromatography.^40^

**Scheme 2:**
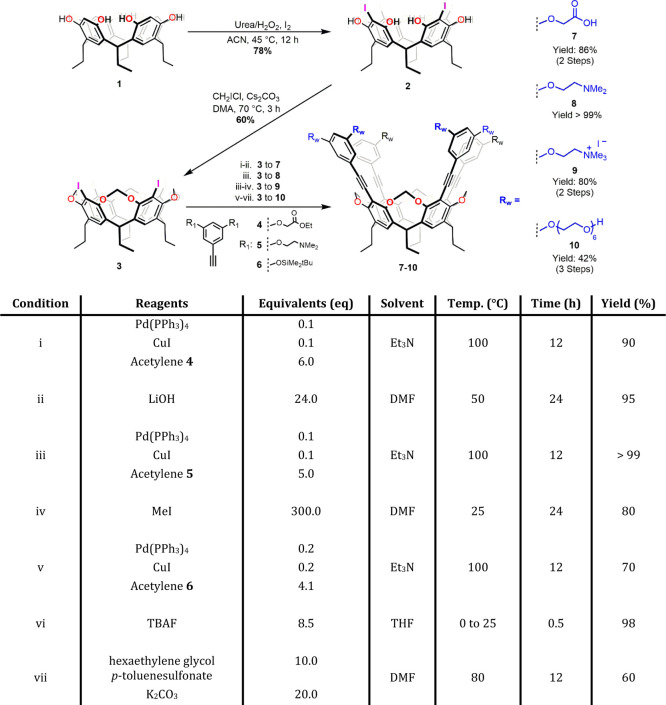
Synthesis of Water-Soluble Cavitands **7**–**10** from resorcin[4]­arene **1** through KIC Intermediate **3**

The instability of **2** (and its derivatives) in solvents like DMSO, DMA, and DMF requires careful selection of experimental conditions to ensure that ring-closing kinetics prevails over decomposition kinetics. The decomposition of **2** in DMSO, DMA, and DMF is likely due to two main factors: (i) the stabilizing HBs between the OH group(s) and iodine atom are weakened in the presence of polar solvents like DMSO and DMF, and (ii) the formation of halogen bonds between the iodine atom and the carbonyl group of the solvent molecules, which weakens the C–I bond. After several attempts, we found that the treatment of **2** with 10 equiv of chloroiodomethane (CH_2_ICl) and 30 equiv of Cs_2_CO_3_ at 70 °C for 3 h in DMA afforded the KIC (**3**) in 60% yield (Scheme 2). The higher yield (26% and 11% compared to the Diederich and Aekeröy procedures, respectively) should be mainly attributed to the use of CH_2_ICl, a more reactive electrophile that enables faster ring-closing reactions,^52^ reducing the reaction time (from >40 to 3 h) and limiting the formation of side products from the decomposition of **2**. Finally, a simple filtration of the reaction crude on a SiO_2_/Celite pad, followed by slow cooling crystallization from a refluxing ACN/dioxane solution, allows a column-free purification of **3** (Figures S55 and S56). A similar protocol was recently reported by Dalcanale et al.^53^ for ring-closing reactions on the methylated version of **2**, achieving an yield of 77%.^54^ Cavitand **3** (KIC) was fully characterized, and its structure was confirmed by single-crystal X-ray crystallography (see Figure 1A).^43^ The crystals contained four KIC molecules in the unit cell, with two molecules of ACN hosted, one inside the cavity and one in the proximity of the “feet” (Figure S57). The gram-scale accessibility of **3** (KIC) and the reactivity of the “iodine panels” toward alkynes such as **4**–**6** ensure a versatile and smooth functionalization under Sonogashira conditions. These alkyne derivatives were wisely selected to introduce water-solubilizing groups that are effective over a wide range of pH values (vide infra). The reaction conditions for the synthesis of alkynes **4**–**6** have been optimized to achieve individual reaction yields of no less than 60% (Schemes S5–S7). The reaction between KIC (**3**) and alkyne **4** (Scheme 2–Condition (i)) afforded the ester precursor of **7** in 90% yield, which was purified by crystallization through slow cooling of an ACN/EtOH solution. Its structure was fully characterized (see Supporting Information) and confirmed by single-crystal X-ray crystallography (Figures 1B and S55). Hydrolysis of the ester groups with LiOH in DMF gave cavitand **7** in 95% yield (Scheme 2 Condition (ii)). Purification of **7** was performed through subsequent extractions, avoiding any chromatographic process and taking advantage of the different solubility of the involved systems under acidic and basic conditions (Figure S17).^55^ Similarly, reaction between **3** (KIC) and alkyne **5** led to cavitand **8** in nearly quantitative yields (Scheme 2–Condition (iii)). As mentioned for **7**, purification of the resulting product required only subsequent extractions (Figure S26).^56^ Methylation of **8** with MeI in DMF at room temperature yielded positively charged cavitand **9** in 80% yield (Scheme 2–Condition (iv)). The latter could not undergo subsequent extractions; however, a simple wash with hexane^57^ gave pure **9** due to its low solubility in apolar solvent (Figure S29). A lower yield (≈70%) was instead observed for the reaction between KIC (**3**) and alkyne **6** (Scheme 2 (v)), probably due to the partial cleavage of the TBS-protecting groups under harsh conditions (100 °C). However, treatment of the resulting reaction crude with TBAF in THF at 0–25 °C for 30 min, followed by functionalization of the free phenolic groups with hexaethylene glycol chains, afforded cavitand **10** with an overall yield of 41% (Scheme 2–Conditions vi and vii). The phenolic intermediate could undergo the same extraction purification process as **7** and **8**, taking advantage of the formation of sodium phenolates.^58^ However, column chromatography on reverse silica gel using H_2_O/MeOH solutions was necessary to remove traces of unreacted PEG chains from the crude product and obtain pure **10** (Figure S36). Cavitand **9** and **10** showed good solubility in D_2_O at neutral pH, although lower water solubility is reported for similar derivatives.^59^ Instead, cavitands **7** and **8** required basic and acid pH conditions to form carboxylate and ammonium salts, respectively, and dissolve in solution.^30^ The chemical diversity of the side chains and the different self-aggregation kinetics with respect to the NMR time scale lead for some hosts to a relatively low resolution of the NMR spectra in a D_2_O solution. This is extreme for **10** where broader peaks were recorded in D_2_O. However, sharper peaks were always detected after inclusion of suitable guests (e.g., cyclodecane)^60^ (Figures S63–S66). All hosts exhibited excellent binding properties, confirmed by the inclusion of cyclodecane, which was taken into account as a model guest for our purposes (see the Supporting Information). Moreover, the larger cavity compared to OMC and OA (Figures S68–S70) is expected to allow the inclusion of larger guests, such as fullerene derivatives, which have recently became interesting targets in host–guest chemistry.^61–64^ This hypothesis was preliminarily confirmed through a computational study on fullerene@host complexes (Figure S72).

**Figure 1:**
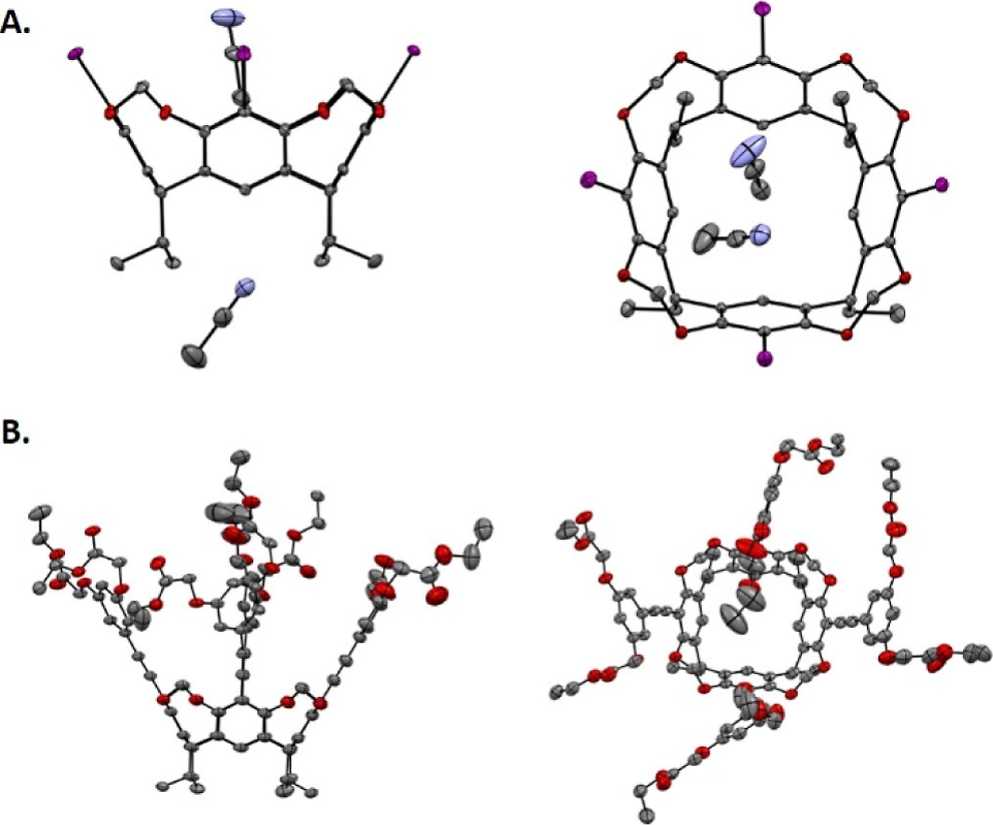
Side (left) and top (right) views of the X-ray crystal structure (the ellipsoid contour at 50% probability levels) of (A) compound **3** (KIC–Deposition Number CDCC 2430285) and (B) ester derivative of 7 (Deposition Number CDCC 2430286).

## Conclusion

We have developed a column chromatography-free methodology for large-scale access to the KIC, which acts as a versatile concave-shaped scaffold for the construction of a library of organic and water-soluble molecular containers via the Sonogashira coupling reaction. The new hosts were obtained, in most cases, in good yield and without the need for chromatographic processes, demonstrating good binding properties and a larger cavity compared to the current deep-cavitands reported in the literature. Studies on the effect of Coulombic interactions^65^ on the binding properties are currently ongoing, aiming to extend our understanding on the role of noncovalent interactions in molecular recognition processes.

## Supplementary Material



## Data Availability

The data underlying this study are available in the published article and its Supporting Information.

## Notes

Experimental procedures, characterization details, ^1^H- and ^13^C NMR spectra, HRMS spectra, binding experiments in D_2_O, computational calculations, and X-ray crystallographic data of new compounds (PDF)
