# Supplementary material for: A Column Chromatography-Free Protocol for the Synthesis of Water-Soluble Cavitands
Source: J Org Chem. 2025 Jul 2;90(28):9770–5. doi: 10.1021/acs.joc.5c00725 (PMC12281564; doi:10.1021/acs.joc.5c00725)
Supplement: Supplementary file 1 [file jo5c00725_si_001.pdf]

Supporting Information

A Column Chromatography-Free Protocol for the Synthesis of Water-Soluble Cavitands

Gabriele Zirpoli[b] and Manuel Petroselli*[a,b]

[a]Department of Science and Technological Innovation, University of Eastern Piedmont, Via Michel 11, 15121 Alessandria, Italy.

[b]Laboratorium für Organische Chemie, ETH Zürich, Vladimir-Prelog-Weg 3, Zürich 8093, Switzerland.

Email: manuel.petroselli@uniupo.it

Table of Contents

1. General Information, Material & Methods S4
   1.1 Reagents and Solvents S4
   1.2 Thin Layer Chromatography (TLC) S4
   1.3 Flash Chromatography S4
   1.4 ^{1}H NMR Spectroscopy S4
   1.5 ^{13}C NMR Spectroscopy S4
   1.6 Infrared (IR) Spectroscopy S4
   1.7 High Resolution Mass Spectroscopy (HR-MS) S5
   1.8 Melting Point Analysis S5
2. Experimental Procedures S6
3. Synthetic Schemes S18
4. Spectral Data S24
   4.1 NMR Spectra S24
   4.2 MS Spectra S46
5. Crystallographic Data S54
6. Binding Experiments S60
7. Computational Studies S63
8. References S74

1. General Information, Material & Methods

1.1 Reagents and Solvents

Reagents and solvents (ABCR, Acros, Sigma Aldrich, Fluka, TCI) were used without prior purification. Reactions were performed in analytical grade solvents under a N_2 atmosphere and under exclusion of water or air in standard glassware unless stated otherwise. Air and moisture sensitive reactions were carried out in extra dry solvents over 5A molecular sieves stored under a N_2 atmosphere (Acros and Sigma Aldrich). Technical grade solvents were utilized for workups and washes. Concentration under reduced pressure was performed by rotary evaporation at 40 °C. Purified compounds were further dried under high vacuum.

1.2 Thin Layer Chromatography (TLC)

TLC was performed on *Merck silica gel 60 F254 TLC glass plates* and visualized with 254 nm light and potassium permanganate or *p*-anisaldehyde staining solutions followed by heating.

1.3 Flash Chromatography

Flash chromatography was carried out using the *Büchi Reveleris X2 Flash Chromatography System* with 20-63 µm silica cartridges and technical grade solvents. The flow rates varied up to 200 mL/min depending on the column and moderate pressures of less than 100 psig were maintained. Manual flash chromatographic purification (if necessary) was performed on SiO_2 32-63, 60 Å under 0.3-0.5 bar overpressure.

1.4 ^{1}H NMR Spectroscopy

^{1}H-NMR spectra were recorded on a *Bruker AVIII 600 MHz spectrometer* with He or prodigy N_2 cryo-probes, *Bruker VIII HD 500 MHz* and *400 MHz spectrometers* as well as *Bruker Neo 500 MHz* and *400 MHz spectrometers* and are reported in ppm with the solvent resonance as reference ($CDCl_3$ at 7.26 ppm, DMSO-d_6 at 2.50 ppm, D_2O at 4.79 ppm). Peaks are reported as s = singlet, d = doublet, t = triplet, q = quartet, p = pentet, m = multiplet, br = broad signals. Coupling constant(s) J are given in Hz. Integration value for the diastereotopic protons (4H in a single cavitand) of the methylene-bridge (O-CH_2-O) has been set at 4.00 and taken as internal reference. Structural assignments were made with additional information from gCOSY experiments.

1.5 ^{13}C NMR Spectroscopy

^{13}C-NMR spectra were recorded with ^{1}H-decoupling on Bruker AVIII 150 MHz spectrometers with He or prodigy N_2 cryo-probes, *Bruker AVIII HD 125 MHz,* and *100 MHz spectrometers* as well as *Bruker Neo 125 MHz* and *100 MHz spectrometers*. Resonances are reported in ppm with the solvent resonance as reference ($CDCl_3$ at 77.16 ppm, DMSO-d_6 at 39.52 ppm).

1.6 Infrared (IR) Spectroscopy

IR spectra were recorded neat on a *Perkin-Elmer Spectrum Two FT-IR* spectrometer. The peaks are reported as absorption maxima (cm^{-1}).

1.7 High Resolution Mass Spectroscopy (HR-MS)

HR-MS measurements were performed by the MS service at the Laboratory of Organic Chemistry of ETH Zurich on a *Bruker Daltonics maXis ESI-QTOF* for EI-MS, on a *Bruker maXis* spectrometer for ESI-MS, and on a *Bruker Daltonics solariX* for MALDI/ESI; Masses are reported in *m/z* units as the molecule ion $[M]^+$, $[M+H]^+$, $[M+Na]^+$, $[M+K]^+$ or $[M+NH_4]^+$.

1.8 X-Ray Analysis

Single crystalline samples were measured on a Bruker Kappa APEX-II Duo diffractometer with sealed tube Mo-Kα radiation (λ = 0.71073 Å) or a Bruker/Nonius Kappa APEX-II diffractometer with microfocus sealed tube Mo-Kα radiation using mirror optics (λ = 0.71073 Å).

1.9 Melting Point Analysis

Melting points were determined on a *Büchi B545 Melting Point Apparatus*.

2. Experimental Procedures

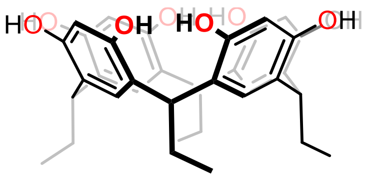

C-ethylcalix[4]resorcinarene (1)

Following our modification of a literature procedure, [1] to a solution of resorcinol (20 g, 181.6 mmol) in EtOH : H_2O (1:1 v/v, 90 mL) is added HCl (37%, 22.5 mL) dropwise. A solution of propionaldehyde (13.1 mL, 10.5 mmol, 1 equiv) in EtOH (90 mL) is then added and the reaction mixture is stirred at 50 °C for 3 days in an oil bath. Precipitation of a beige solid occurs within the first day. After cooling to room temperature, the solid is filtered, washed with a cold solution of EtOH : H_2O (1:1 v/v) and dried at 80 °C under rotary evaporation for at least 1 h and eventually under high vacuum to yield pure compound **1** (9.3 g, 15.4 mmol, 34%) as a beige solid.

^{1}H-NMR (500 MHz, DMSO-d$_6$, 298 K): δ 8.92 (s, 8H), 7.23 (s, 4H), 6.14 (s, 4H), 4.09 (t, J = 7.9 Hz, 4H), 2.11 (p, J = 7.3 Hz, 8H), 0.79 (t, J = 7.2 Hz, 12H); **^{13}C-NMR {^{1}H}** (126 MHz, DMSO-d$_6$, 298 K) δ 151.7, 125.0, 123.1, 102.3, 35.2, 26.7, 12.7; **FT-IR** (neat): 3528, 3476, 3322, 2959, 2929, 2871, 1614, 1504, 1435, 1377, 1331, 1295, 1280, 1193, 1149, 1109, 1099, 1059, 1030, 932, 892, 844, 826, 774, 759, 597, 532, 518, 491, 455 cm^{-1}; **HRMS** (MALDI/ESI) m/z: [M]$^+$ Calcd for $C_{36}H_{40}O_8$ 600.2718; Found 600.2717. **TLC**: R_f = 0.28 (10:1 DCM/MeOH, p-anisaldehyde); **mp**: > 410 °C (slowly decomposing above 300 °C).

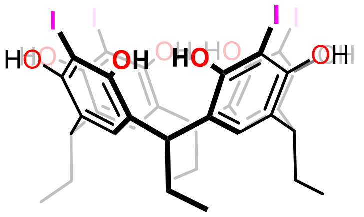

C-ethyl-tetraiodocalix[4]resorcinarene (2)

To a suspension of **1** (2 g, 3.3 mmol) in ACN (65 mL) are added Urea/H_2O_2 (657 mg, 6.9 mmol, 2.1 equiv) and I_2 (1.7 g, 6.9 mmol, 2.1 equiv). The mixture is stirred in the dark overnight at 45 °C in an oil bath. The next morning, almost complete discoloration occurred (a slight excess of I_2 was used) and a light beige solid is observed. The latter is filtered, rinsed copiously with distilled water and dried at 80 °C for 2 h under reduced pressure to yield pure **2** (2.9 g, 2.6 mmol, 78%) as a white solid.

^{1}H-NMR (500 MHz, DMSO-d$_6$, 298 K): δ 9.30 (s, 8H), 7.53 (s, 4H), 4.18 (t, 4H), 2.32 (p, 8H), 0.83 (t, 12H); **^{13}C-NMR {^{1}H}** (126 MHz, DMSO-d$_6$, 298 K): δ 150.9, 125.9, 124.7, 81.3, 38.2, 26.1, 12.4; **FT-IR** (neat): 3399, 2962, 2871, 1606, 1463, 1438, 1380, 1299, 1255, 1197, 1147, 1100,

1062, 1035, 945, 902, 817, 767, 675, 638, 564 cm^{-1}; **HRMS** (MALDI/ESI) m/z: $[M]^+$ Calcd for $C_{36}H_{36}I_4O_8$ 1103.8584; Found 1103.8582. **mp:** decomposition above 240 °C.

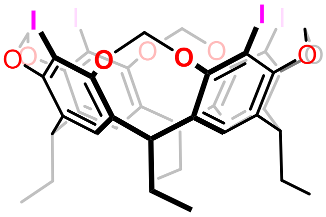

Key Iodo Cavitand **(KIC)** (**3**)

Compound **2** (2.50 g, 2.26 mmol) is loaded into a three-neck flask and, in the absence of light, dissolved in DMA extra dry (90.6 mL, 25 mM) under a N_2 atmosphere and with the help of a mechanical stirrer. The latter is necessary to maintain a fine dispersity of the solid suspension throughout the reaction. After warming the solution to 70 °C in an oil bath, Cs_2CO_3 (22.1 g, 67.9 mmol, 30 equiv) and, 10 minutes later, $ClCH_2I$ (3.31 mL, 45.3 mmol, 20 equiv) are added. The mixture is then stirred in the dark for 3 h at 70 °C under strong mechanical stirring. Following the removal of the solvent at 80 °C under reduced pressure, the obtained solid is picked up in distilled water and sonicated for 30 minutes. The resulting fine beige powder is filtered, washed with water and methanol, and finally dissolved in $CHCl_3$ and picked up through the filter to leave traces of insoluble impurities. The crude solid is a mixture of the desired product **3** and ca 15% of its tris-Iodo derivative arising from the slow decomposition of the starting material during the reaction. A simple filtration of the reaction crude on a pad of SiO2/celite followed by slow cooling crystallization from a refluxing ACN/dioxane solution afford pure **3** as a white solid (1.56 g, 60%).

^{1}H-NMR (500 MHz, $CDCl_3$, 298 K): δ 7.07 (s, 4H), 5.98 (d, J = 7.4 Hz, 4H), 5.30 (s, 2H), 4.77 (t, J = 8.1 Hz, 4H), 4.33 (d, J = 7.4 Hz, 4H), 2.30 – 2.20 (m, 8H), 1.00 (t, J = 7.2 Hz, 12H); **^{13}C-NMR {^{1}H}** (126 MHz, $CDCl_3$, 298 K): δ 155.1, 138.7, 120.7, 98.9, 93.2, 40.0, 23.4, 12.3.; **FT-IR** (neat): 2961, 2933, 2873, 1463, 1443, 1410, 1382, 1323, 1297, 1224, 1178, 1140, 1091, 1067, 1043, 1017, 993, 966, 926, 896, 823, 786, 768, 733, 651, 639, 611, 587, 574, 532 cm^{-1}; **HRMS** (ESI) m/z: $[M+NH_4]^+$ Calcd for $C_{40}H_{40}I_4NO_8$ 1169.8927; Found 1169.8921. **TLC**: R_f = 0.28 (1:1 Hexane/DCM, p-anisaldehyde); **mp**: decomposition above 385 °C.

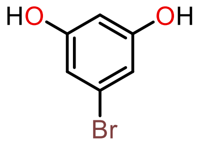

5-bromobenzene-1,3-diol (A)

Following our modification of a reported procedure, to an ice-cold solution of 1-bromo-3,5-dimethoxybenzene (5 g, 23 mmol) in DCM extra dry (100 mL) is added BBr_3 (7.64 mL, 80.6 mmol, 3.5 equiv) dropwise. The mixture is then warmed to room temperature and stirred for 24 h. After cooling the reaction mixture to 0 °C, a saturated aq. $NaHCO_3$ (*ca.* 70 mL) solution

is slowly added until no more gas evolution is observed. A 1 M NaOH solution is then carefully added until the mixture reaches a pH of *ca.* 11. The DCM phase is then separated from the aqueous phase and discarded. The latter is extracted once with DCM, acidified to a pH of *ca.* 2 and then extracted twice with EtOAc. The collected organic layers are dried under $MgSO_4$ and filtered. Concentration *in vacuo* affords a brown oil. Flash column chromatography (SiO_2; Hexane-EtOAc gradient on Büchi Reveleris X2) yields the title compound as a white solid (3.6 g, 19.0 mmol, 83%).

^{1}H-NMR (500 MHz, DMSO-d_6, 298 K): δ 9.66 (s, 2H), 6.37 (d, J = 2.1 Hz, 2H), 6.18 (t, J = 2.1 Hz, 1H); **^{13}C-NMR {^{1}H}** (126 MHz, DMSO-d_6, 298 K): δ 159.4, 121.8, 109.4, 101.8; **FT-IR** (neat): 3259, 1595, 1469, 1347, 1288, 1196, 1151, 990, 822, 665, 594, 557, 534, 519 cm^{-1}; **HRMS** (EI) m/z: $[M]^+$ Calcd for $C_6H_5O_2Br$ 187.9467; Found 187.9464. **TLC**: R_f = 0.41 (2:1 Hexane/Ethyl Acetate, p-anisaldehyde); **mp**: 95 °C.

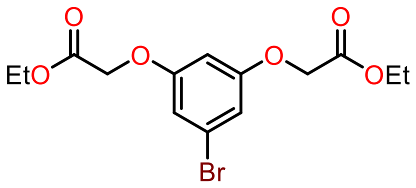

Diethyl-2,2'-((5-bromo-1,3-phenylene)bis(oxy))diacetate (B)

To a suspension of K_2CO_3 (2.85 g, 20.6 mmol, 3.9 equiv) in a solution of **A** (1 g, 5.3 mmol) in extra dry acetone (20 mL) is added ethyl bromoacetate (2.3 mL, 20.6 mmol, 3.9 equiv). The mixture is stirred at room temperature for 3 h. The solid is filtered off and the filtrate is concentrated *in vacuo*. The resulting oil is picked up in DCM and extracted with a saturated aq. NH_4Cl solution. The collected organic phase is dried under $MgSO_4$ and filtered. Concentration *in vacuo* afforded the title compound as a white solid (1.9 g, 5.26 mmol, quantitative).

^{1}H-NMR (500 MHz, $CDCl_3$, 298 K): δ 6.69 (d, J = 2.3 Hz, 2H), 6.43 (t, J = 2.3 Hz, 1H), 4.56 (s, 4H), 4.27 (q, J = 7.1 Hz, 4H), 1.30 (t, J = 7.2 Hz, 6H); **^{13}C-NMR {^{1}H}** (126 MHz, $CDCl_3$, 298 K): δ 168.3, 159.5, 123.1, 111.6, 101.4, 65.6, 61.7, 14.3; **FT-IR** (thin film): 2996, 2974, 2933, 1747, 1596, 1580, 1441, 1395, 1379, 1333, 1290, 1276, 1256, 1199, 1159, 1108, 1081, 1028, 990, 944, 864, 844, 827, 808, 729, 705, 674, 628, 613, 583, 544, 524; **HRMS** (ESI) m/z: $[M]^+$ Calcd for $C_{14}H_{18}BrO_6$ 361.0281; Found 361.0276. **TLC**: R_f = 0.19 (pure DCM, p-anisaldehyde); **mp**: 89 °C.

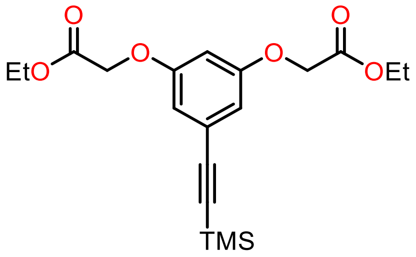

Diethyl-2,2'-((5-((trimethylsilyl)ethynyl)-1,3-phenylene)bis(oxy))diacetate (C)

To a solution of **B** (1.84 g, 5.09 mmol) in freshly distilled Et_3N (51 mL) is added [Pd(PPh_3)$_4$] (294 mg, 0.255 mmol, 0.05 equiv), CuI (48.5 mg, 0.255 mmol, 0.05 equiv). and Trimethylsilyl-acetylene (1.5 mL, 10.2 mmol, 2 equiv). After subjecting the mixture to three cycles of freeze-pump-thaw degassing, the latter is stirred at 70 °C for 12 h in an oil bath. The solvent is concentrated *in vacuo* and the remaining liquid is diluted with DCM and extracted once with a saturated aq. NH_4Cl solution. The organic phase is dried under $MgSO_4$, filtered and concentrated *in vacuo*. Flash column chromatography (SiO_2; pure DCM on Büchi Reveleris X2) yields the title compound as a yellow oil (1.76 g, 4.65 mmol, 91%).

^{1}H-NMR (500 MHz, $CDCl_3$, 298 K): δ 6.63 (d, J = 2.3 Hz, 2H), 6.51 (t, J = 2.3 Hz, 1H), 4.57 (s, 4H), 4.27 (q, J = 7.1 Hz, 4H), 1.30 (t, J = 7.2 Hz, 6H), 0.24 (s, 9H); **^{13}C-NMR {^{1}H}** (126 MHz, $CDCl_3$, 298 K): δ 168.6, 158.8, 124.9, 111.4, 104.5, 103.8, 94.7, 65.6, 61.6, 14.3, 0.03; **FT-IR** (thin film): 2961, 2903, 2160, 1757, 1737, 1588, 1465, 1431, 1379, 1333, 1300, 1277, 1249, 1208, 1156, 1085, 1029, 988, 970, 949, 843, 760, 700, 678, 645, 617, 582, 520 cm^{-1}; **HRMS** (ESI) m/z: $[M+H]^+$ Calcd for $C_{19}H_{27}O_6Si$ 379.1571; Found 379.1569. **TLC**: R_f = 0.5 (pure DCM, p-anisaldehyde).

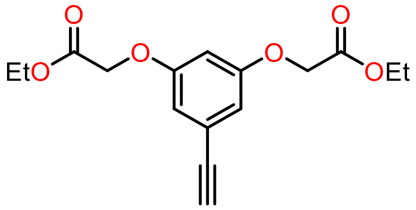

Diethyl-2,2'-((5-ethynyl-1,3-phenylene)bis(oxy))diacetate (4)

To an ice-cold solution of **C** (1.88 g, 4.97 mmol) in THF (10 mL, 0.5 M) and under a N_2 atmosphere is added dropwise a 1M TBAF in THF solution (5.46 mL, 5.46 mmol, 1.1 equiv). The mixture is warmed to room temperature and stirred for 30 minutes and then quenched with a few drops of a saturated aq. NH_4Cl solution. The organic phase is extracted with brine, dried under $MgSO_4$, filtered, and briefly concentrated *in vacuo*. Filtration on a pad of SiO_2/celite in pure DCM yields the title compound as a colourless oil (1.03 g, 3.36 mmol, 68%) after removal of the solvent *in vacuo*.

^{1}H-NMR (500 MHz, $CDCl_3$, 298 K): δ 6.66 (d, J = 2.4 Hz, 2H), 6.53 (t, J = 2.4 Hz, 1H), 4.58 (s, 4H), 4.27 (q, J = 7.1 Hz, 4H), 1.30 (t, J = 7.1 Hz, 6H); **^{13}C-NMR {^{1}H}** (126 MHz, $CDCl_3$, 298 K): δ 168.4, 158.7, 123.8, 111.5, 103.7, 83.0, 77.4, 65.4, 61.5, 14.2; **FT-IR** (thin film): 3278, 2982, 2936,

1752, 1589, 1431, 1378, 1326, 1277, 1210, 1162, 1086, 1028, 944, 854, 678 cm^{-1}; **HRMS** (ESI) m/z: $[M+H]^+$ Calcd for $C_{16}H_{19}O_6$ 307.1176; Found 307.1175. **TLC**: R_f = 0.19 (pure DCM, p-anisaldehyde).

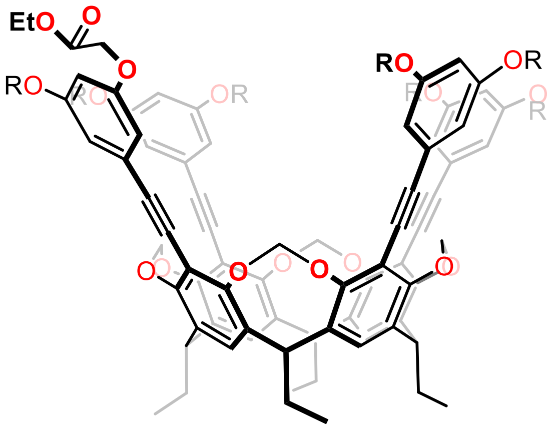

Octa-Ester Cavitand (D)

To a solution of **3** (618 mg, 536 µmol) in freshly distilled Et_3N (22 mL, 25 mM), kept over 5A MS, is added $[Pd(PPh_3)_4]$ (62 mg, 53.6 µmol, 0.1 equiv), CuI (10.2 mg, 53.6 µmol, 0.1 equiv) and **4** (986 mL, 3.2 mmol, 6 equiv). After subjecting the mixture to three cycles of freeze-pump-thaw degassing, the latter is stirred at 100 °C for 12 h in an oil bath. The solvent is concentrated *in vacuo* and the remaining liquid is diluted with DCM and extracted once with a saturated aq. NH_4Cl solution. The organic phase is dried under $MgSO_4$, filtered, and briefly concentrated *in vacuo*. Filtration on a pad of SiO_2/celite in 1:1 Hexane/EtOAc and subsequent slow-cooling crystallization from a refluxing ACN/EtOH solution yields the title compound as a white solid (900 mg, 0.48 mmol, 90%).

^{1}H-NMR (500 MHz, $CDCl_3$, 298 K): δ 7.10 (s, 4H), 6.65 (d, J = 2.3 Hz, 8H), 6.47 (t, J = 2.3 Hz, 4H), 6.00 (d, J = 7.3 Hz, 4H), 4.77 (t, J = 8.1 Hz, 4H), 4.60 (d, J = 7.2 Hz, 4H), 4.57 (s, 16H), 4.22 (q, J = 7.1 Hz, 16H), 2.28 (p, J = 7.4 Hz, 8H), 1.25 (t, J = 7.1 Hz, 24H), 1.03 (t, J = 7.2 Hz, 12H); **^{13}C-NMR {^{1}H}** (126 MHz, $CDCl_3$, 298 K): δ 168.4, 158.7, 155.5, 138.3, 124.7, 120.2, 113.0, 111.3, 103.1, 98.6, 97.1, 81.2, 65.5, 61.5, 38.5, 22.8, 14.1, 12.3; **FT-IR** (thin film): 2964, 1755, 1733, 1587, 1444, 1396, 1379, 1297, 1208, 1159, 1089, 1017, 994, 964, 929, 846, 828, 740, 677, 583, 530; **HRMS** (MALDI/ESI) m/z: $[M+Na]^+$ Calcd for $C_{104}H_{104}NaO_{32}$ 1887.6403; Found 1887.6413. **TLC**: R_f = 0.37 (1:1 Hexane/EtOAc, p-anisaldehyde); **mp:** decomposition above 180 °C.

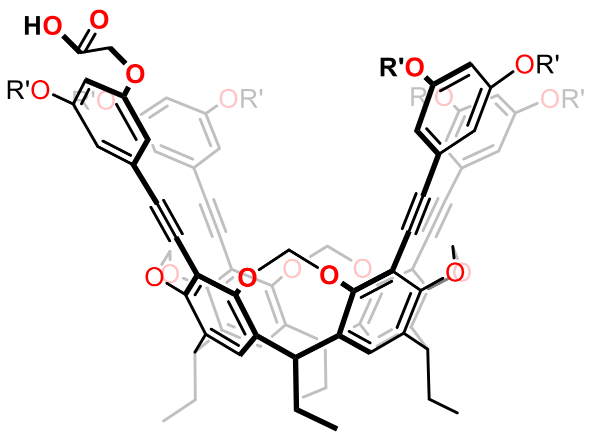

Cavitand 7

To a solution of **D** (84 mg, 0.045 mmol) in DMA (9 mL) is added an aq. LiOH (2 M, 0.54 mL) solution. The mixture is stirred for 48 h at 50 °C in an oil bath. Water is then added followed by enough aq. 0.1 M NaOH solution to raise the pH to 11. The aqueous phase is then washed twice with EtOAc, acidified with a 0.1 M HCl solution to pH 2 and then extracted twice with EtOAc. The organic phase is then washed twice with distilled water, dried under $MgSO_4$, filtered, and organic solvent removed under reduced pressure to yield cavitand **7** as a white solid (70.5 mg, 0.043 mmol, 95%).

^{1}H-NMR (500 MHz, DMSO-d6, 298 K): δ 13.05 (s, 8H), 7.65 (s, 4H), 6.63 (d, J = 2.3 Hz, 8H), 6.52 (t, J = 2.3 Hz, 4H), 6.16 (d, J = 7.5 Hz, 4H), 4.68 (s, 16H), 4.59 (t, J = 8.1 Hz, 4H), 4.48 (d, J = 7.5 Hz, 4H), 2.48 – 2.42 (m, 8H), 0.98 (t, J = 7.1 Hz, 12H); **^{13}C-NMR {^{1}H}** (126 MHz, DMSO-d6, 298 K): δ 169.9, 158.8, 154.7, 138.1, 123.6, 112.5, 110.4, 96.9, 80.9, 64.7, 21.9, 12.1; **FT-IR** (thin film): 2932, 1742, 1587, 1427, 1293, 1247, 1163, 1073, 1017, 962, 931, 829, 676, 583, 530; **HRMS** (MALDI/ESI) m/z: $[M+Na]^+$ Calcd for $C_{88}H_{72}NaO_{32}$ 1663.3899; Found 1663.3891. **TLC**: R_f = 0 (20% MeOH/DCM, p-anisaldehyde); **mp:** slow decomposition above 200 °C.

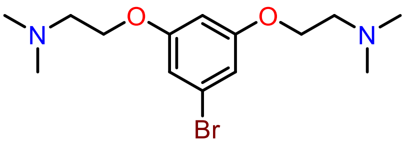

2,2'-((5-bromo-1,3-phenylene)bis(oxy))bis(*N*,*N*-dimethylethan-1-amine) (E)

To a solution of **A** (0.5 g, 2.64 mmol) in EtOH/Toluene 1:1 (50 mL, 0.05 M) is added K_2CO_3 (2.92 g, 21.2 mmol, 8 equiv) and 2-dimethylaminoethyl chloride hydrochloride (1.52 g, 10.6 mmol, 4 equiv) is added. The reaction mixture is stirred at 85 °C for 3 h in an oil bath. The solvent is then removed under reduced pressure. The resulting solid is picked up in DCM and washed once with a 1 M NaOH solution. The organic phase is dried under Na_2SO_4, filtered, and organic solvent removed under reduced pressure to give the title compound as a red oil (747 mg, 2.26 mmol, 85%).

¹H-NMR (500 MHz, $CDCl_3$, 298 K): δ 6.67 (d, J = 2.2 Hz, 2H), 6.43 (t, J = 2.2 Hz, 1H), 4.00 (t, J = 5.7 Hz, 4H), 2.70 (t, J = 5.6 Hz, 4H), 2.32 (s, 12H); **¹³C-NMR {¹H}** (126 MHz, $CDCl_3$, 298 K): δ 160.5, 123.0, 110.8, 101.0, 66.3, 58.2, 45.9; **FT-IR** (thin film): 2941, 2863, 2819, 2769, 1596, 1574, 1438, 1407, 1365, 1331, 1278, 1165, 1097, 1036, 988, 959, 909, 813, 676 cm^{-1}; **HRMS** (ESI) m/z: $[M+H]^+$ Calcd for $C_{14}H_{24}BrN_2O_2$ 331.1016; Found 331.1021. **TLC**: R_f = 0.26 (20:1 DCM/MeOH with 1% Et_3N, UV at 254 nm).

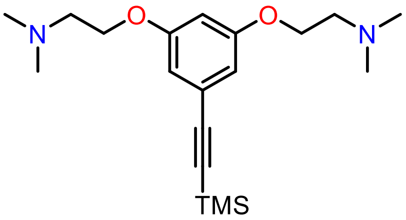

2,2'-((5-((trimethylsilyl)ethynyl)-1,3-phenylene)bis(oxy))bis(*N*,*N*-dimethylethan-1-amine) (F)

To a solution of **E** (1.20 g, 3.62 mmol) in freshly distilled Et_3N (36.2 mL, 100 mM), kept over 5A MS, is added [Pd(PPh_3)$_4$] (209 mg, 181 µmol, 0.05 equiv), CuI (34.5 mg, 181 µmol, 0.05 equiv) and trimethylsilyl acetylene (1.0 mL, 7.25 mmol, 2 equiv). After subjecting the mixture to three cycles of freeze-pump-thaw degassing, the latter is stirred at 70 °C overnight in an oil bath. After cooling the mixture to room temperature, the latter is diluted with DCM and extracted once with brine. The organic phase is dried under $MgSO_4$, filtered and concentrated *in vacuo*. Flash column chromatography (SiO_2; DCM-MeOH gradient with 1% Et_3N on Büchi Reveleris X2) yields the title compound as a yellow oil (1.15 g, 3.30 mmol, 91%).

¹H-NMR (500 MHz, $CDCl_3$, 298 K): δ 6.62 (d, J = 2.3 Hz, 2H), 6.50 (t, J = 2.3 Hz, 1H), 4.02 (t, J = 5.7 Hz, 4H), 2.70 (t, J = 5.6 Hz, 4H), 2.32 (s, 12H), 0.24 (s, 9H); **¹³C-NMR {¹H}** (126 MHz, $CDCl_3$, 298 K): δ 159.8, 124.3, 110.6, 105.2, 103.6, 93.8, 66.3, 58.3, 46.0, 0.1; **FT-IR** (thin film): 2945, 2820, 2769, 2157, 1586, 1456, 1430, 1408, 1348, 1296, 1249, 1159, 1090, 1054, 1042, 1008, 987, 960, 842, 789, 760, 698, 681, 647 cm^{-1}; **HRMS** (ESI) m/z: $[M+H]^+$ Calcd for $C_{19}H_{33}N_2O_2Si$ 349.2306; Found 349.2301. **TLC**: R_f = 0.28 (20:1 DCM/MeOH with 1% Et_3N, p-anysaldehyde).

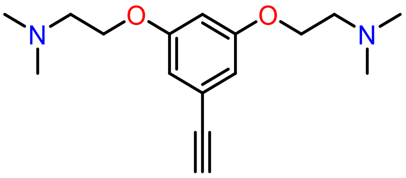

2,2'-((5-ethynyl-1,3-phenylene)bis(oxy))bis(*N*,*N*-dimethylethan-1-amine) (5)

To an ice cold solution of **F** (351 mg, 1.01 mmol) in THF extra dry (1.3 mL, 0.5 M) and under N_2 atmosphere is added dropwise a 1M TBAF in THF solution (1.41 mL, 1.41 mmol, 1.4 equiv). The mixture is warmed to room temperature and stirred for 30 minutes and then quenched with a few drops of water. The organic phase is extracted with a saturated $NaHCO_3$ solution, dried under $MgSO_4$, filtered and concentrated *in vacuo*. Flash column chromatography (SiO_2;

DCM-MeOH gradient with 1% Et_3N on Büchi Reveleris X2) yields the title compound as a yellow oil (274 mg, 0.99 mmol, 98%).

^{1}H-NMR (500 MHz, $CDCl_3$, 298 K): δ 6.64 (d, J = 2.3 Hz, 2H), 6.52 (t, J = 2.3 Hz, 1H), 4.02 (t, J = 5.7 Hz, 4H), 2.71 (t, J = 5.6 Hz, 4H), 3.02 (s, 1H), 2.33 (s, 12H); **^{13}C-NMR {^{1}H}** (126 MHz, $CDCl_3$, 298 K): δ 159.8, 123.4, 110.8, 103.5, 83.7, 76.8, 66.2, 58.3, 45.99, 45.95; **FT-IR** (thin film): 3287, 2970, 2942, 2865, 2820, 2770, 2104, 1586, 1456, 1432, 1408, 1366, 1344, 1321, 1294, 1165, 1098, 1054, 1041, 1004, 959, 848, 789, 681, 653, 603 cm^{-1}; **HRMS** (ESI) m/z: $[M+H]^+$ Calcd for $C_{16}H_{25}N_2O_2$ 277.1911; Found 277.1917. **TLC**: R_f = 0.3 (10:1 DCM/MeOH with 1% Et_3N, p-anysaldehyde).

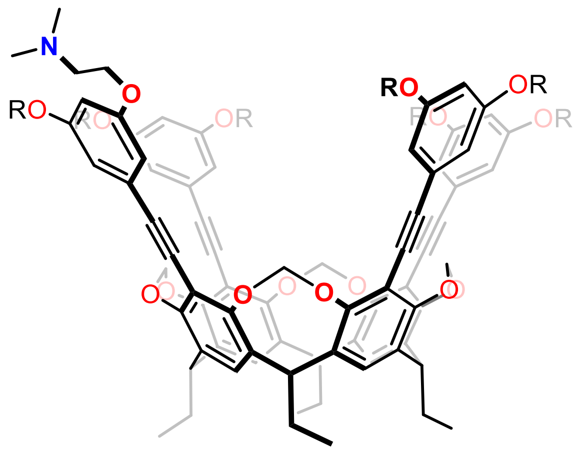

R = $CH_2CH_2N(CH_3)_2$

Cavitand 8

To a solution of **3** (569 mg, 494 µmol) in freshly distilled Et_3N (20 mL, 25 mM), kept over 5A MS, is added $[Pd(PPh_3)_4]$ (57 mg, 49.4 µmol, 0.1 equiv), CuI (9.4 mg, 53.6 µmol, 0.1 equiv) and alkyne **5** (683 mg, 2.47 mmol, 5 equiv). After subjecting the mixture to three cycles of freeze-pump-thaw degassing, the latter is stirred at 70 °C overnight in an oil bath. The solvent is concentrated *in vacuo* and the remaining liquid is diluted with DCM and extracted once with a 1 M HCl solution. The aqueous phase is washed twice with DCM and then neutralized/basified through extraction with a 6M NaOH solution. The organic phase is dried under $MgSO_4$, filtered on a pad of celite and concentrated *in vacuo*. The obtained solid is washed 6 times with hexane and once with pentane. It is then dissolved in $CHCl_3$ and concentrated *in vacuo* to yield the cavitand **8** as a white solid (613 mg, 494 µmol, quantitative).

^{1}H-NMR (500 MHz, $CDCl_3$, 298 K): δ 7.09 (s, 4H), 6.62 (d, J = 2.3 Hz, 8H), 6.50 (t, J = 2.3 Hz, 4H), 6.01 (d, J = 7.2 Hz, 4H), 4.77 (t, J = 8.1 Hz, 4H), 4.60 (d, J = 7.3 Hz, 4H), 4.00 (t, J = 5.7 Hz, 16H), 2.67 (t, J = 5.7 Hz, 17H), 2.28 (s, 58H), 1.03 (t, J = 7.2 Hz, 12H); **^{13}C-NMR {^{1}H}** (126 MHz, $CDCl_3$, 298 K): δ 159.9, 155.6, 138.4, 124.3, 120.1, 113.3, 110.6, 102.9, 98.8, 97.8, 80.7, 66.2, 58.3, 45.97, 38.7, 22.9, 12.4; **FT-IR** (thin film): 2938, 2766, 1581, 1444, 1393, 1354, 1294, 1164, 1093, 1042, 1018, 996, 971, 843, 813, 754, 678; **HRMS** (ESI-QTOF) m/z: $[M+H]^+$ Calcd for

$C_{104}H_{129}N_8O_{16}$ 1745.9521; Found 1745.9506. **TLC**: R_f = 0 (20% MeOH/DCM, p-anisaldehyde stain); **mp:** 171-173 °C.

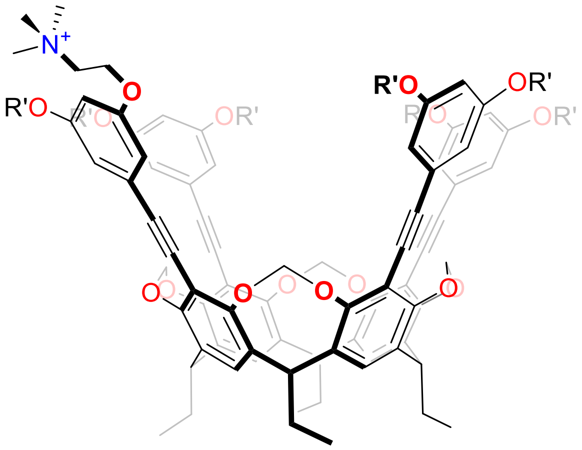

Cavitand 9

To a solution of cavitand **8** (101 mg, 58.0 µmol) in DMF extra dry (1 mL, 58 mM) at room temperature is added methyl iodide (1.1 mL, 17.4 mmol, 300 equiv). A precipitate forms immediately. The mixture is then stirred at room temperature for 2 days. The solvent/MeI is then removed under a N_2 flow. The obtained wet solid is suspended and sonicated in hexane and subsequently decanted. This procedure is repeated twice with hexane and twice with ACN. Removal of the solvent under reduced pressure yields cavitand **9** as a white solid (130 mg, 45 µmol, 80%).

^{1}H-NMR (500 MHz, DMSO-d6, 298 K): δ 7.69 (s, 4H), 6.79 (d, J = 2.2 Hz, 8H), 6.76 (t, J = 2.3 Hz, 4H) 6.18 (d, J = 7.5 Hz, 4H), 4.58 (t, J = 8.1 Hz, 4H), 4.52 (t, J = 4.7 Hz, 16H), 4.45 (d, J = 7.5 Hz, 4H), 3.79 (t, J = 4.8 Hz, 16H), 3.21 (s, 72H), 2.48 – 2.41 (m, 8H), 0.97 (t, J = 7.1 Hz, 12H); **^{13}C-NMR {^{1}H}** (126 MHz, DMSO-d6, 298 K): δ 158.5, 154.7, 138.2, 123.8, 122.9, 112.2, 111.1, 103.5, 96.5, 81.3, 64.0, 62.3, 53.3, 39.5, 22.0, 12.1; **FT-IR** (thin film): 3431, 2958, 1584, 1472, 1449, 1346, 1294, 1165, 1068, 962, 878, 680, 583, 532; **mp:** slow decomposition above 250 °C.

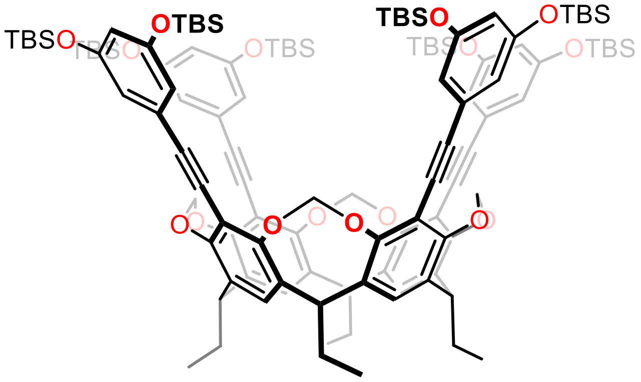

Octa-TBS Cavitand (G)

To a solution of **3** (173 mg, 166 µmol) in freshly distilled Et_3N (6 mL, 25 mM), kept over 5A MS, is added [$Pd(PPh_3)_4$] (8.4 mg, 33.2 µmol, 0.2 equiv), CuI (6.32 mg, 33.2 µmol, 0.2 equiv) and the commercially available TBS-phenylacetylene [2] (47.2 mg, 682 µmol, 4.1 equiv). After subjecting the mixture to three cycles of freeze-pump-thaw degassing, the latter is stirred at 100 °C overnight in an oil bath. The solvent is concentrated *in vacuo* and the remaining liquid is diluted with DCM and extracted once with a saturated aq. NH_4Cl solution. The organic phase is dried under $MgSO_4$, filtered, and concentrate *in vacuo*. Flash column chromatography (SiO_2; Hexane/DCM gradient on Büchi Reveleris X2) yields the title compound as a white solid (219 mg, 105 µmol, 70%).

^{1}H-NMR (500 MHz, $CDCl_3$, 298 K): δ 7.09 (s, 4H), 6.52 (d, J = 2.2 Hz, 8H), 6.28 (t, J = 2.2 Hz, 4H), 6.00 (d, J = 7.2 Hz, 4H), 4.77 (t, J = 8.1 Hz, 4H), 4.62 (d, J = 7.2 Hz, 4H), 2.28 (p, J = 7.4 Hz, 8H), 1.03 (t, J = 7.2 Hz, 12H), 0.91 (s, 72H), 0.12 (s, 48H).**^{13}C-NMR {^{1}H}** (126 MHz, $CDCl_3$, 298 K): δ 156.6, 155.6, 138.4, 124.0, 120.1, 116.7, 113.5, 113.4, 98.6, 97.9, 80.6, 77.4, 77.2, 76.9, 38.7, 25.7, 22.9, 18.3, 12.4, -4.3; **FT-IR** (thin film): 2931, 1757, 1578, 1470, 1446, 1426, 1392, 1359, 1300, 1253, 1162, 1093, 1073, 1022, 998, 974, 900, 883, 827, 780, 741, 679, 583, 536; **HRMS** (MALDI/ESI) m/z: $[M+Na]^+$ Calcd for $C_{120}H_{168}NaO_{16}Si_8$ 2112.0379; Found 2112.0362. **TLC**: R_f = 0.6 (10:1 Hexane/EtOAc, p-anisaldehyde stain); **mp:** 303 °C.

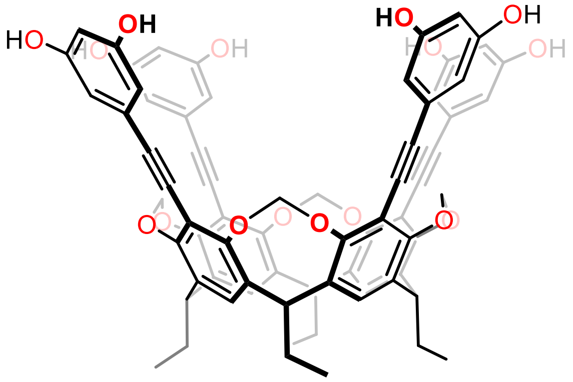

Octa-Phenol Cavitand (H)

To an ice-cold solution of **G** (397 mg, 190 µmol) in THF (2.9 mL, 65 mM) and under N_2 atmosphere is added dropwise a 1M TBAF in THF solution (1.6 mL, 1.6 mmol, 8.5 equiv). The

mixture is warmed to room temperature and stirred for 30 minutes and then quenched with a few drops of a saturated aq. NH_4Cl solution. The organic phase is extracted with brine, dried under $MgSO_4$, filtered, and concentrated *in vacuo*. A 0.1 M NaOH solution is then added and the aqueous phase is washed twice with EtOAc, acidified with a 0.1 M HCl solution to pH 2 and then extracted twice with EtOAc. The organic phase is then washed twice with distilled water, dried under $MgSO_4$, filtered, and solvent removed under reduced pressure to yield the title compound as a white solid (219 mg, 186 µmol, 98%).

^{1}H-NMR (500 MHz, DMSO-d6, 298 K): δ 9.45 (s, 8H), 7.62 (s, 4H), 6.33 (d, J = 2.2 Hz, 8H), 6.23 (t, J = 2.2 Hz, 4H), 6.00 (d, J = 7.4 Hz, 4H), 4.57 (t, J = 8.1 Hz, 4H), 4.47 (d, J = 7.5 Hz, 4H), 2.44 (q, J = 7.5 Hz, 8H), 2.07 (s, 5H), 0.98 (t, J = 7.2 Hz, 12H); **^{13}C-NMR {^{1}H}** (126 MHz, DMSO-d6, 298 K): δ 158.4, 154.5, 138.2, 123.1, 122.3, 112.7, 109.5, 104.0, 97.9, 97.7, 79.6, 39.5, 21.9, 12.0; **FT-IR** (thin film): 3274, 2962, 1746, 1593, 1505, 1446, 1395, 1354, 1298, 1150, 1093, 1074, 1021, 998, 970, 841, 740, 676, 573, 523; **HRMS** (MALDI/ESI) m/z: $[M+Na]^+$ Calcd for $C_{72}H_{56}NaO_{16}$ 1199.3461; Found 1199.3458. **mp:** slow decomposition above 300 °C.

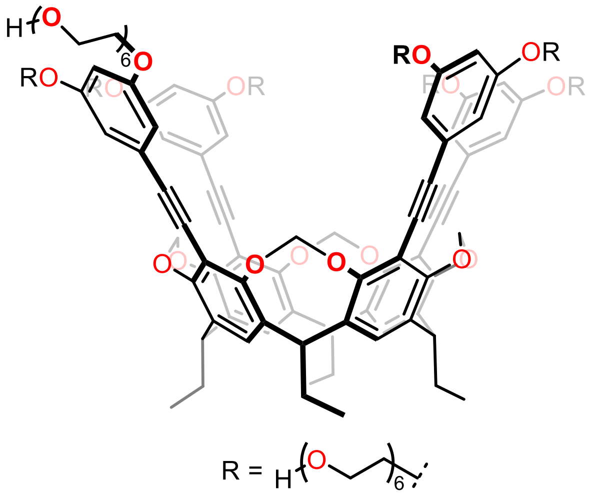

Cavitand 10

To a solution of **H** (100 mg, 85 µmol) in DMF (3.4 mL, 25 mM) and under N_2 atmosphere is added K_2CO_3 (235 mg, 1.7 mmol, 20 equiv) and hexaethylene glycol p-toluenesulfonate (371 mg, 849 µmol, 10 equiv). The mixture is stirred at 80 °C for 3 days in an oil bath. The solvent is concentrated *in vacuo* and the remaining liquid is diluted with DCM and extracted once with a saturated aq. NH_4Cl solution. The organic phase is dried under $MgSO_4$, filtered, and concentrated *in vacuo*. Flash column chromatography (C_{18} SiO_2; Water/Methanol gradient on Büchi Reveleris X2) yields cavitand **10** as a colourless oil (167 mg, 51 µmol, 60%).

^{1}H-NMR (500 MHz, $CDCl_3$, 298 K): δ 7.09 (s, 4H), 6.61 (d, J = 2.2 Hz, 8H), 6.49 (t, J = 2.3 Hz, 4H), 6.01 (d, J = 7.2 Hz, 4H), 4.75 (t, J = 8.1 Hz, 4H), 4.58 (d, J = 7.3 Hz, 4H), 4.07 (t, J = 4.9 Hz, 16H), 3.83 – 3.77 (m, 16H), 3.74 – 3.62 (m, 152H), 3.62 – 3.56 (m, 16H), 2.27 (q, J = 7.5 Hz, 8H), 1.02 (t, J = 7.2 Hz, 12H); **^{13}C-NMR {^{1}H}** (126 MHz, $CDCl_3$, 298 K): δ 159.8, 155.6, 138.4, 124.4, 120.2, 113.3, 110.7, 103.0, 98.8, 97.7, 80.9, 77.2, 72.7, 70.9, 70.7, 70.7, 70.7, 707, 70.5, 69.7, 67.7, 61.8, 38.7, 22.9, 12.4; **FT-IR** (thin film): 3458, 2871, 1743, 1687, 1584, 1448, 1399, 1349, 1326,

1294, 1249, 1166, 1096, 1068, 1016, 966, 845, 739, 682, 644, 582, 529; **HRMS** (MALDI/ESI) m/z: $[M+Na]^+$ Calcd for $C_{168}H_{248}NaO_{64}$ 3312.6044; Found 3312.6016.

3. Synthetic Schemes

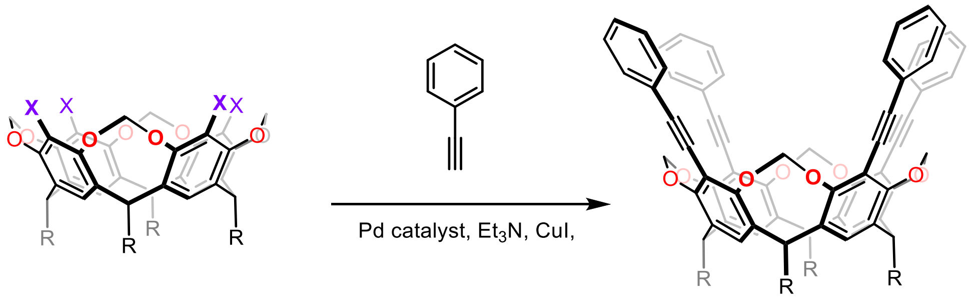

Scheme S1: Synthetic protocol reported by Aekeröy *et al*. for the synthesis of organic-soluble cavitand(s)

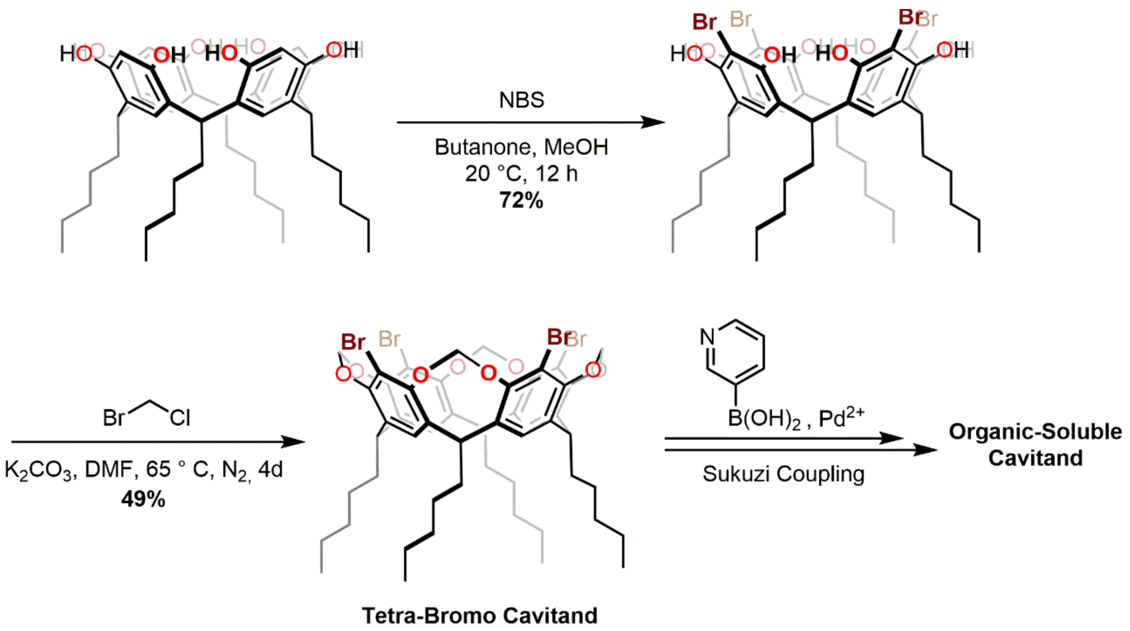

Scheme S2: Synthetic protocol reported by Aekeröy *et al*. for the synthesis of organic-soluble cavitand(s). [3]

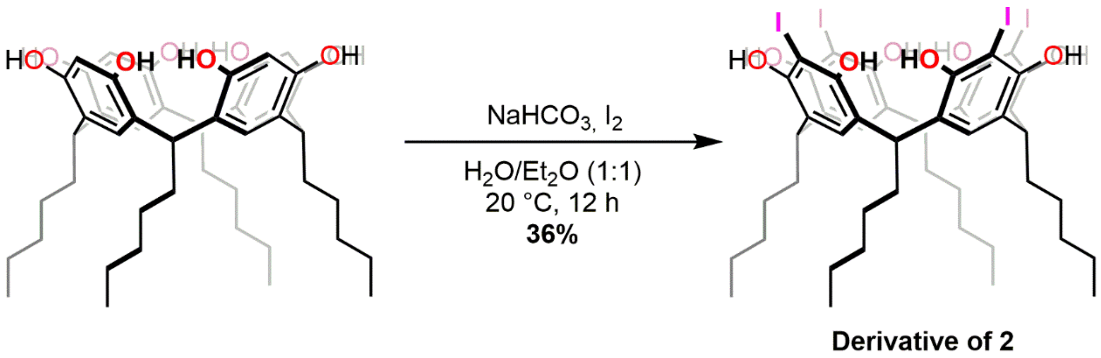

Scheme S3: Synthetic protocol reported by Dalcanale *et al*. for the iodination of resorcin[4]arenes. [4]

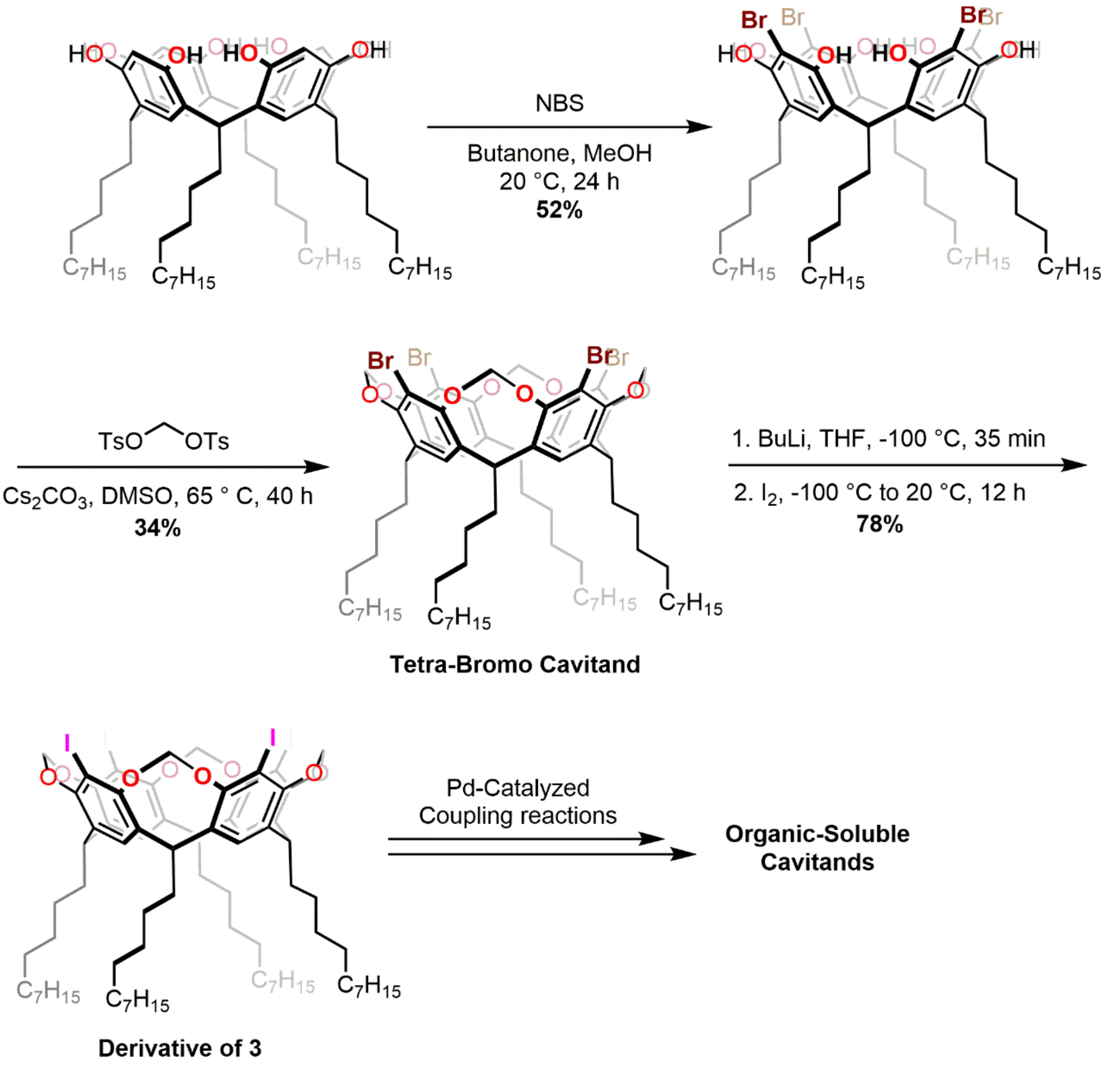

Scheme S4: Synthetic protocol reported by Diederich *et al*. for the synthesis of organic-soluble cavitands. [5]

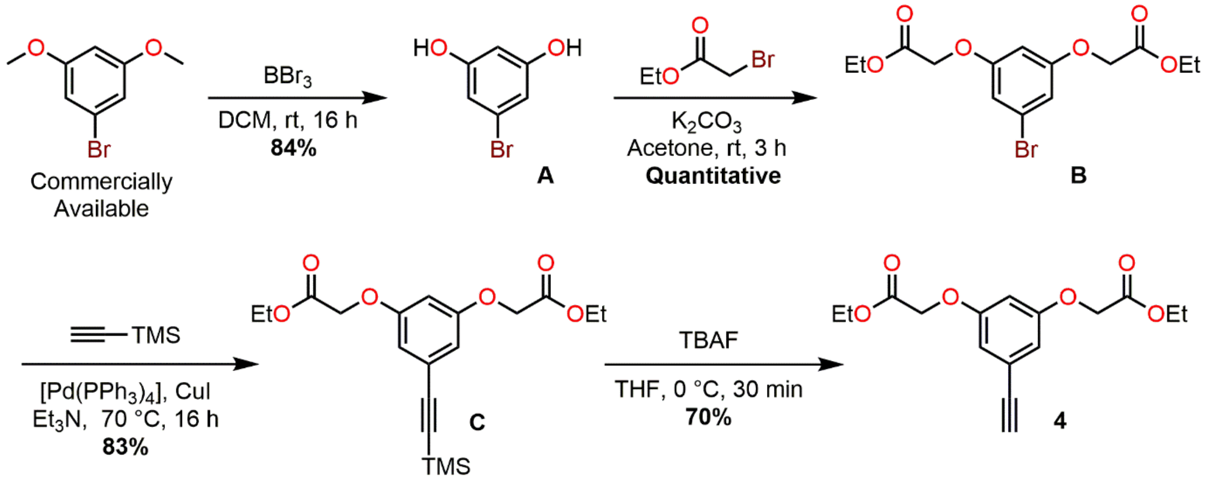

Scheme S5: Synthetic scheme for the synthesis of alkyne **4**.

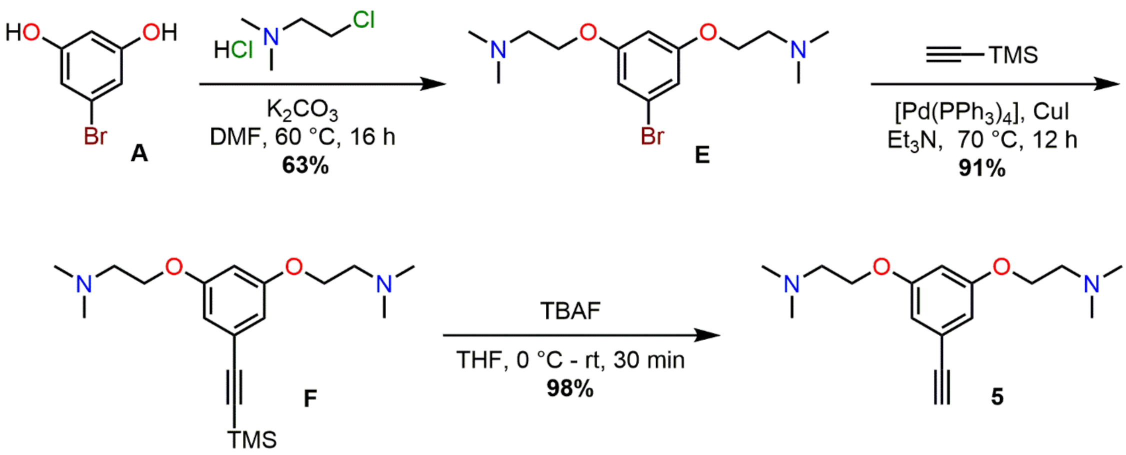

Scheme S6: Synthetic scheme for the synthesis of alkyne **4**.

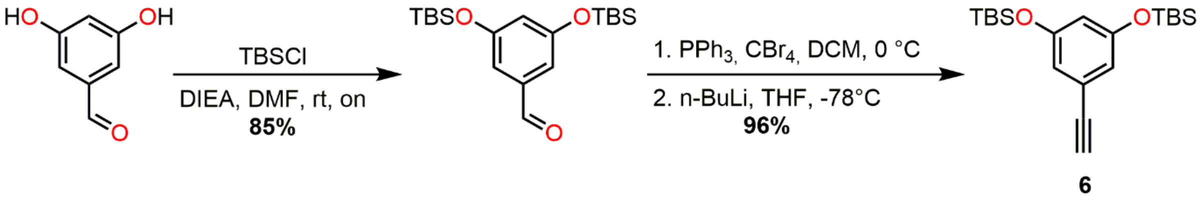

Scheme S7: Synthetic scheme for the synthesis of alkyne **6**.

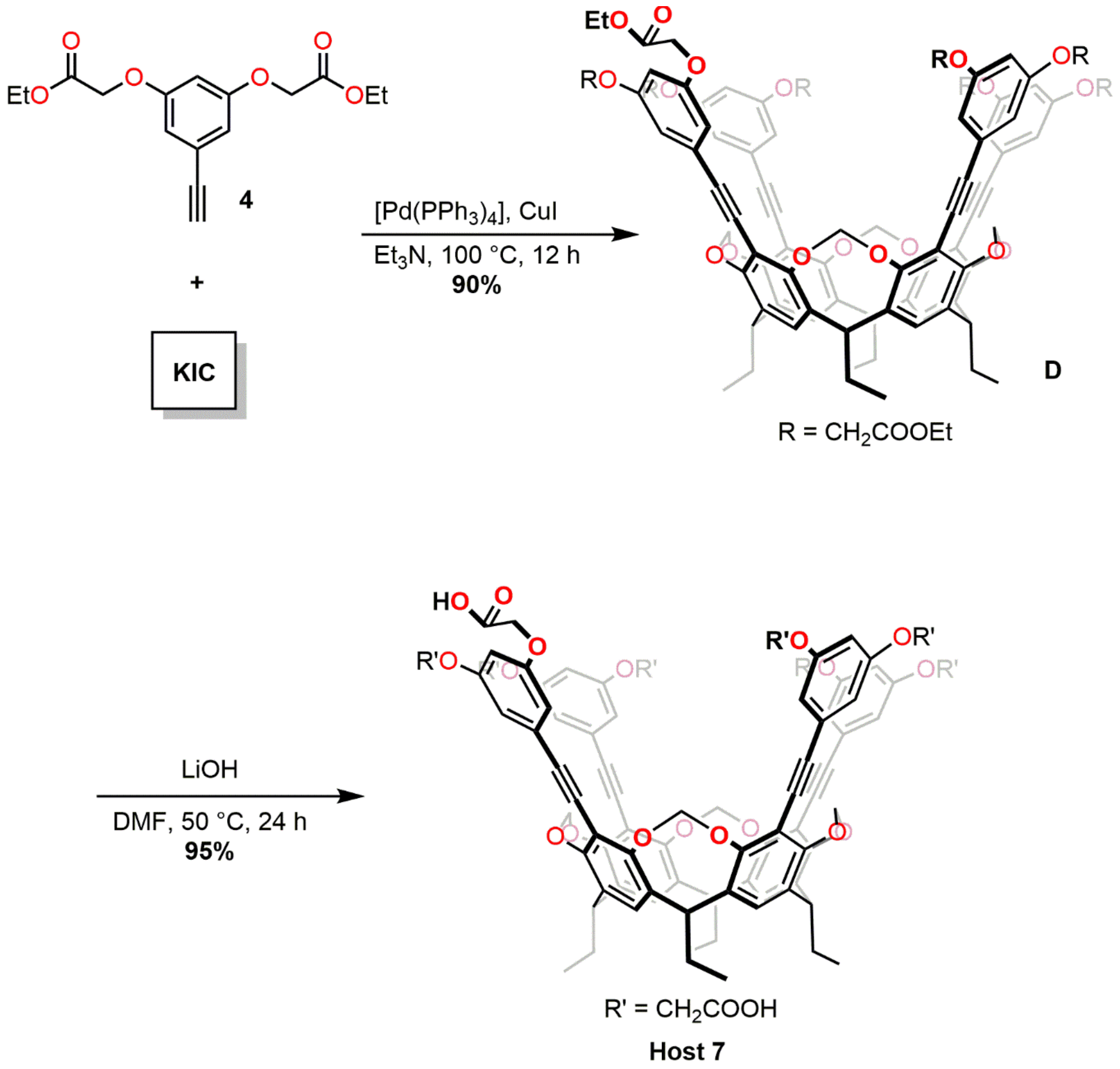

Scheme S8: Synthetic scheme for the synthesis of Host **7**.

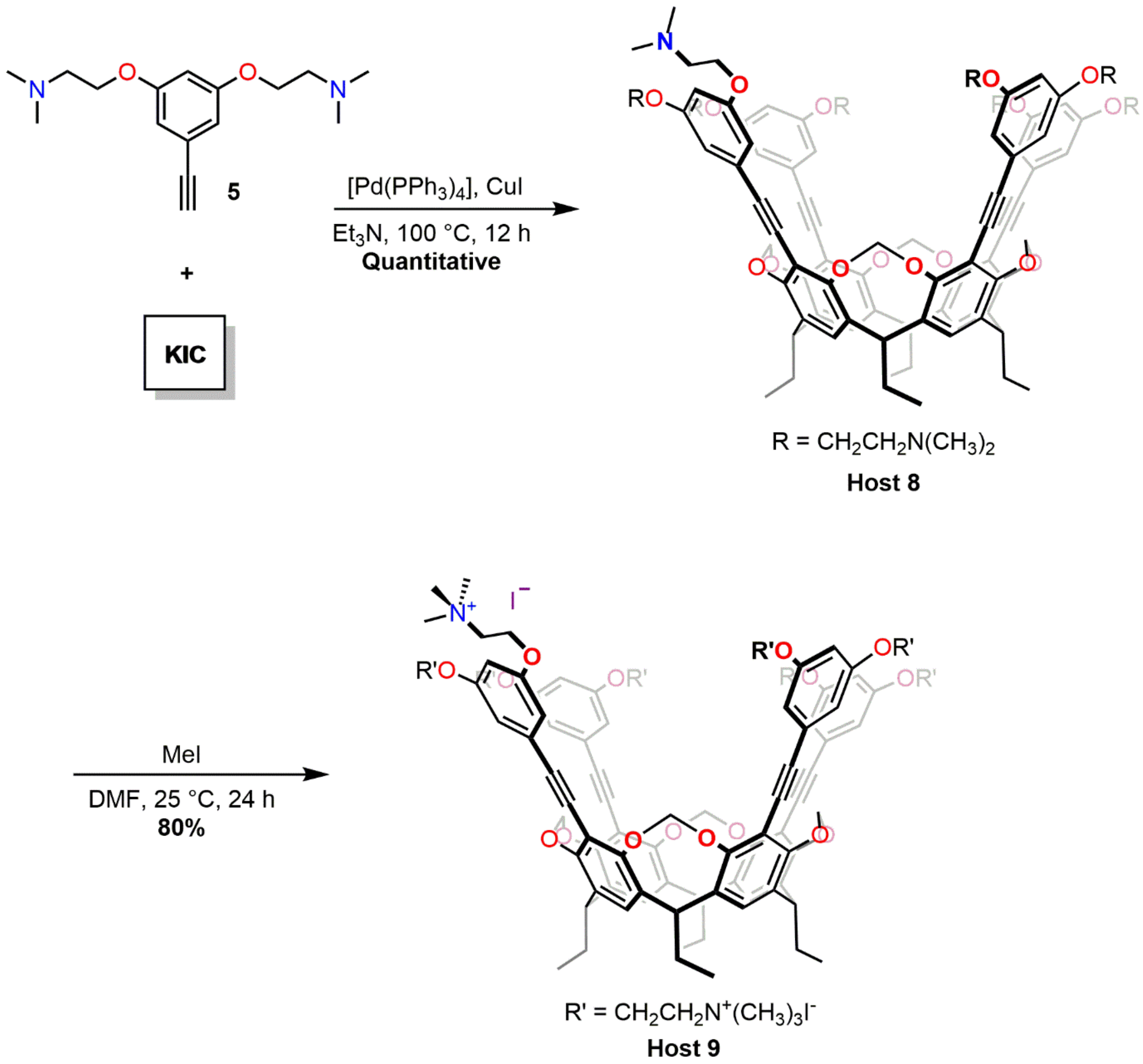

Scheme S9: Synthetic scheme for the synthesis of Host **8** and **9**.

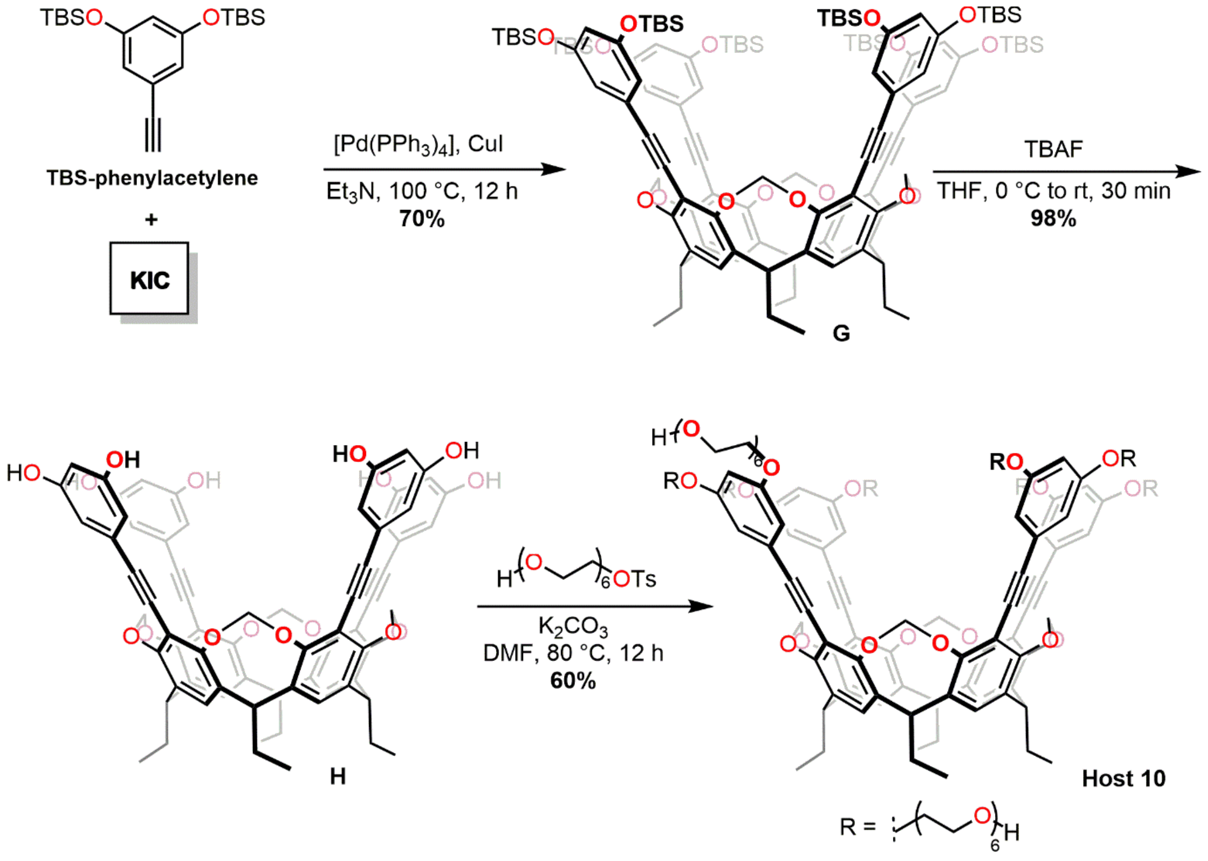

Scheme S10: Synthetic scheme for the synthesis of Host **10**.

4. Spectral Data

4.1 NMR Spectra

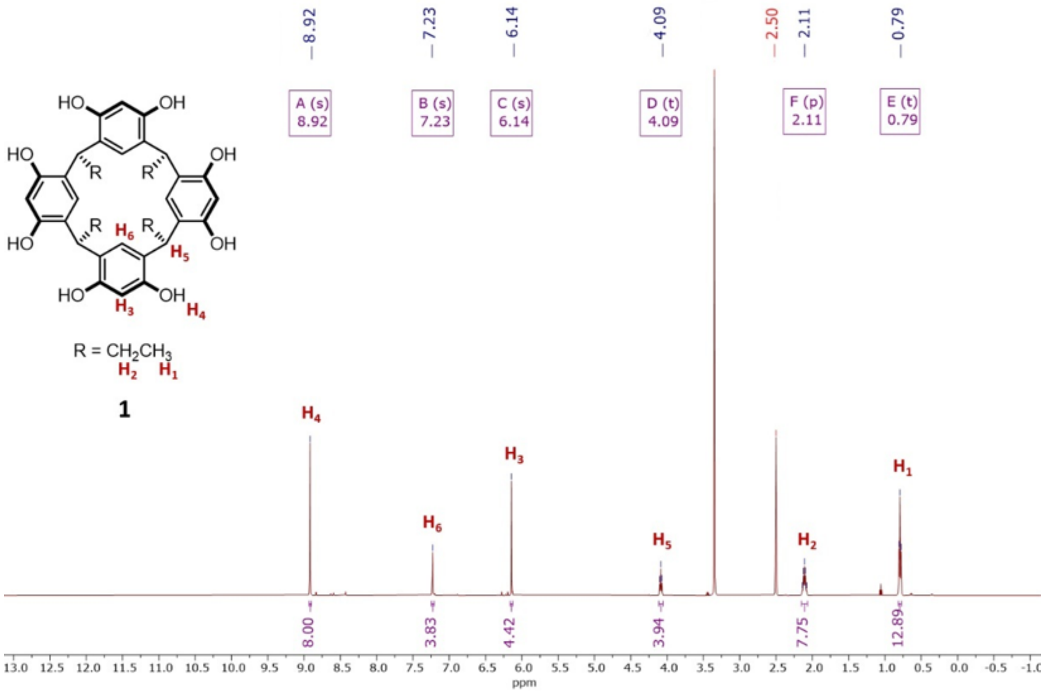

Figure S1: Full ^{1}H NMR (500 MHz, DMSO-d6, 298 K) of compound **1**.

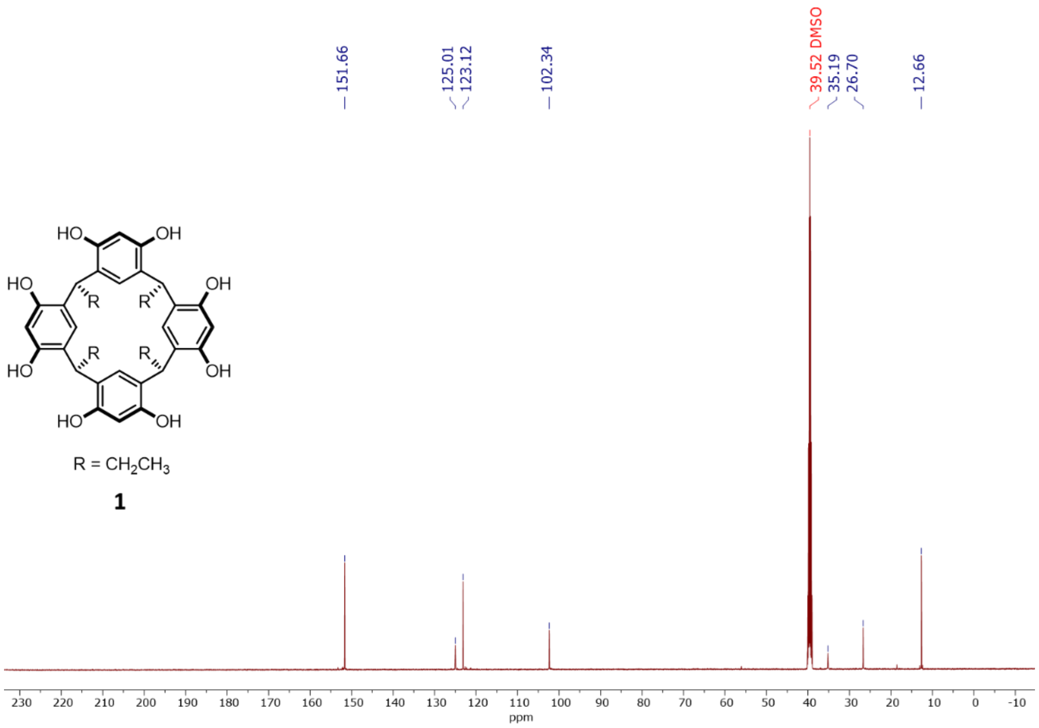

Figure S2: Full ^{13}C {^{1}H} NMR (126 MHz, DMSO-d6, 298 K) of compound **1**.

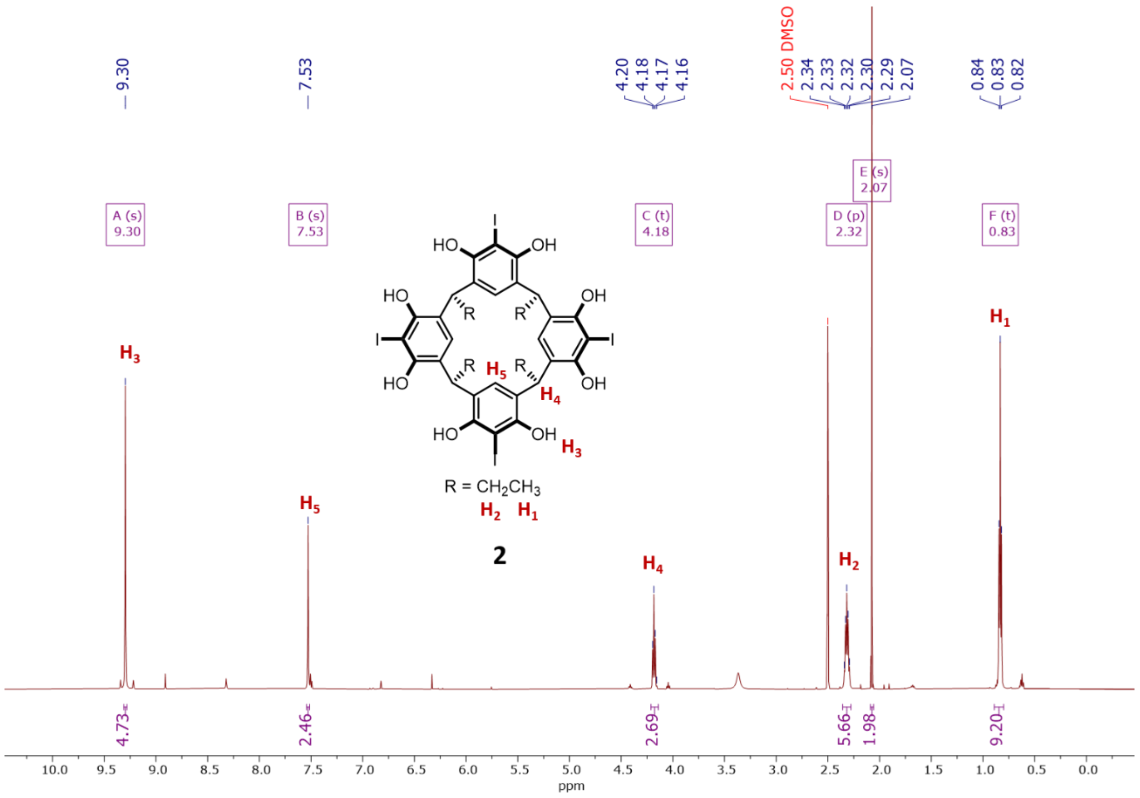

Figure S3: Full ^{1}H NMR (500 MHz, DMSO-d6, 298 K) of compound **2**.

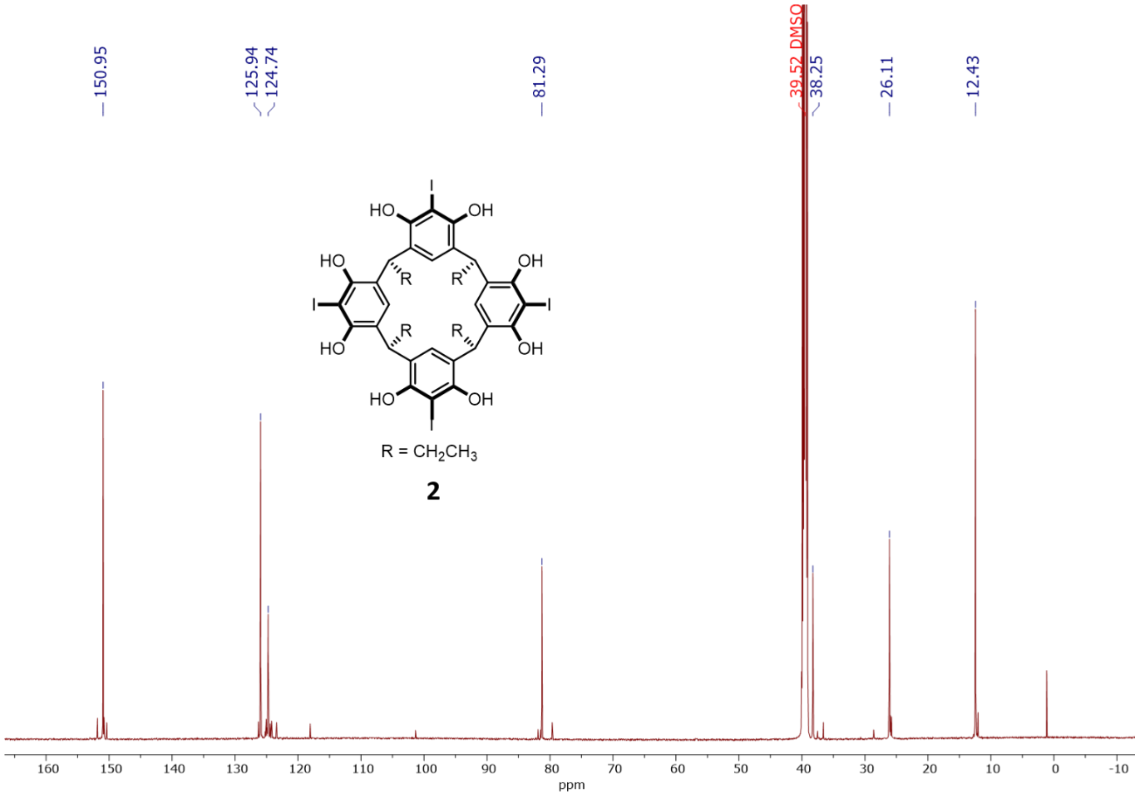

Figure S4: Full ^{13}C NMR {^{1}H} (126 MHz, DMSO-d6, 298 K) of compound **2**.

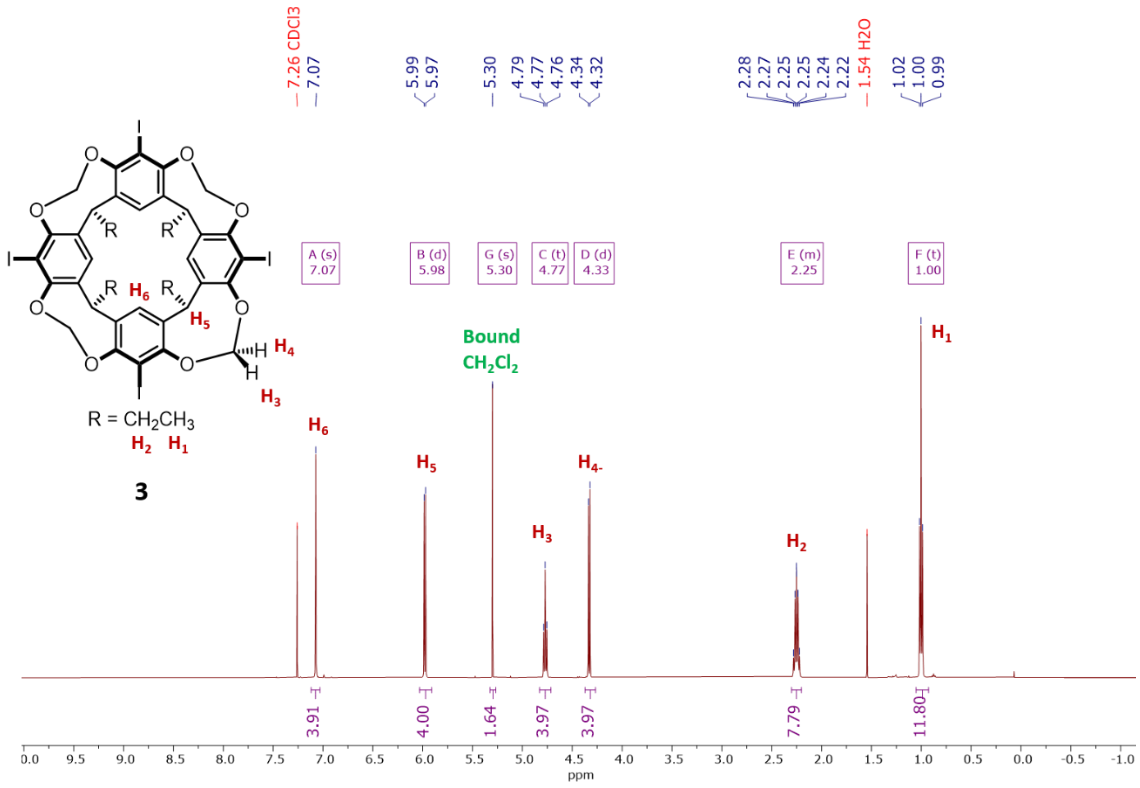

Figure S5: Full ^{1}H NMR (500 MHz, $CDCl_3$, 298 K) of compound **3** (KIC). A molecule of CH_2Cl_2 was encapsulated in the cavity of **3** (δ 5.30 ppm), not removable under *high vacuum*.

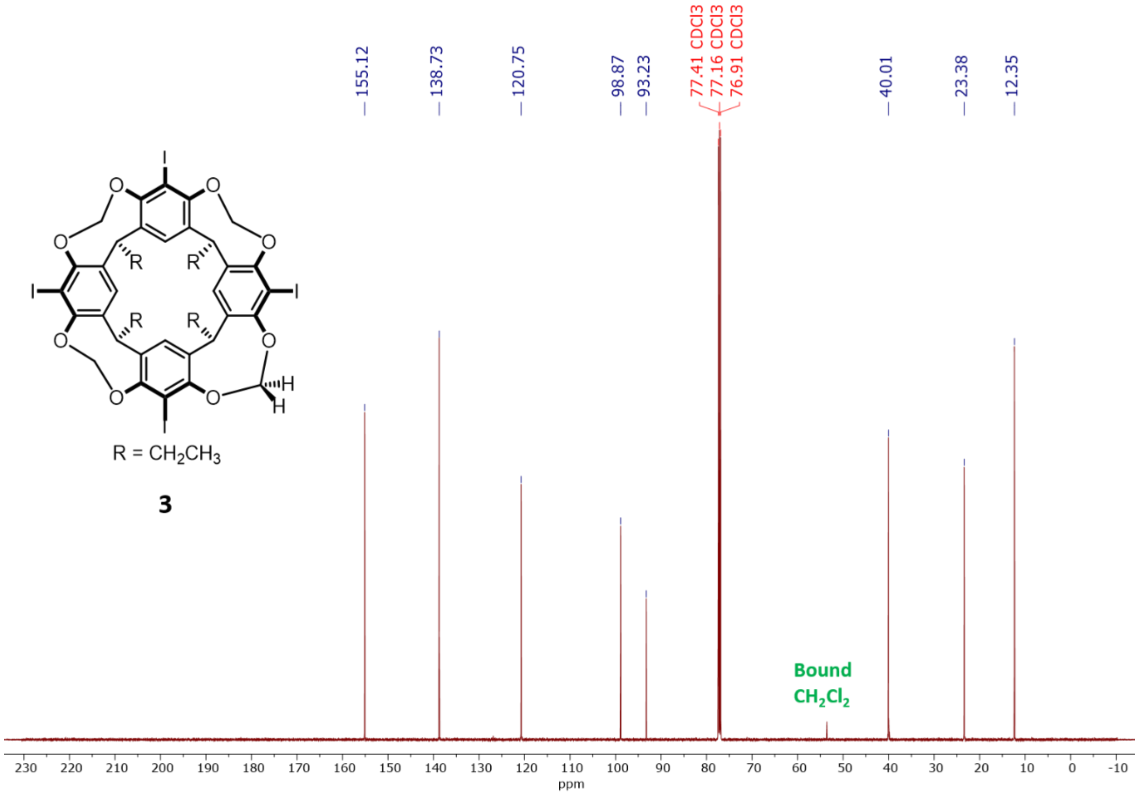

Figure S6: Full ^{13}C NMR {^{1}H} (126 MHz, $CDCl_3$, 298 K) of compound **3** (KIC).

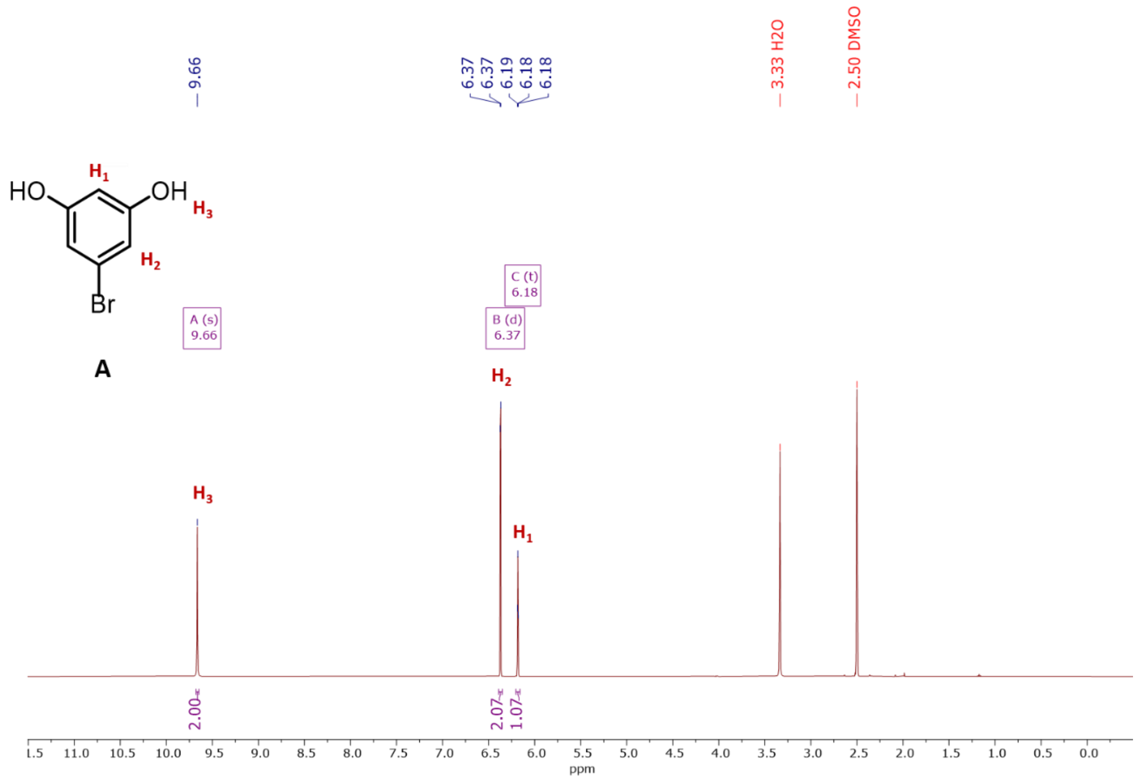

Figure S7: Full ^{1}H NMR (500 MHz, DMSO-d6, 298 K) of compound **A**.

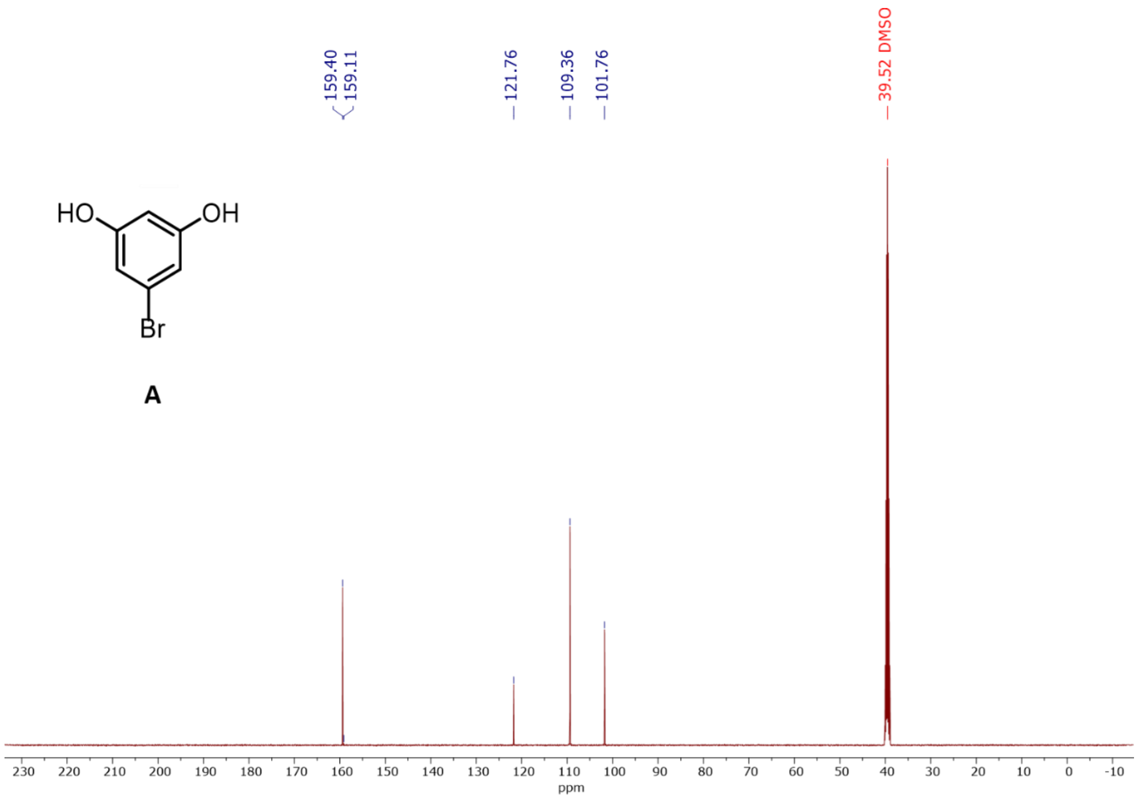

Figure S8: Full ^{13}C NMR {^{1}H} (126 MHz, DMSO-d6, 298 K) of compound **A**.

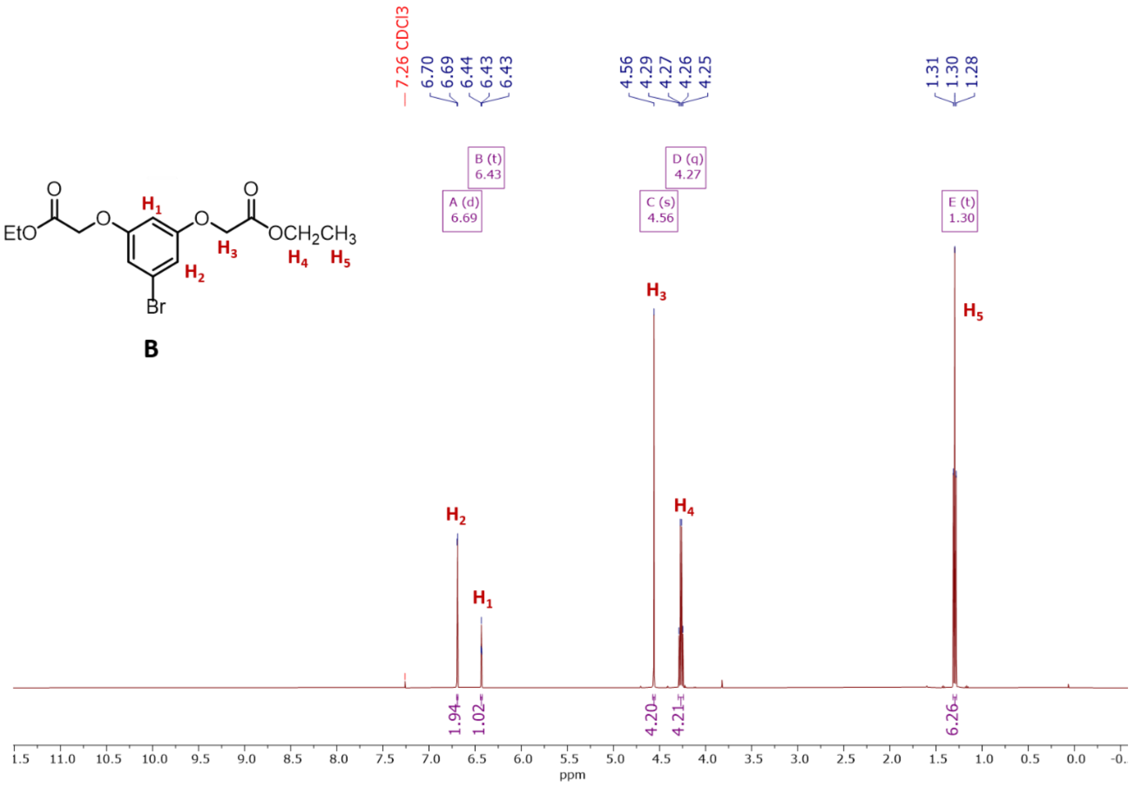

Figure S9: Full ^{1}H NMR (500 MHz, $CDCl_3$, 298 K) of compound **B**.

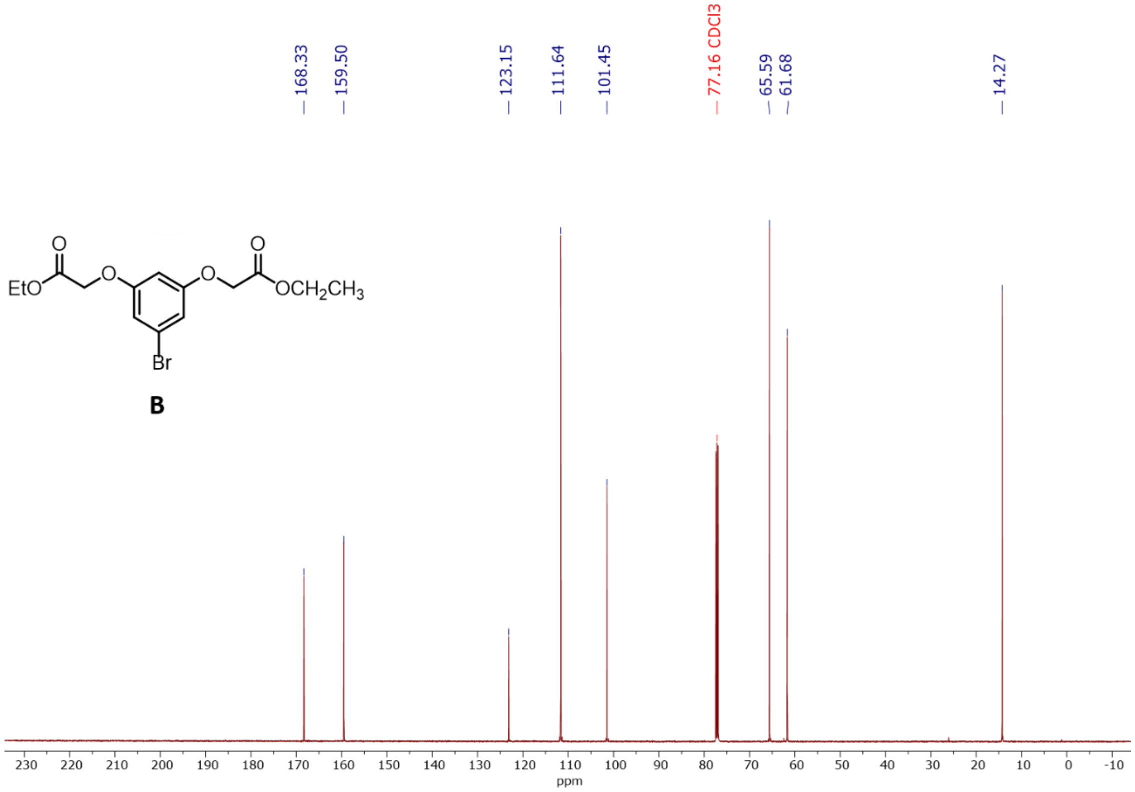

Figure S10: Full ^{13}C NMR {^{1}H} (126 MHz, $CDCl_3$, 298 K) of compound **B**.

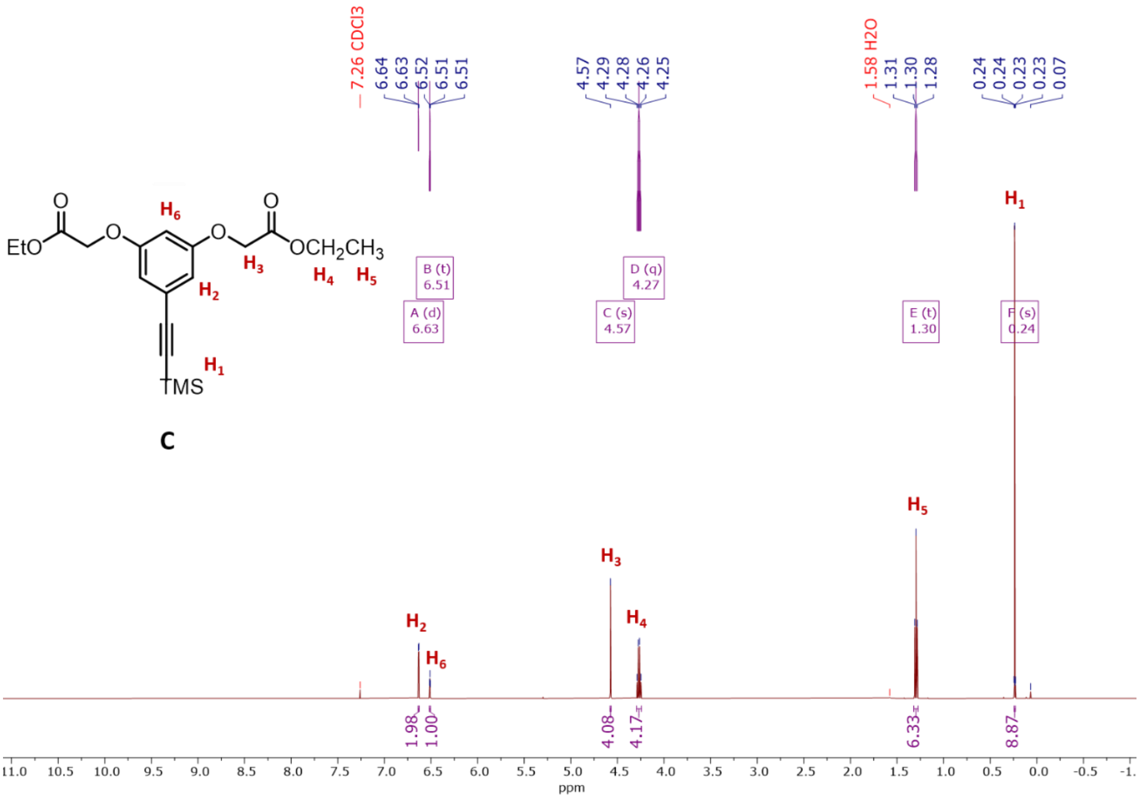

Figure S11: Full ^1H NMR (500 MHz, $CDCl_3$, 298 K) of compound **C**.

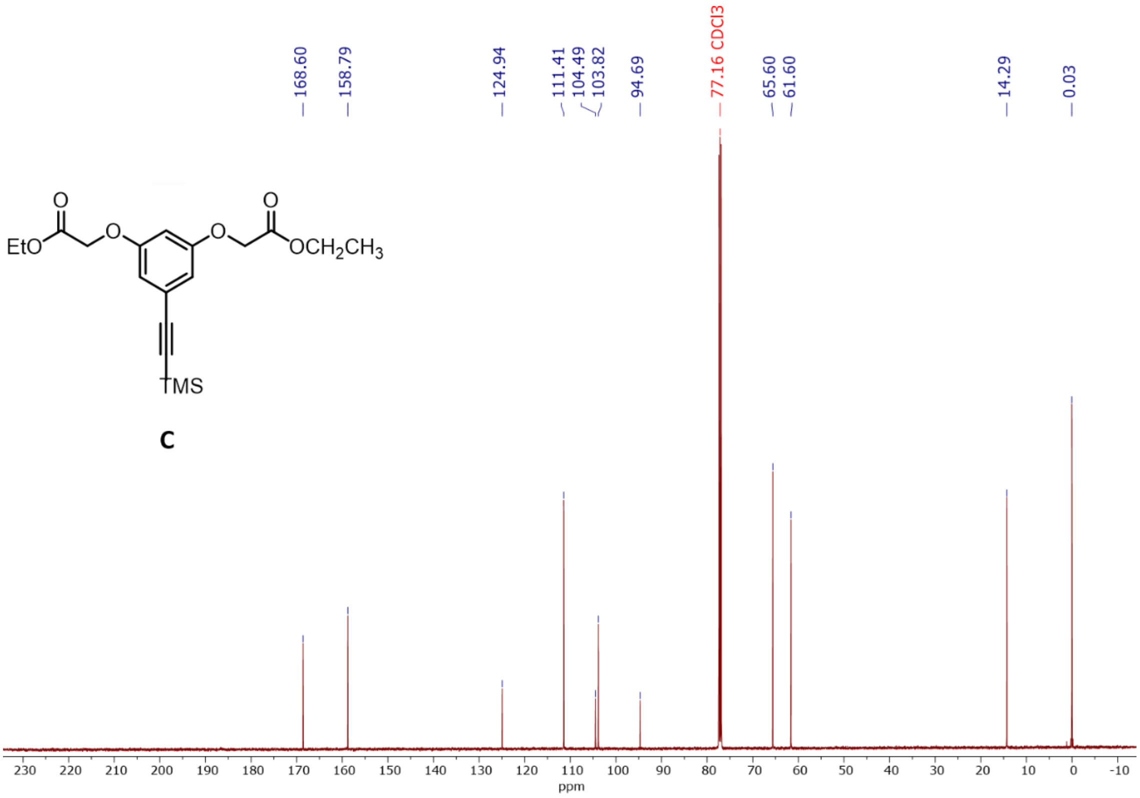

Figure S12: Full ^{13}C NMR {^1H} (126 MHz, $CDCl_3$, 298 K) of compound **C**.

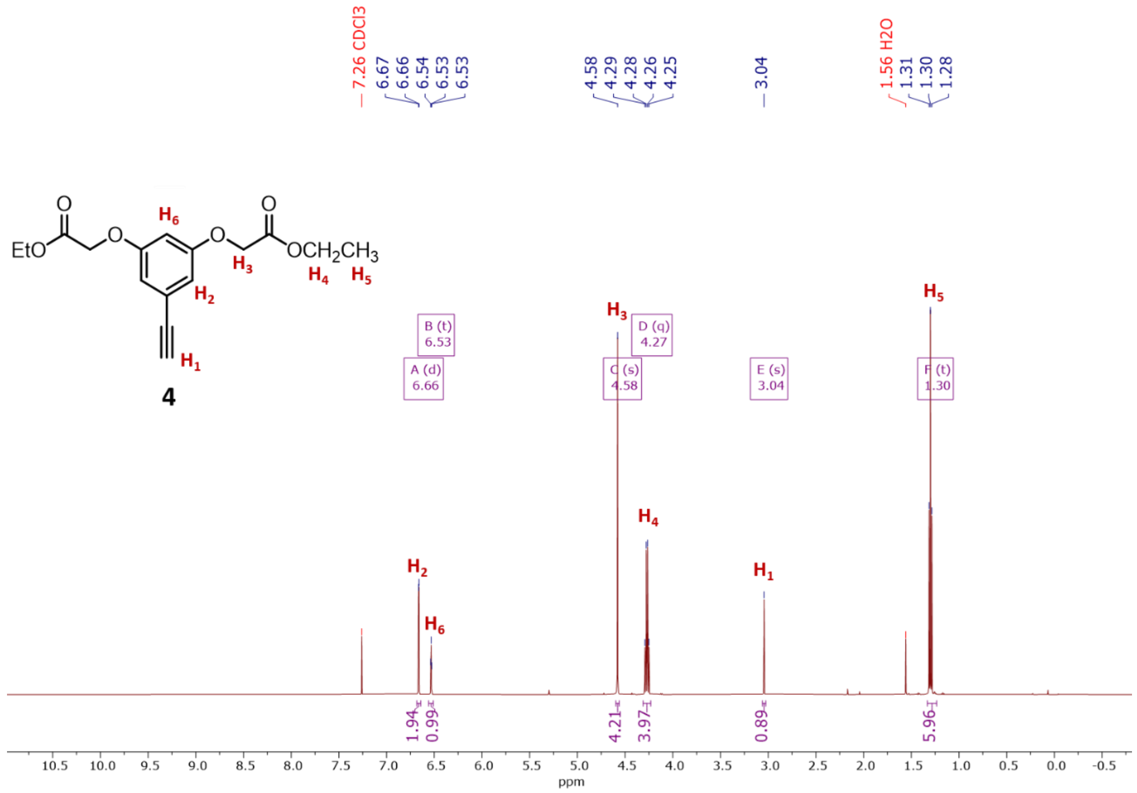

Figure S13: Full ^{1}H NMR (500 MHz, $CDCl_3$, 298 K) of alkyne **4**.

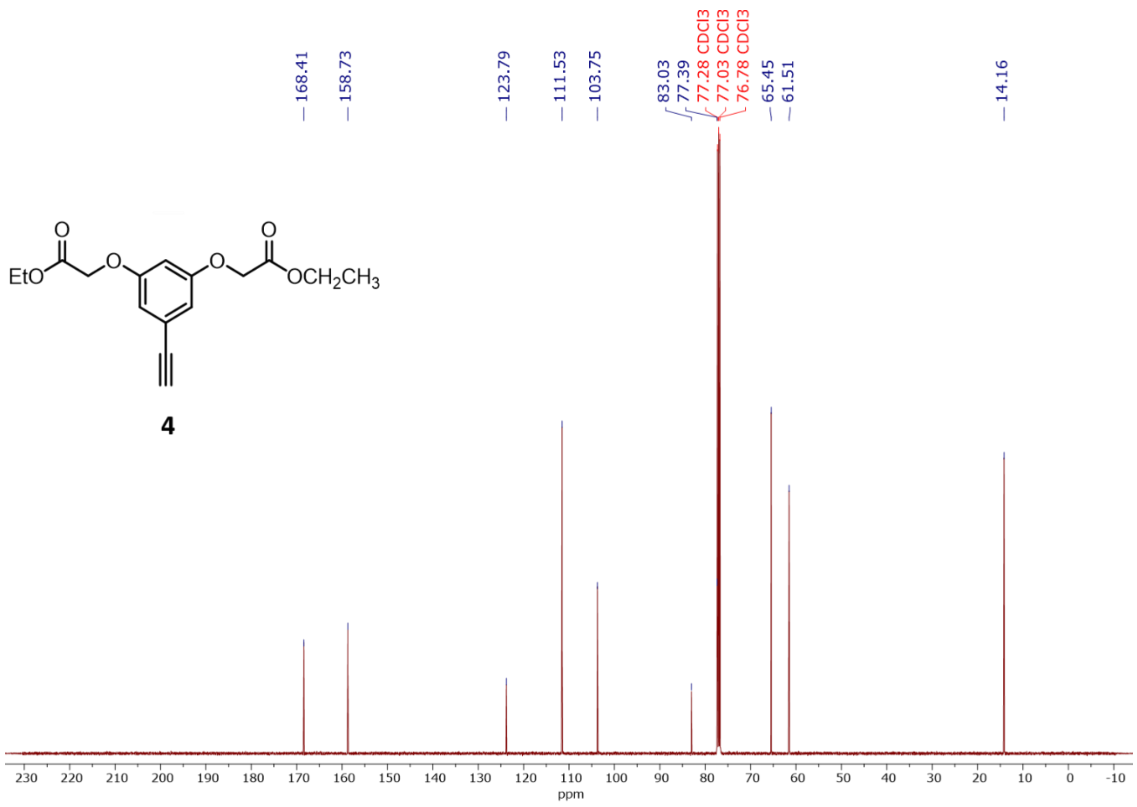

Figure S14: Full ^{13}C NMR {^{1}H} (126 MHz, $CDCl_3$, 298 K) of alkyne **4**.

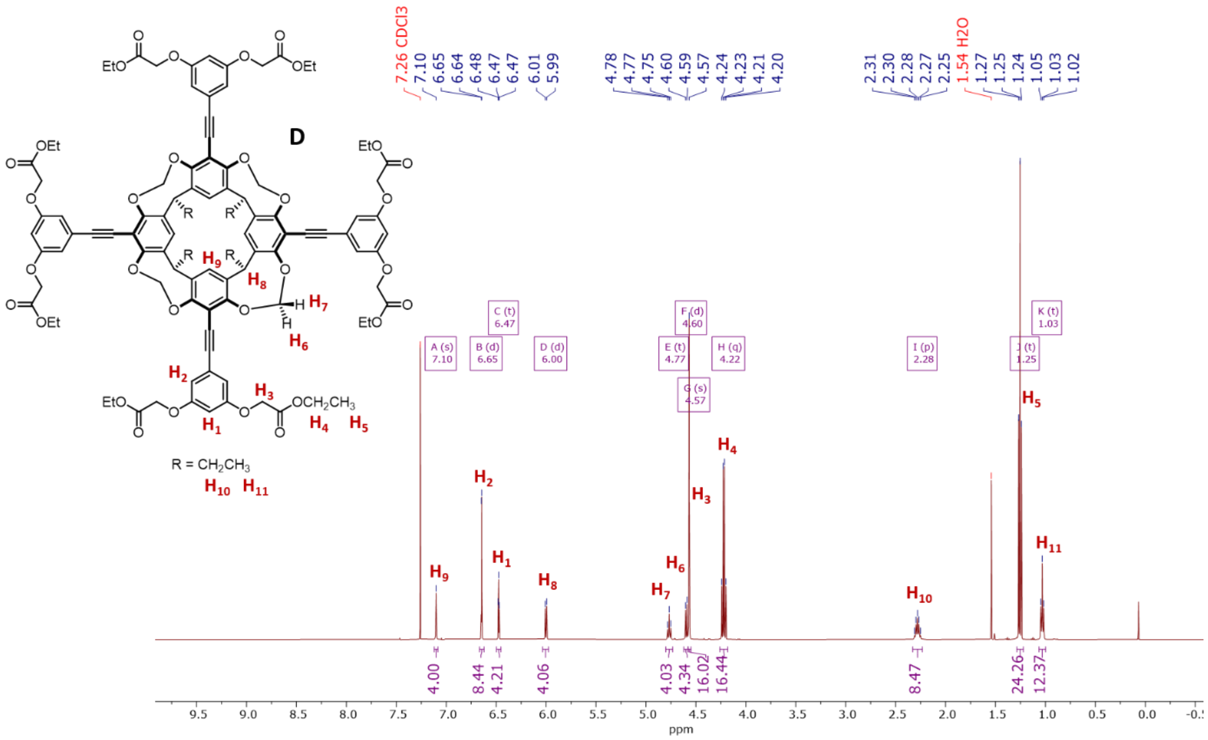

Figure S15: Full ^{1}H NMR (500 MHz, $CDCl_3$, 298 K) of compound **D**.

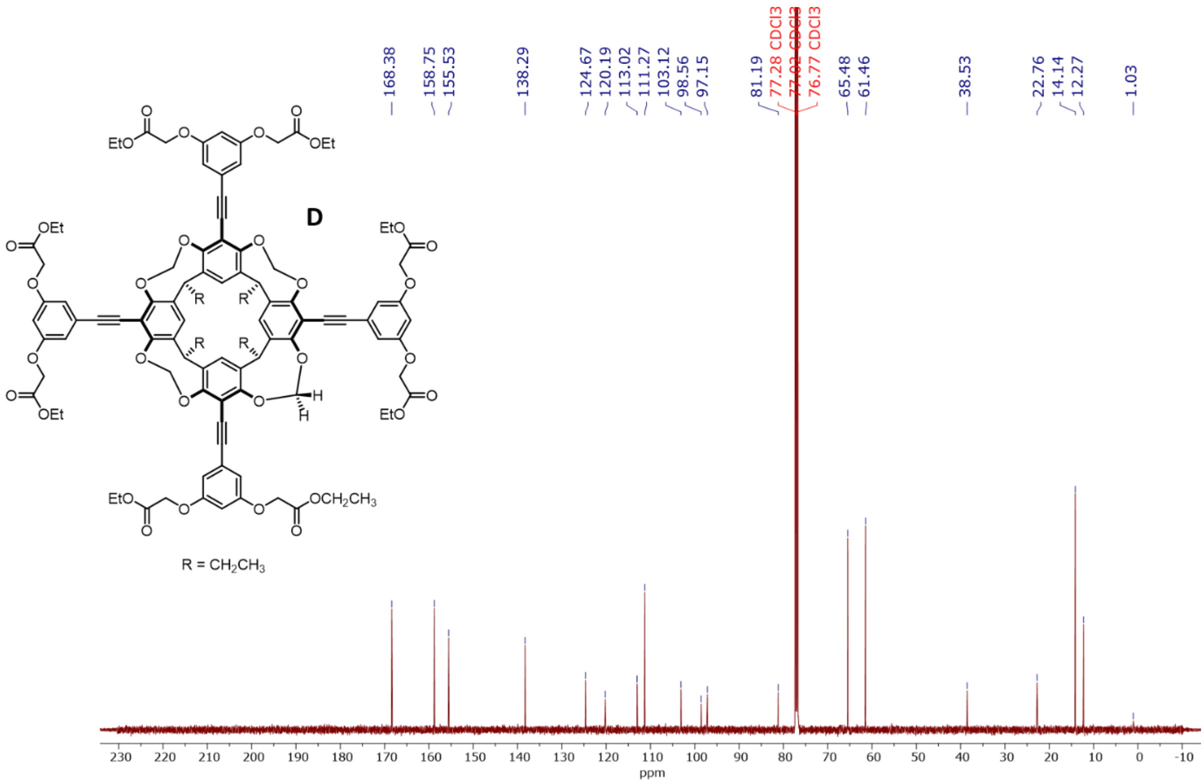

Figure S16: Full ^{13}C NMR {^{1}H} (126 MHz, $CDCl_3$, 298 K) of compound **D**.

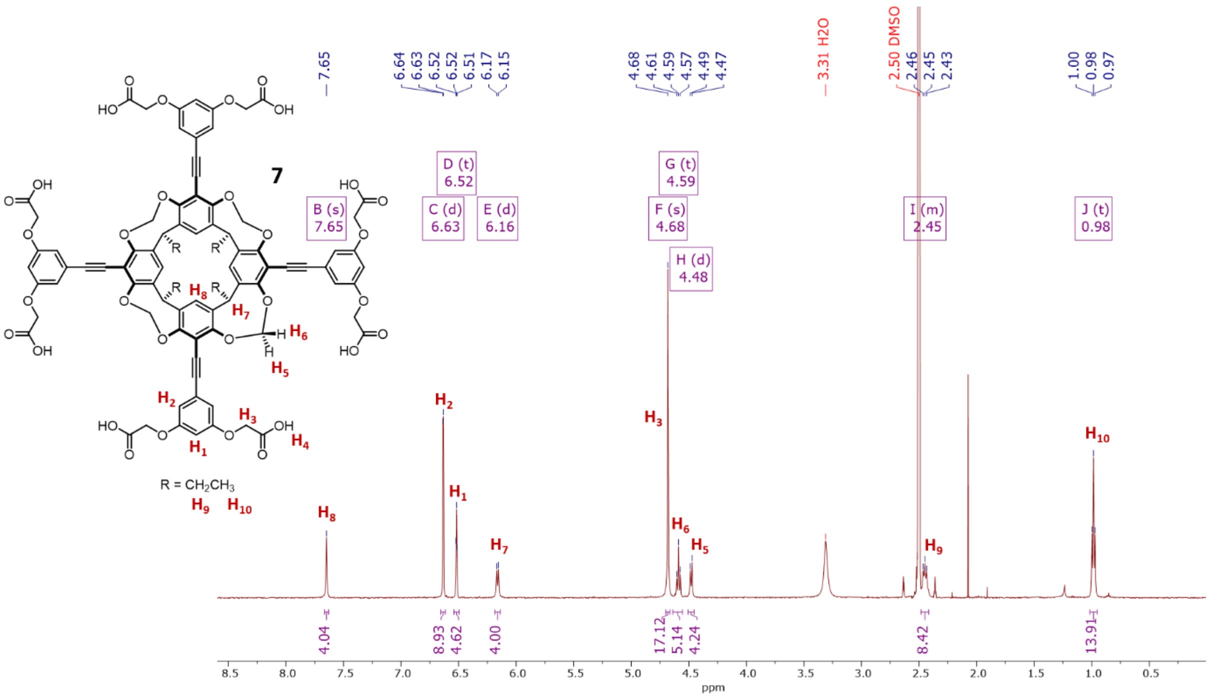

Figure S17: Full ^{1}H NMR (500 MHz, DMSO-d6, 298 K) of Cavitand **7**. Fast exchange compared to the NMR time scale is observed for carboxylic protons H_4.

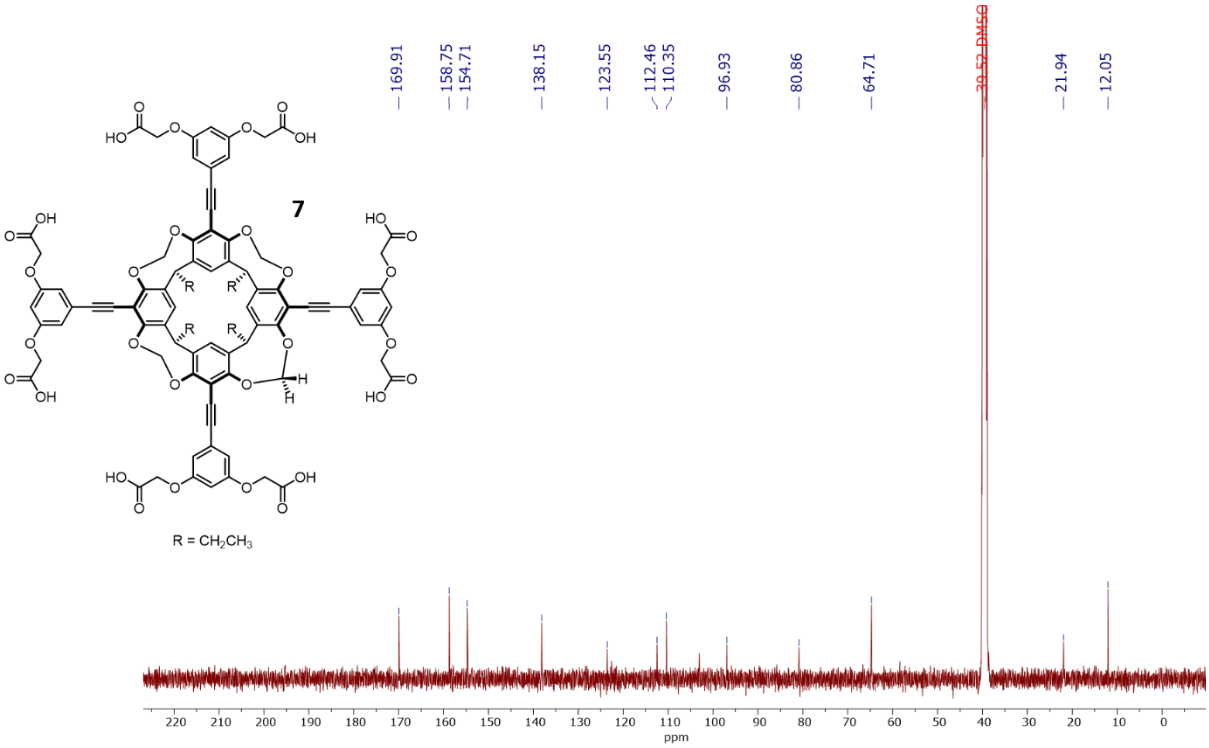

Figure S18: Full ^{13}C NMR {^{1}H} (126 MHz, DMSO-d6, 298 K) of Cavitand **7**.

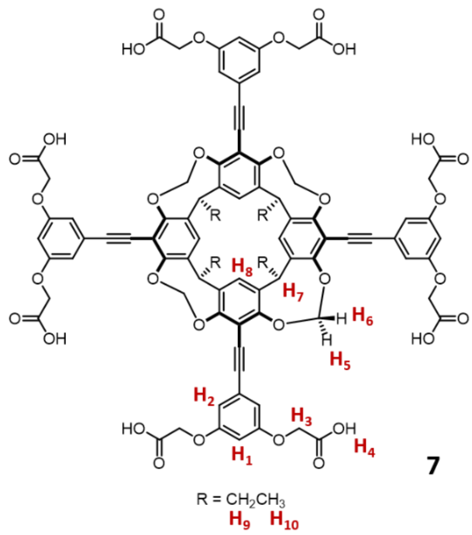

7

R = CH_2CH_3
H_9 H_{10}

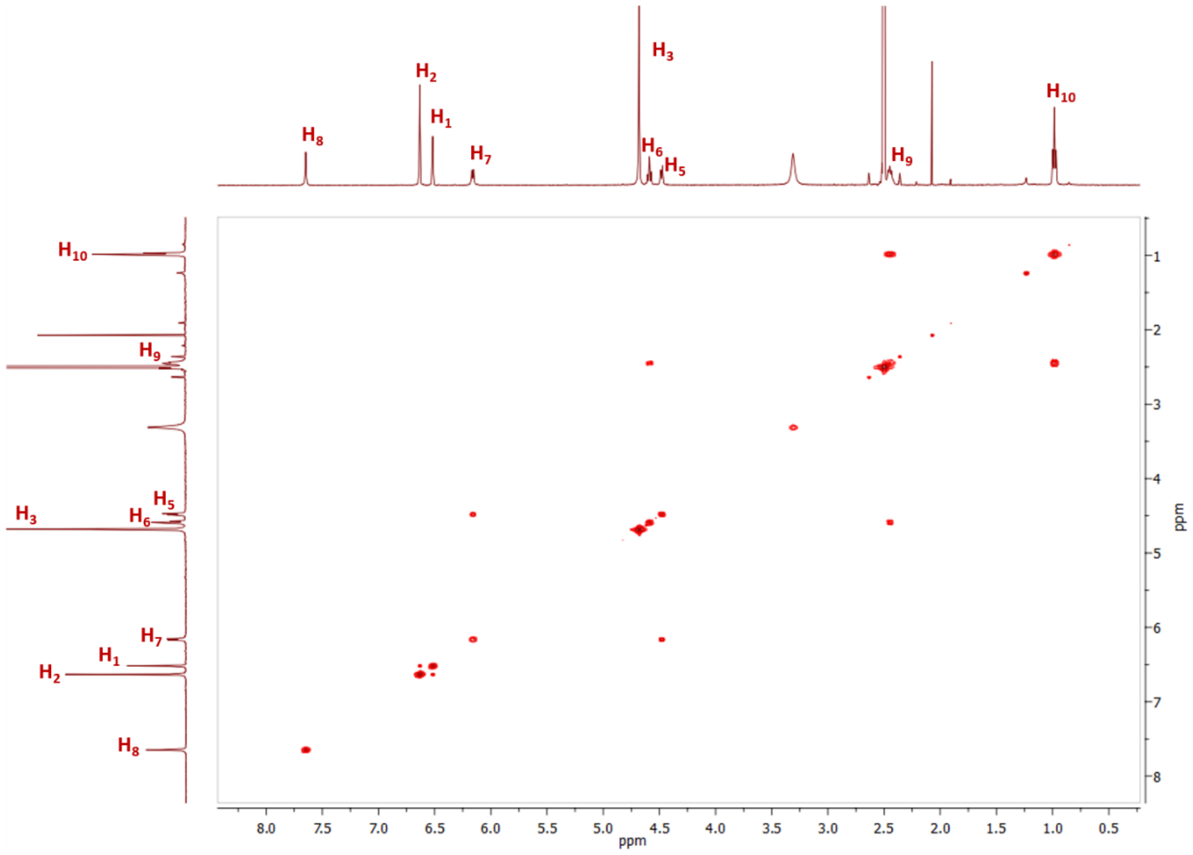

Figure S19: ^{1}H-^{1}H COSY (DMSO-d$_6$, 298 K) of Cavitand **7**.

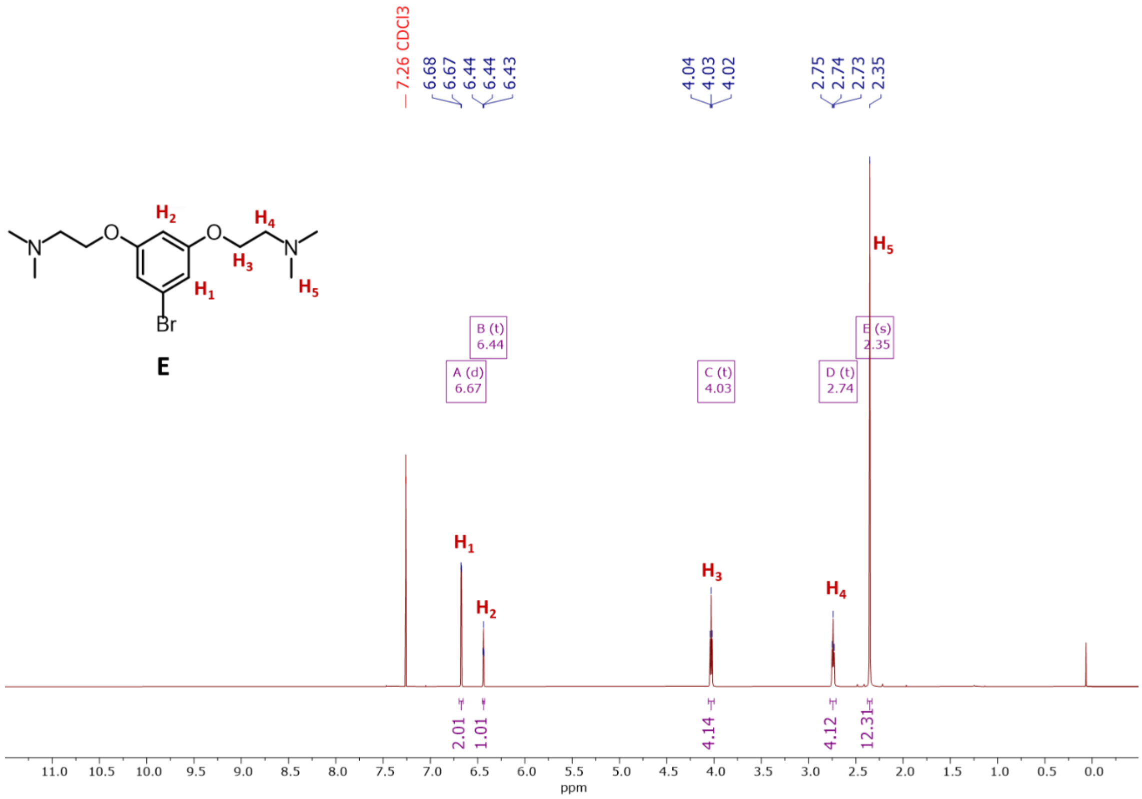

Figure S20: Full ^{1}H NMR (500 MHz, DMSO-d$_6$, 298 K) of compound **E**.

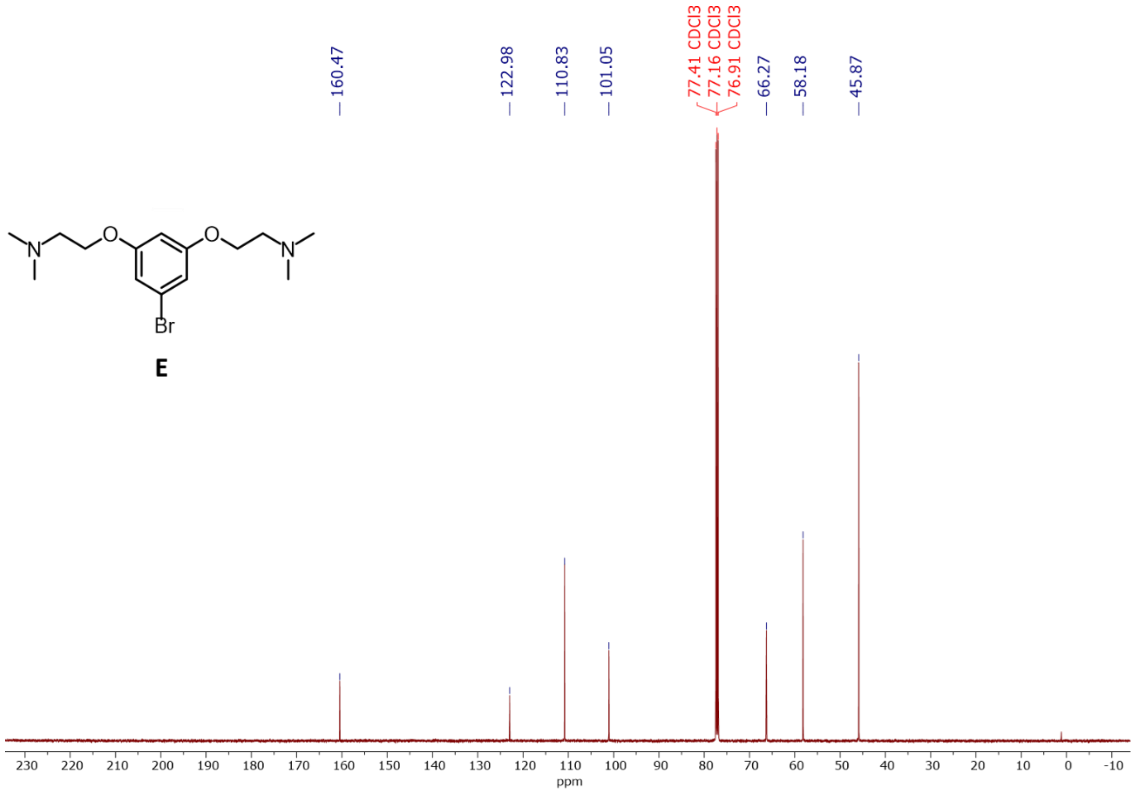

Figure S21: Full ^{13}C NMR {^{1}H} (126 MHz, $CDCl_3$, 298 K) of compound **E**.

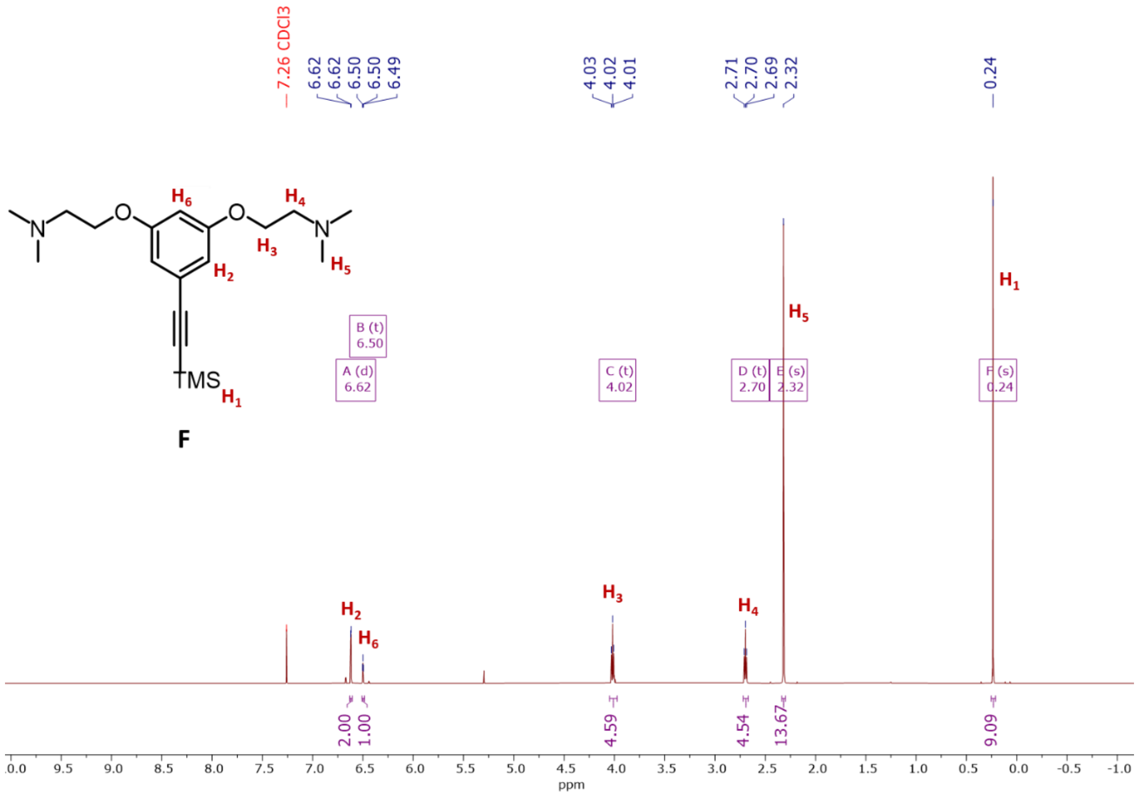

Figure S22: Full ^{1}H NMR (500 MHz, $CDCl_3$, 298 K) of compound **F**.

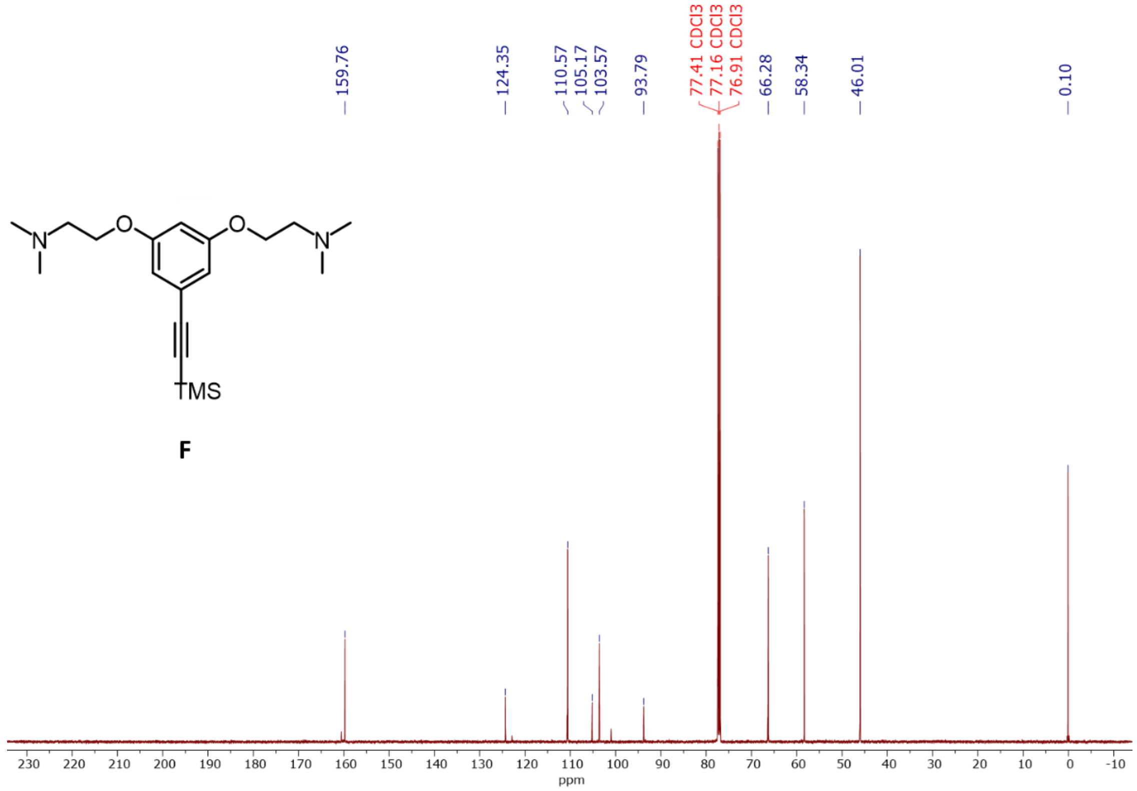

Figure S23: Full ^{13}C NMR {^{1}H} (126 MHz, $CDCl_3$, 298 K) of compound **F**.

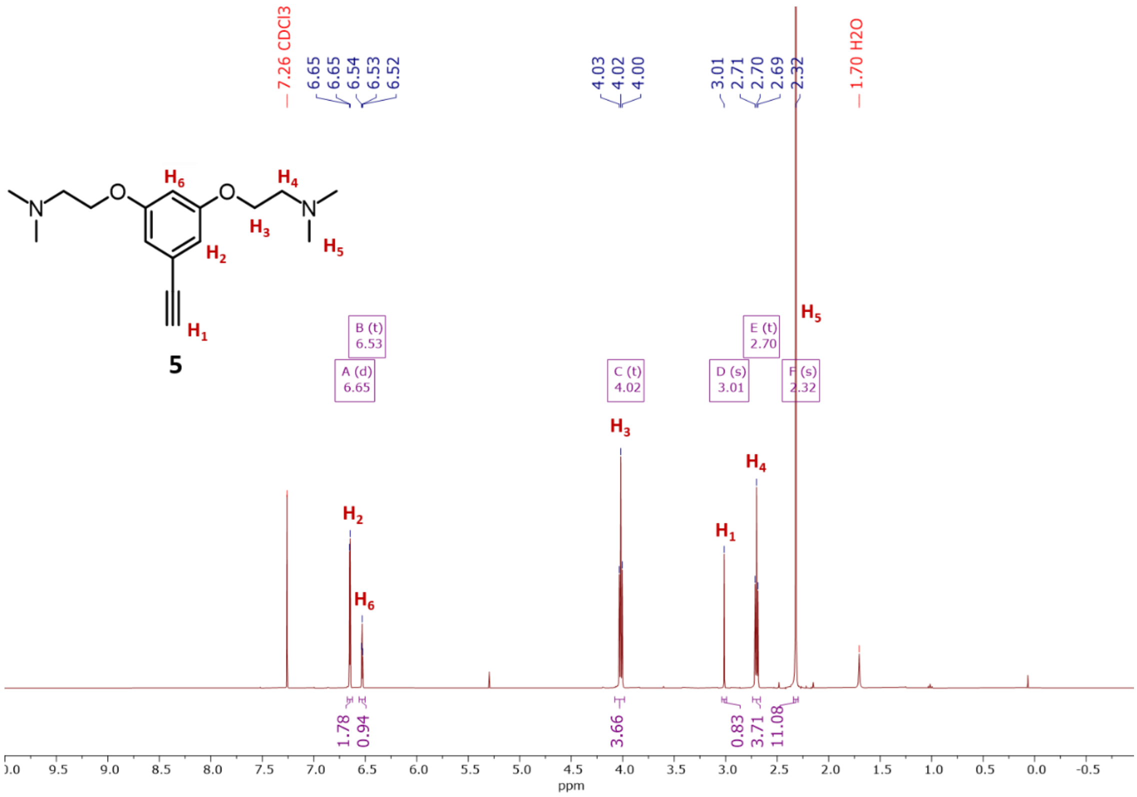

Figure S24: Full ^{1}H NMR (500 MHz, $CDCl_3$, 298 K) of alkyne **5**.

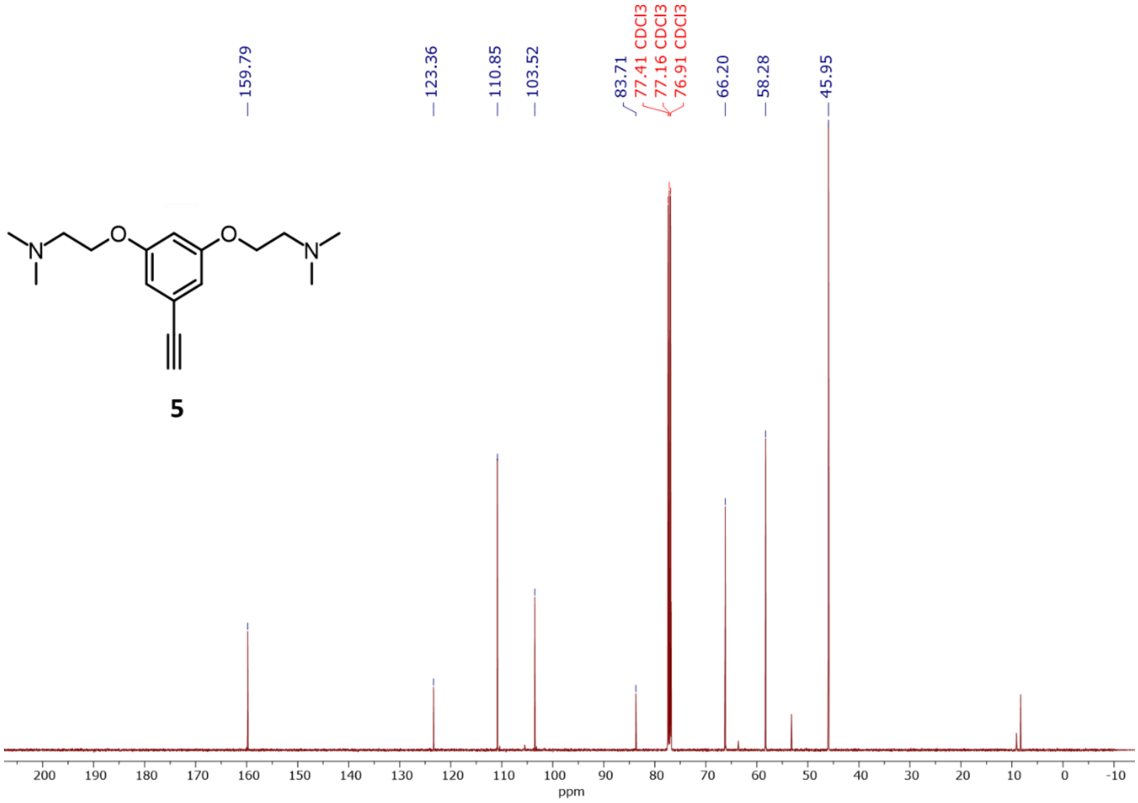

Figure S25: Full ^{13}C NMR {^{1}H} (126 MHz, $CDCl_3$, 298 K) of alkyne **5**.

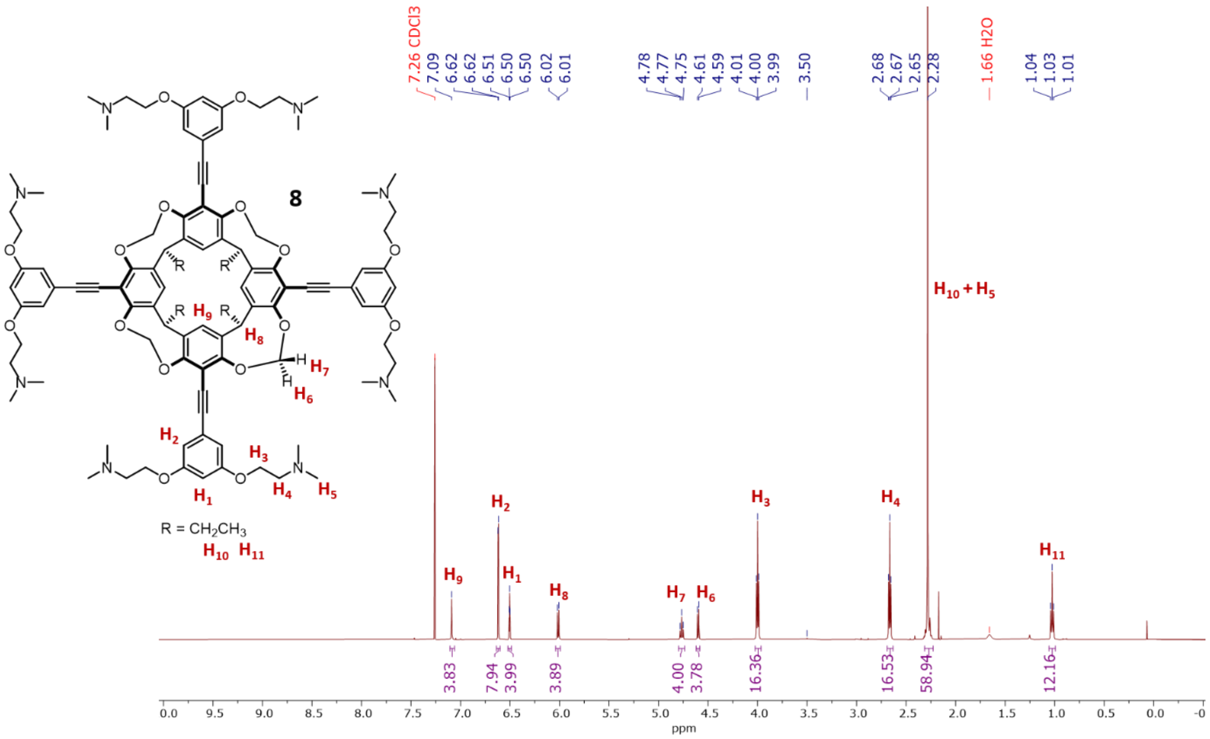

Figure S26: Full ^{1}H NMR (500 MHz, $CDCl_3$, 298 K) of Cavitand **8**.

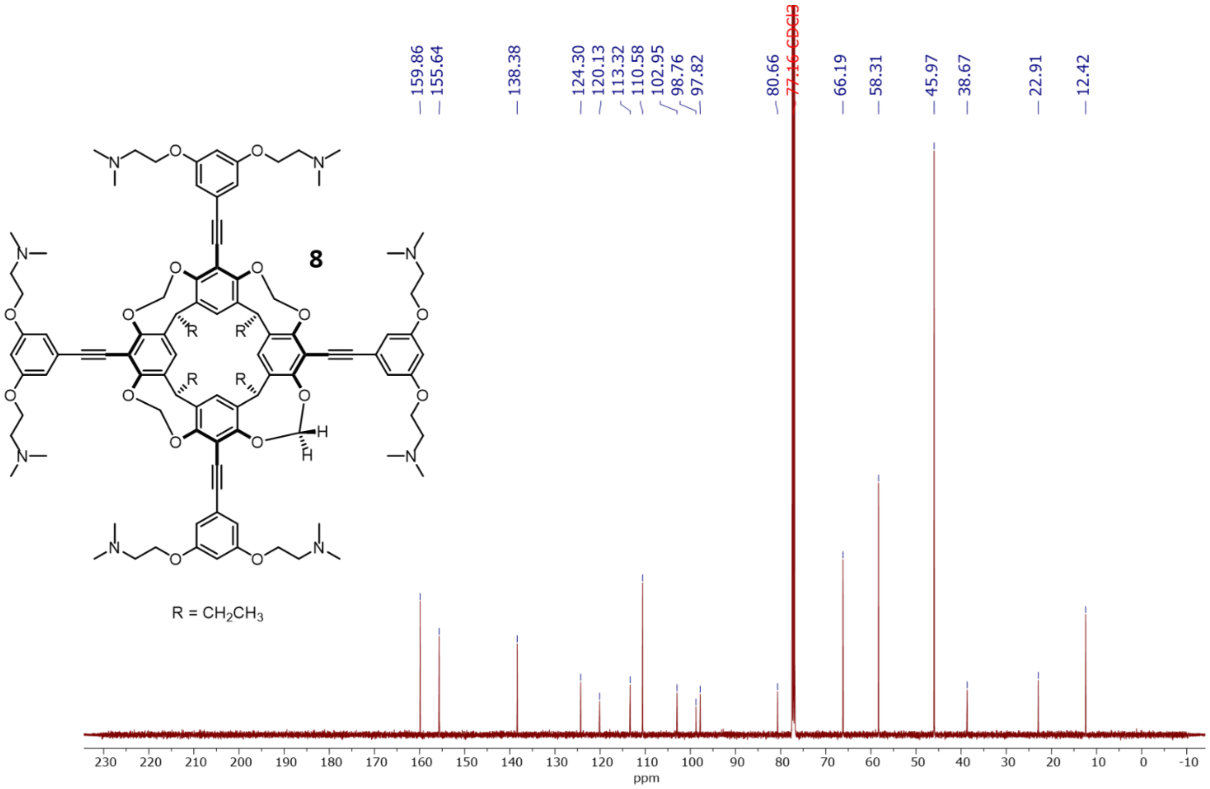

Figure S27: Full ^{13}C NMR {^{1}H} (126 MHz, $CDCl_3$, 298 K) of Cavitand **8**.

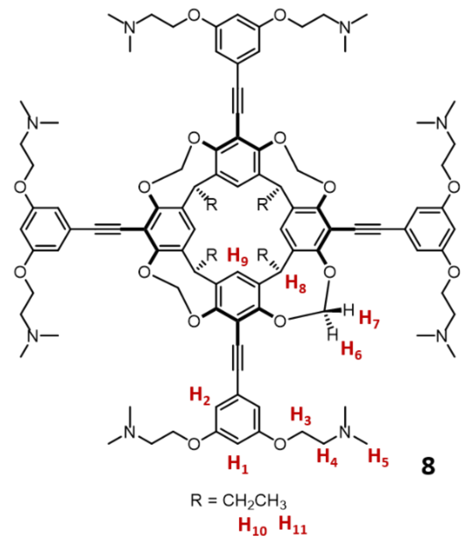

8

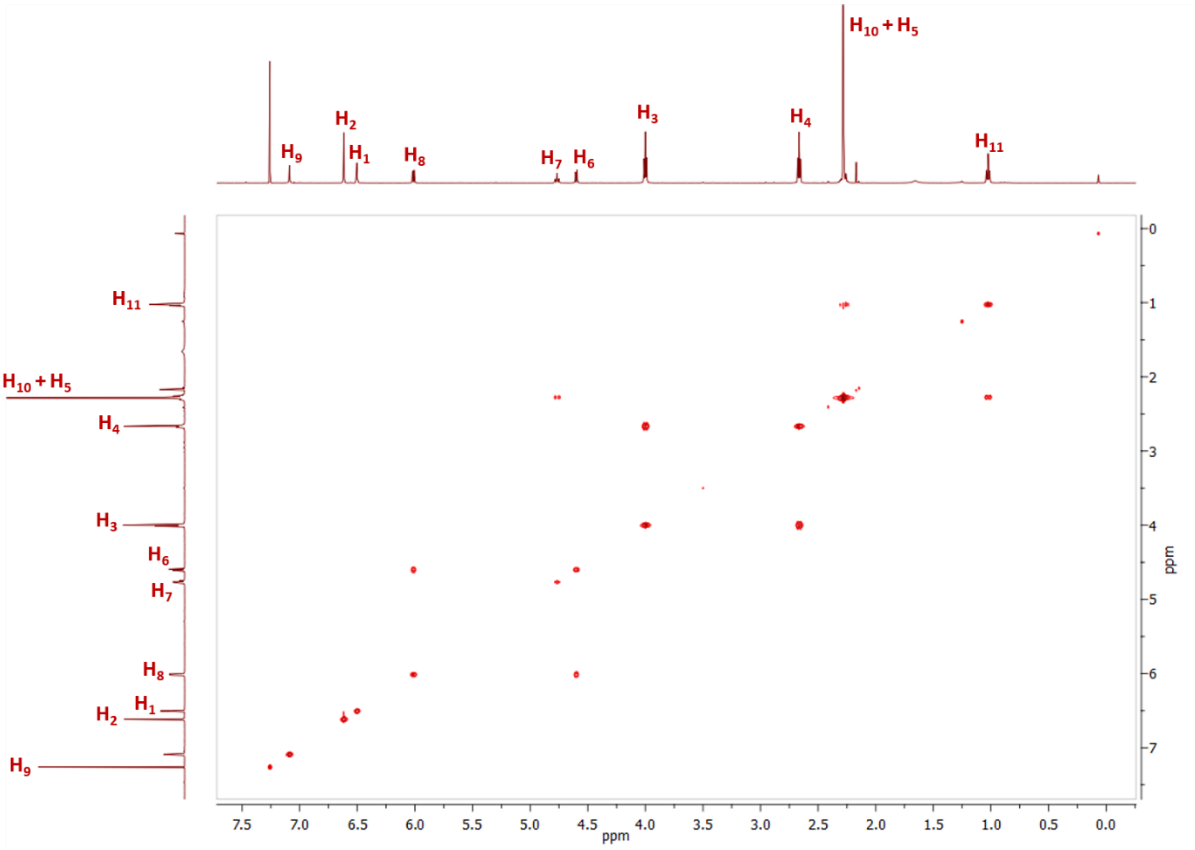

Figure S28: ^{1}H-1H COSY (CDCl₃, 298 K) of Cavitand **8**.

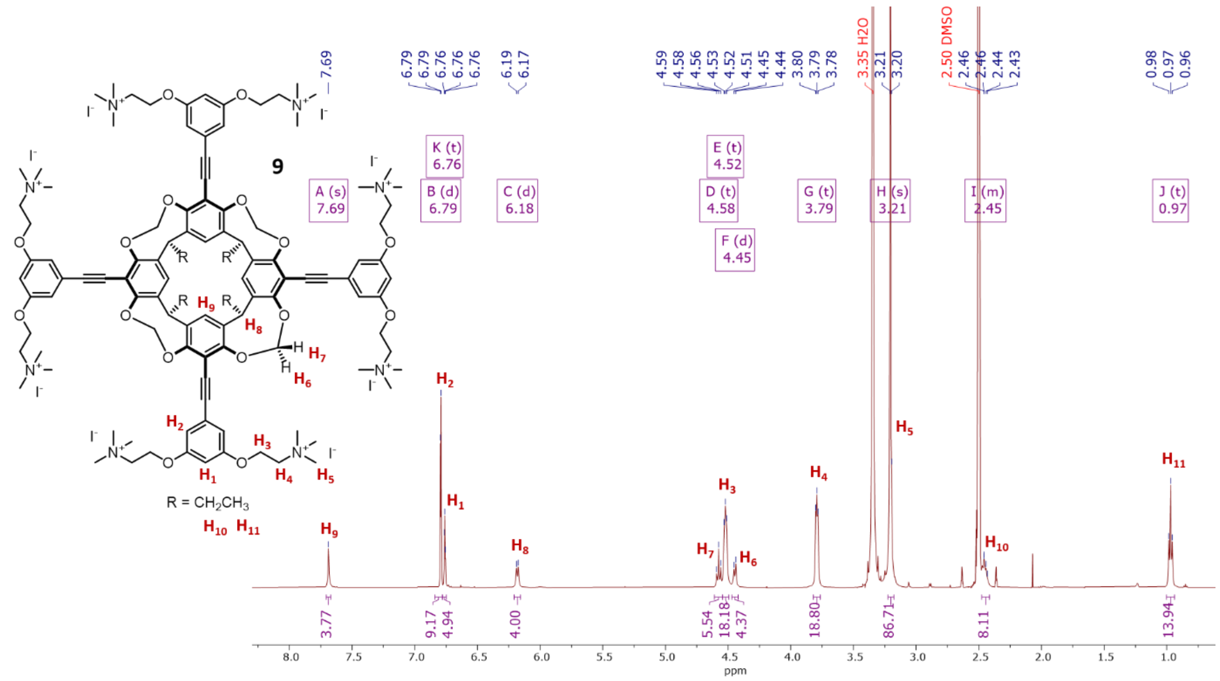

Figure S29: Full ^{1}H NMR (500 MHz, DMSO-d$_6$, 298 K) of Cavitand **9**.

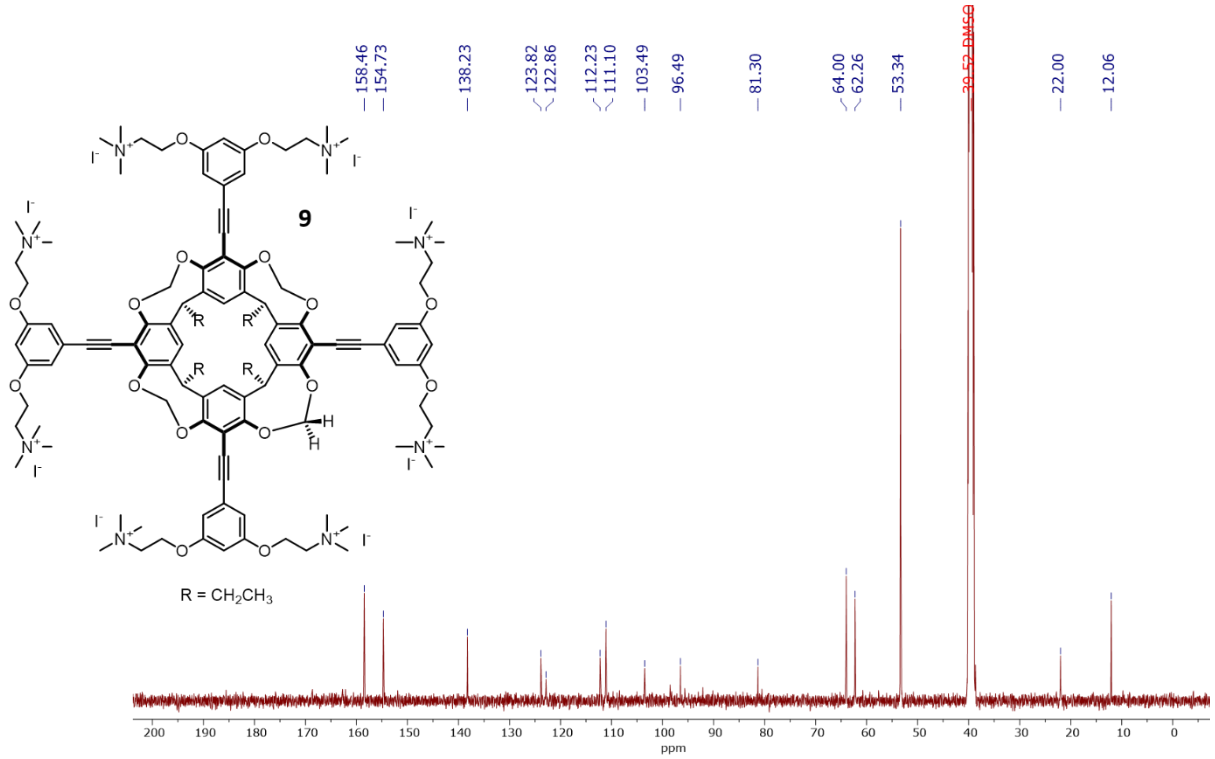

Figure S30: Full ^{13}C NMR {^{1}H} (126 MHz, DMSO-d$_6$, 298 K) of Cavitand **9**.

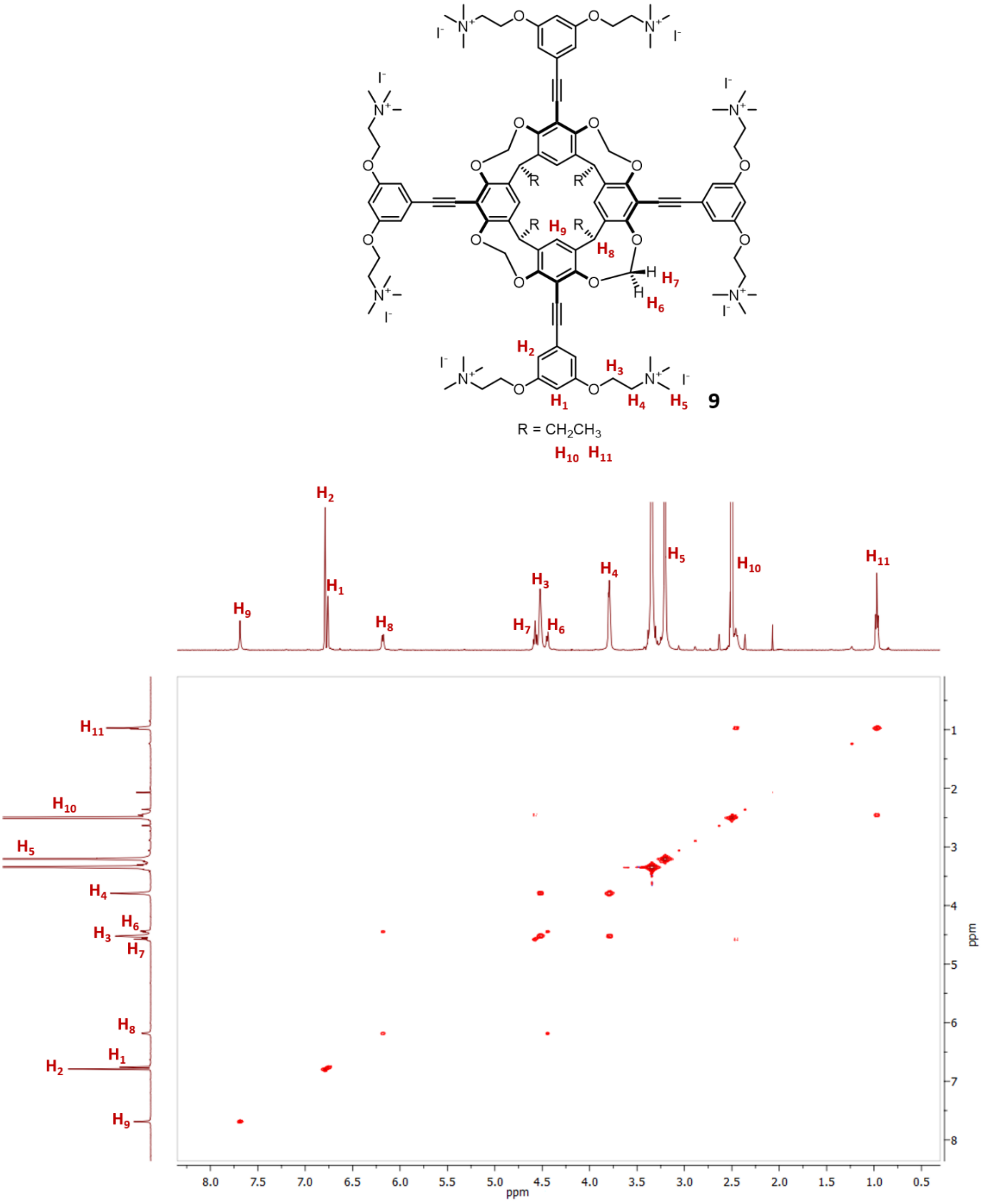

Figure S31: ^{1}H-^{1}H COSY (DMSO-d$_6$, 298 K) of Cavitand **9**.

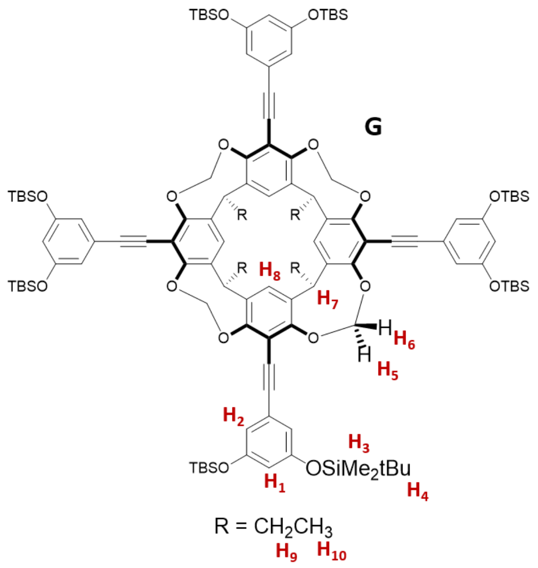

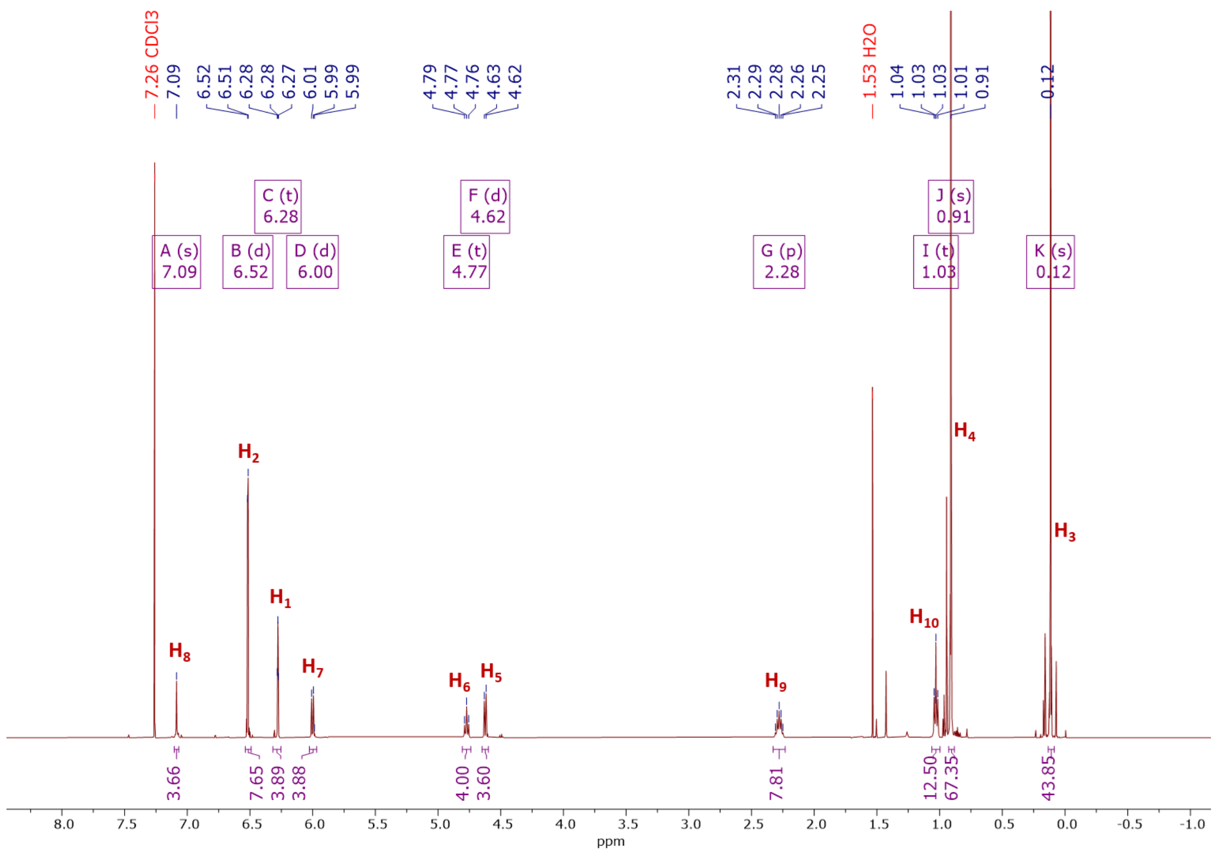

Figure S32: Full ^{1}H NMR (500 MHz, $CDCl_3$, 298 K) of compound **G**.

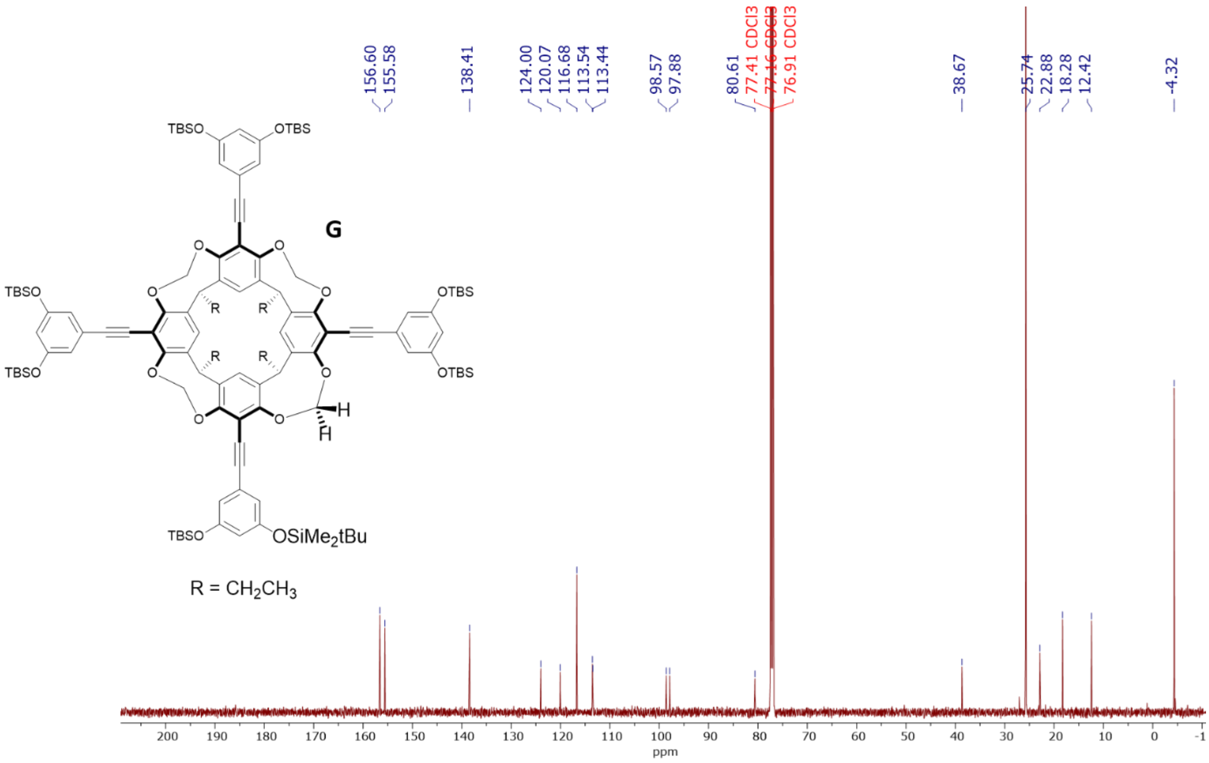

Figure S33: Full ^{13}C NMR {^{1}H} (126 MHz, $CDCl_3$, 298 K) of compound **G**.

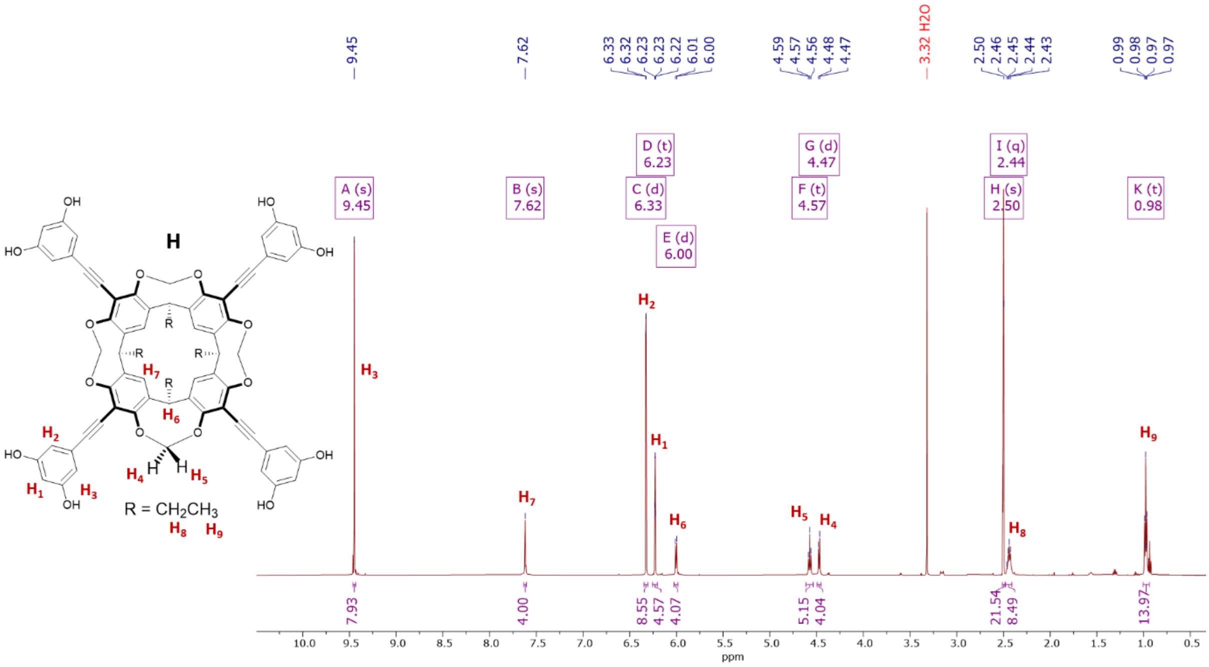

Figure S34: Full ^{1}H NMR (500 MHz, DMSO-d_6, 298 K) of compound **H**.

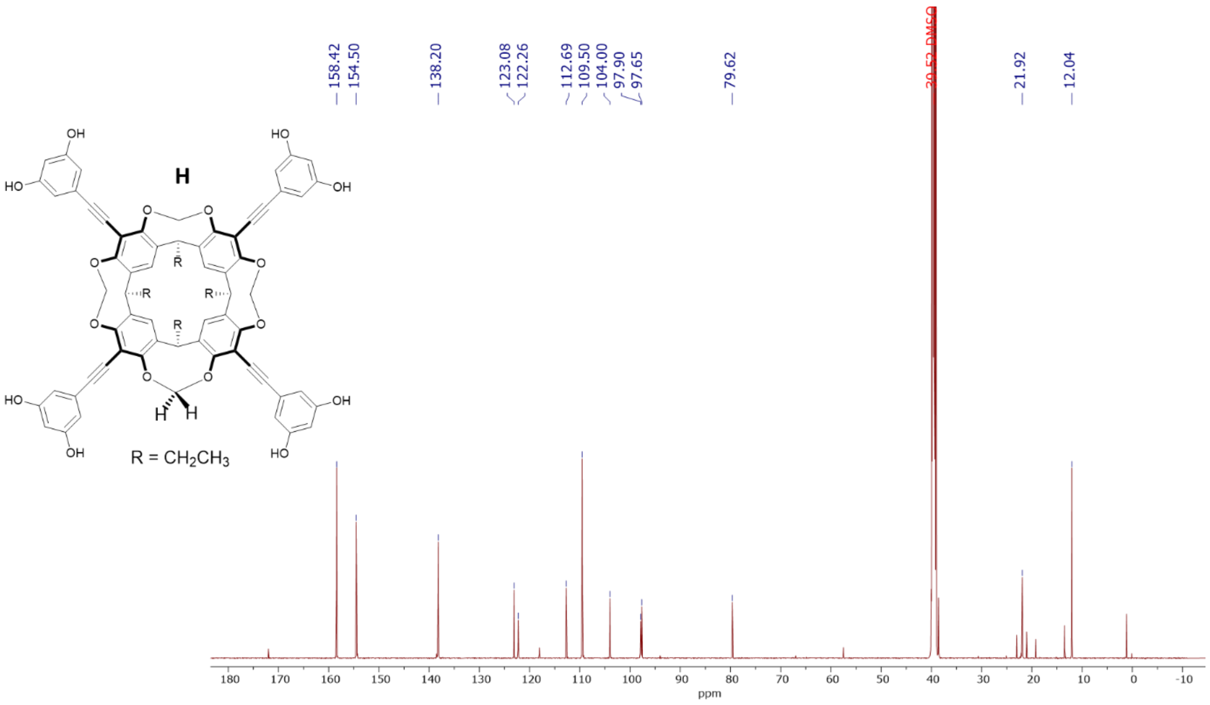

Figure S35: Full ^{13}C NMR {^{1}H} (126 MHz, DMSO-d$_6$, 298 K) of compound **H**.

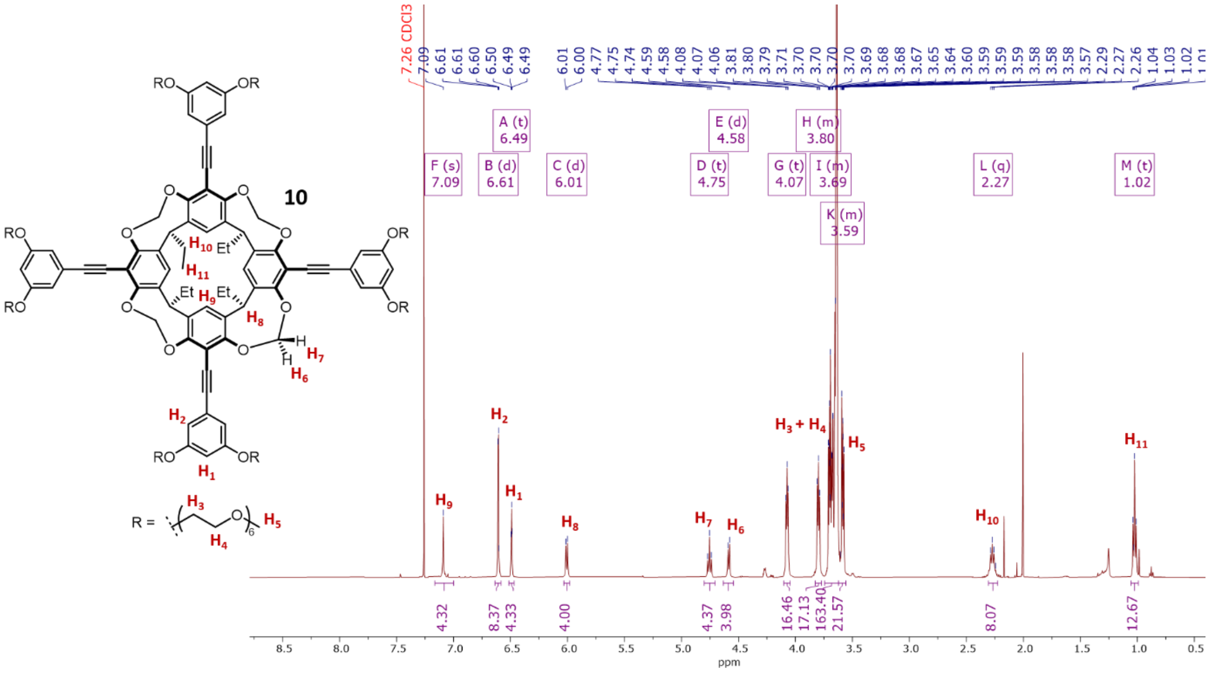

Figure S36: Full ^{1}H NMR (500 MHz, $CDCl_3$, 298 K) of Cavitand **10**.

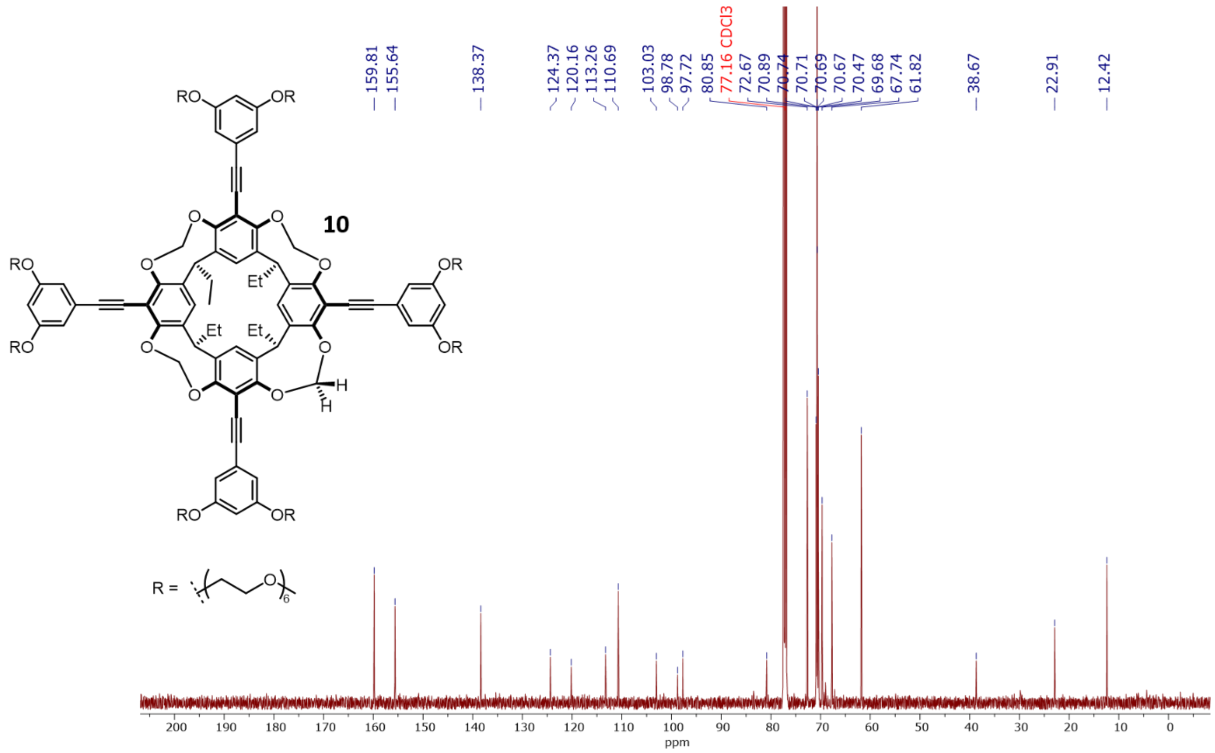

Figure S37: Full ^{13}C NMR $\{^1H\}$ (126 MHz, $CDCl_3$, 298 K) of Cavitand **10**.

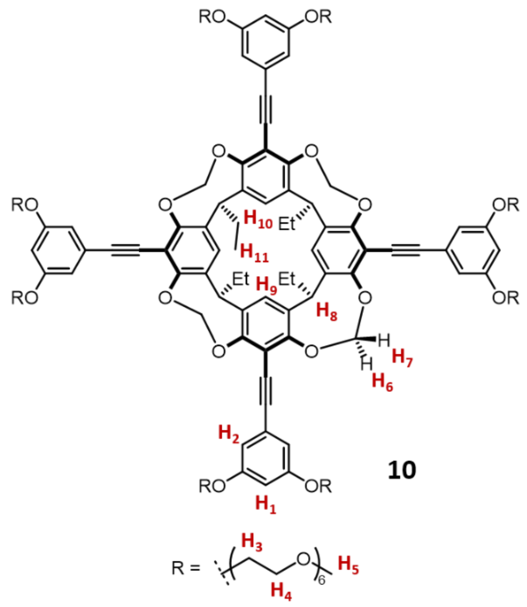

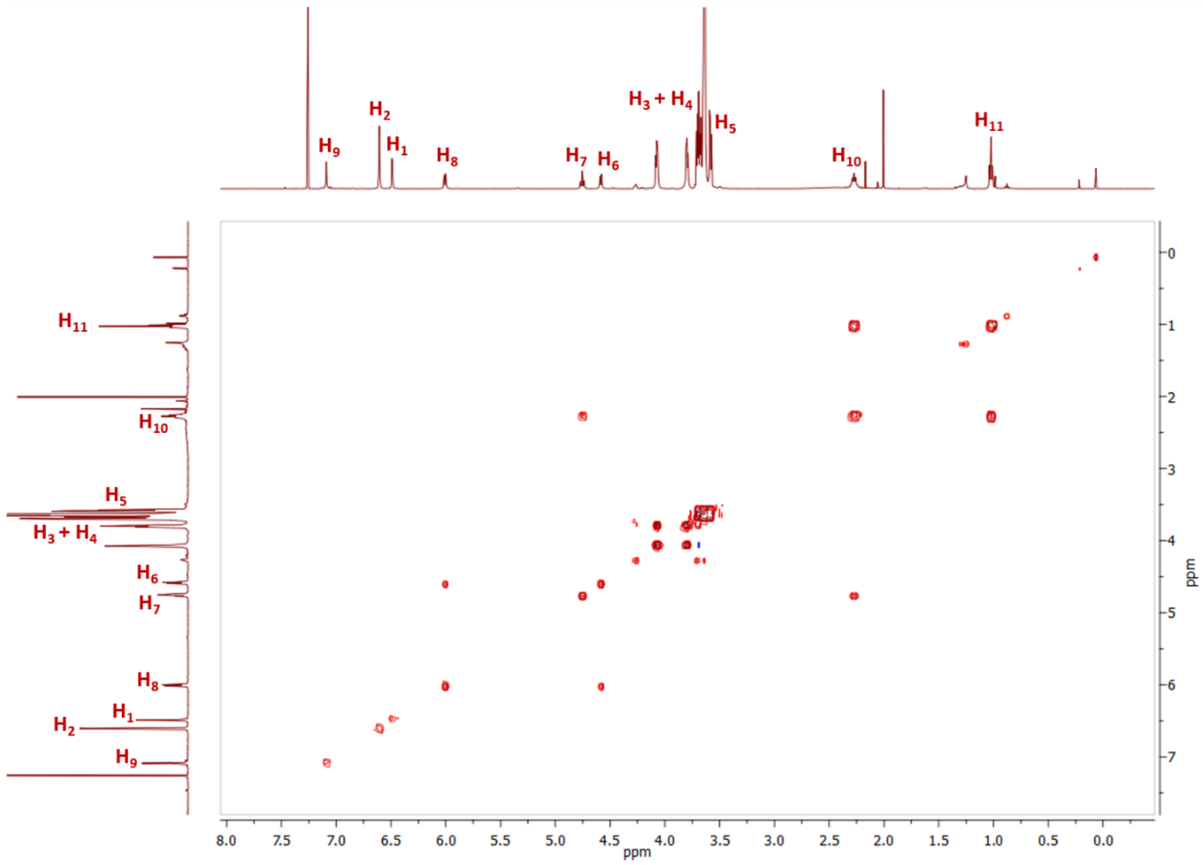

Figure S38: ^{1}H-^{1}H COSY ($CDCl_3$, 298 K) of Cavitand **10**.

4.2 MS Spectra

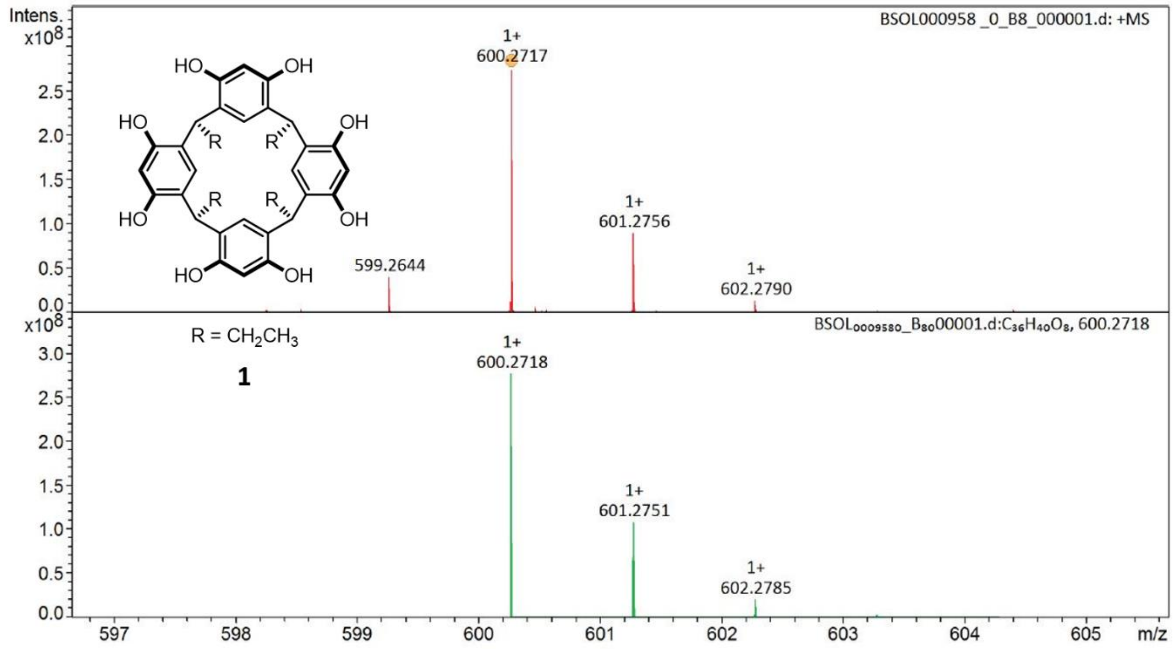

Figure S39: HR-ESIMS spectrum (+MS) of Compound **1** (Exact mass for $[M]^+$: 600.2718).

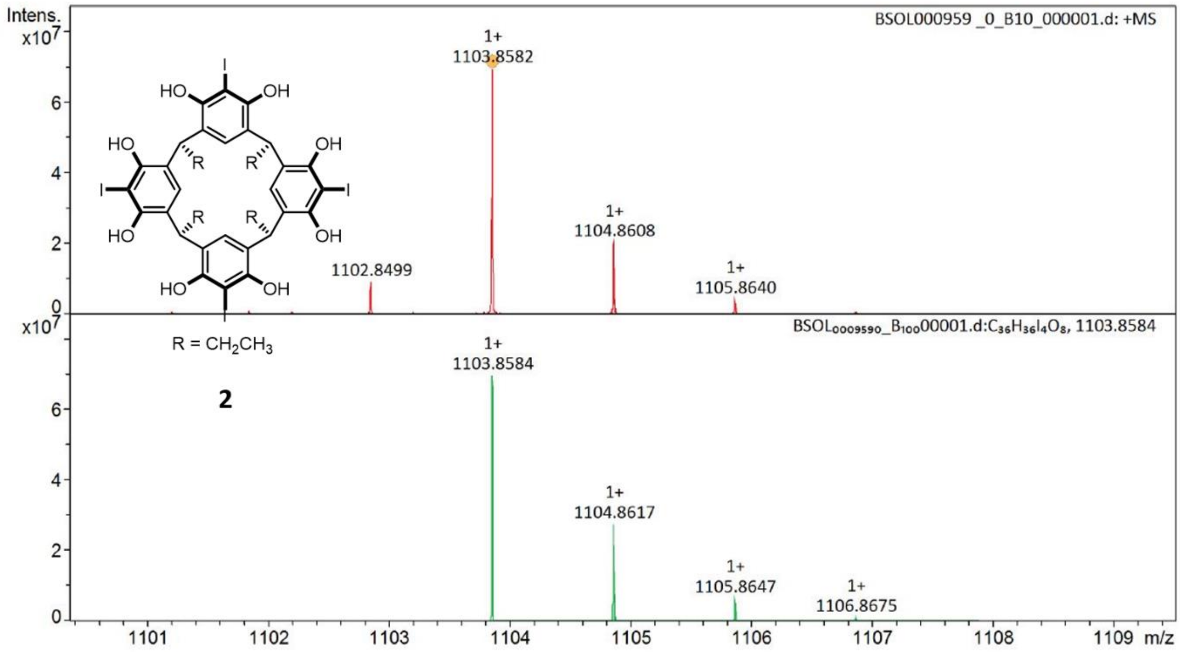

Figure S40: HR-ESIMS spectrum (+MS) of Compound **2** (Exact mass for $[M]^+$: 1103.8584).

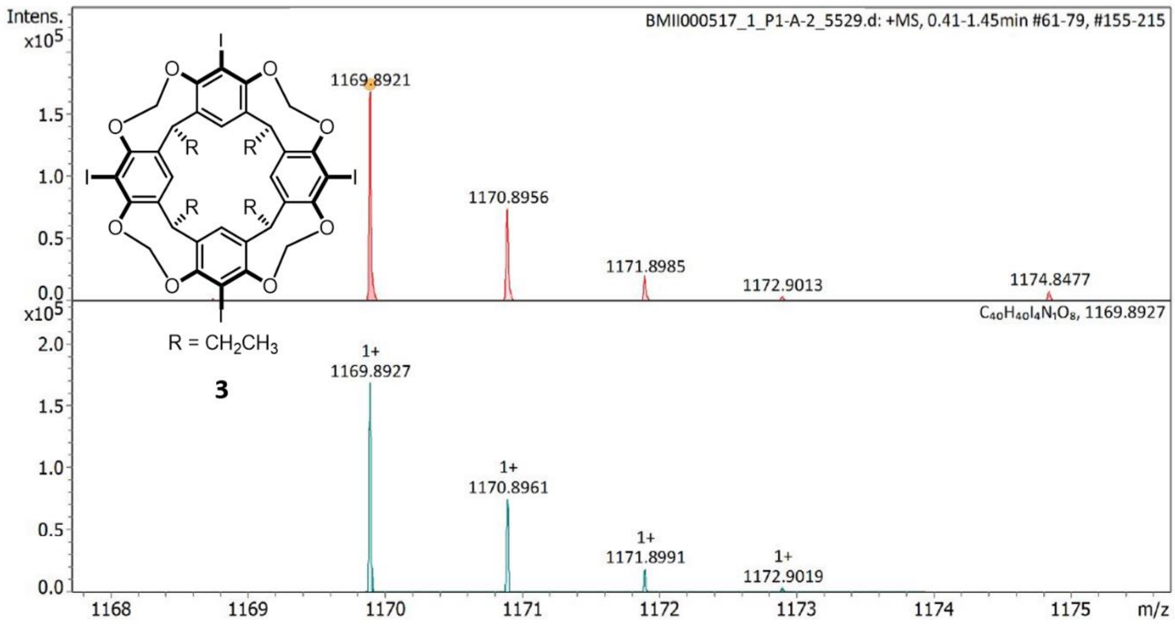

Figure S41: HR-ESIMS spectrum (+MS) of Compound **3** (KIC) (Exact mass for $[M+NH_4]^+$: 1169.8927).

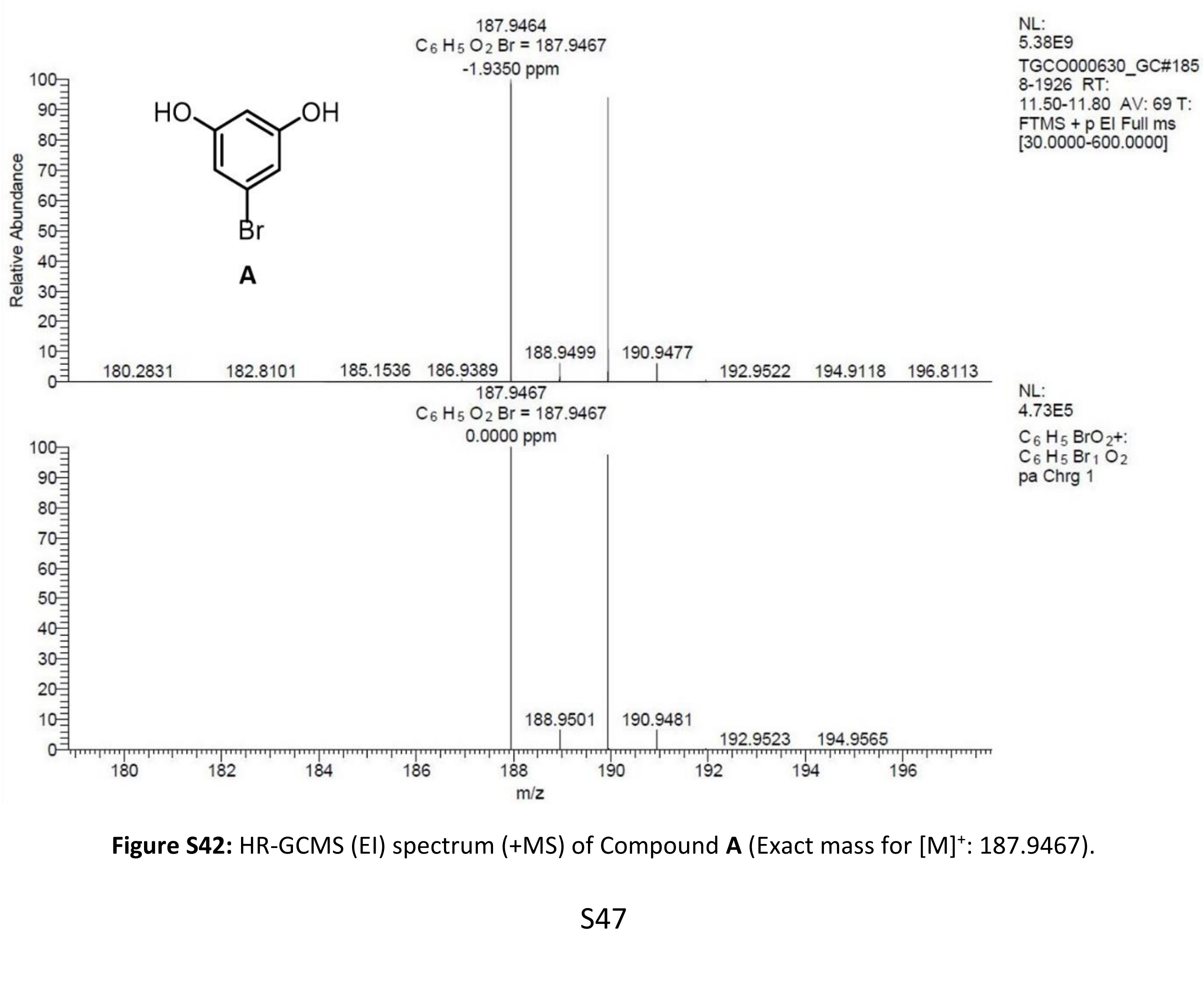

Figure S42: HR-GCMS (EI) spectrum (+MS) of Compound **A** (Exact mass for $[M]^+$: 187.9467).

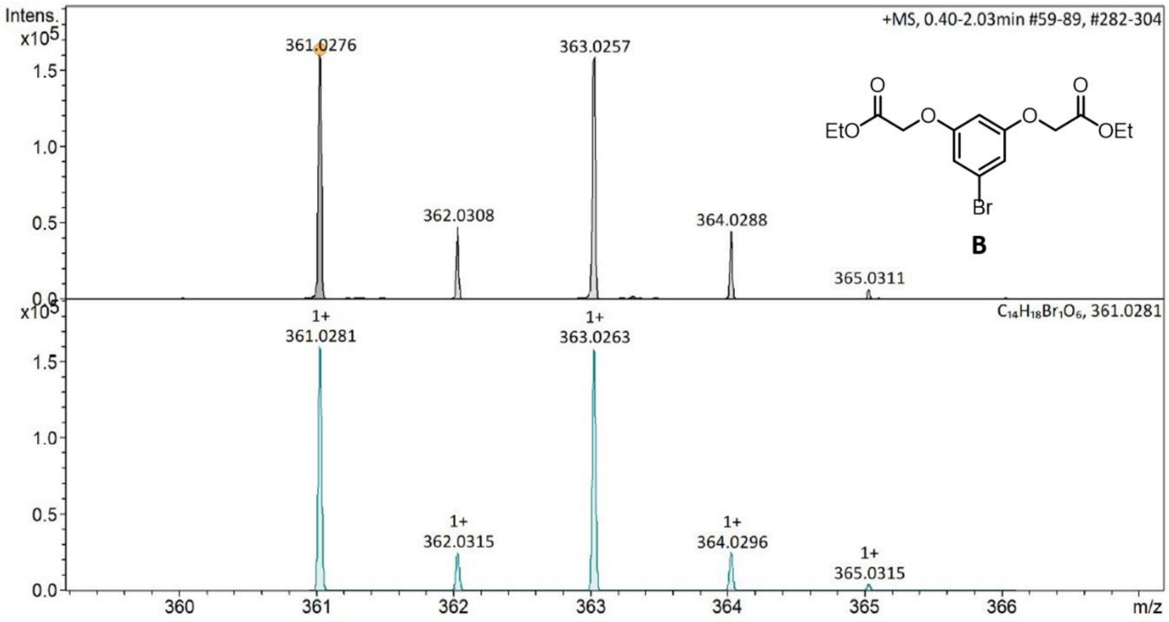

Figure S43: HR-ESIMS spectrum (+MS) of Compound **B** (Exact mass for $[M]^+$: 361.0281).

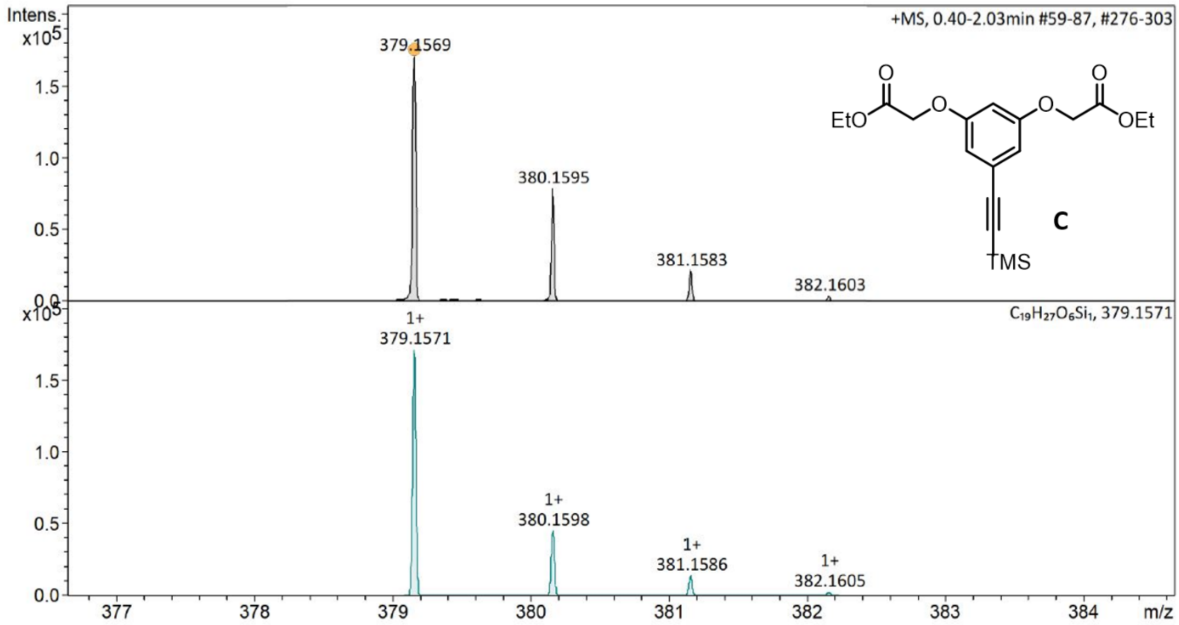

Figure S44: HR-ESIMS spectrum (+MS) of Compound **C** (Exact mass for $[M+H]^+$: 379.1571).

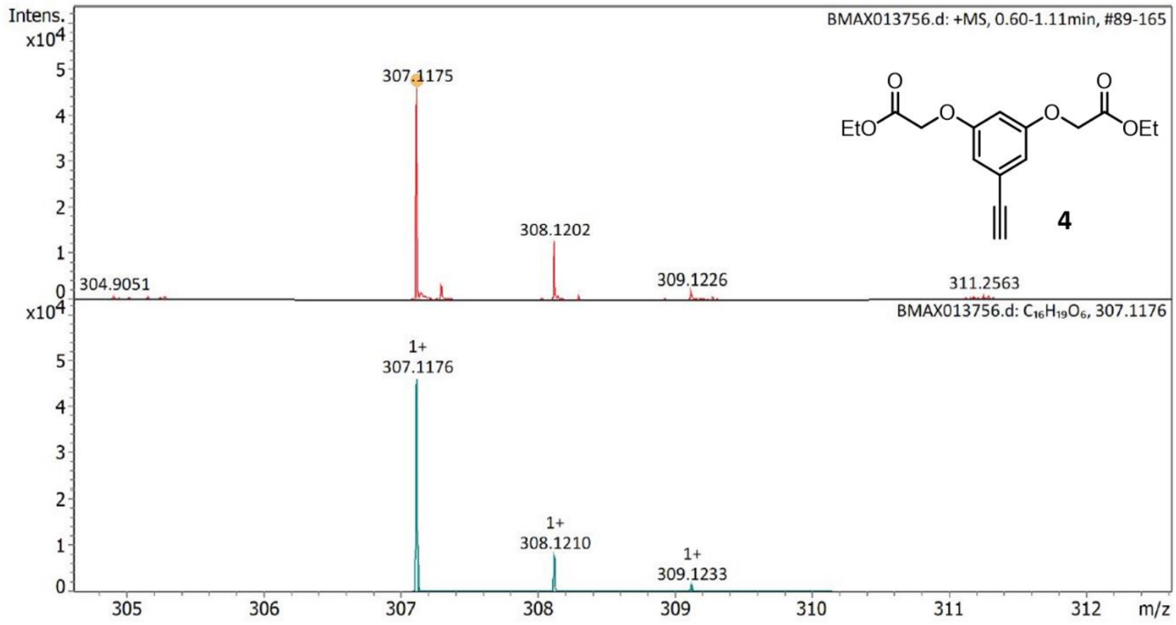

Figure S45: HR-ESIMS spectrum (+MS) of Compound **4** (Exact mass for $[M+H]^+$: 307.1176).

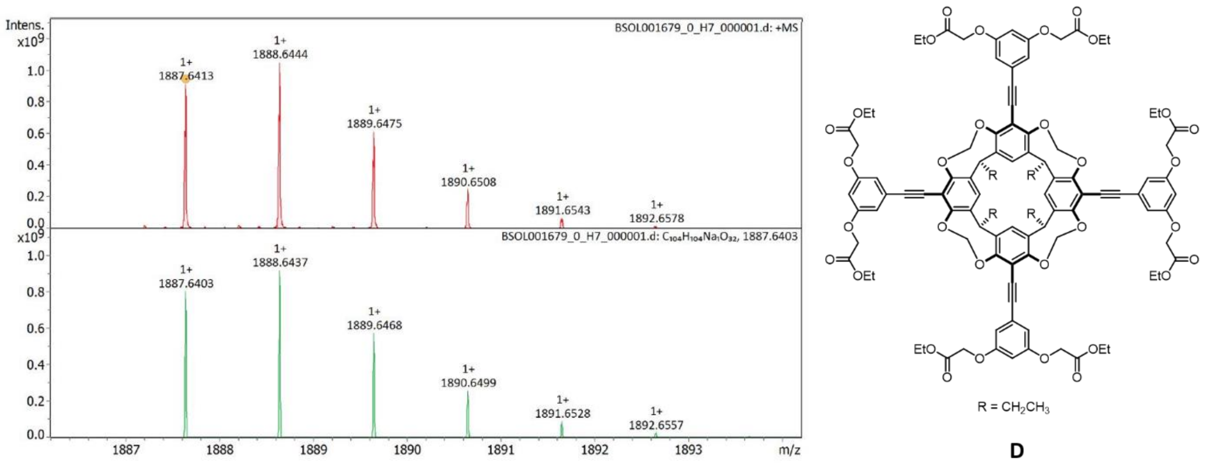

Figure S46: HR-ESIMS spectrum (+MS) of Compound **D** (Exact mass for $[M+Na]^+$: 1887.6403).

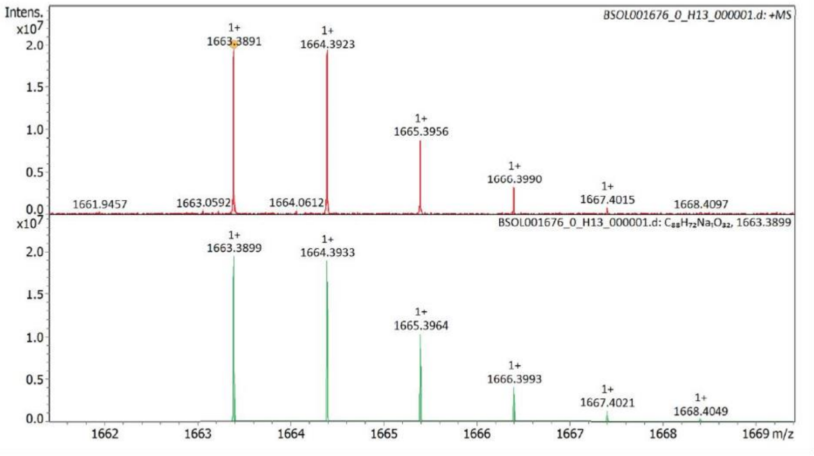

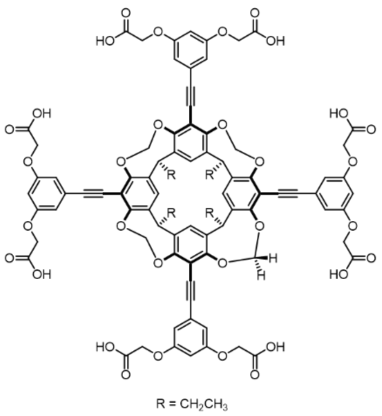

7

Figure S47: HR-ESIMS spectrum (+MS) of Cavitand **7** (Exact mass for $[M+Na]^+$: 1663.3899).

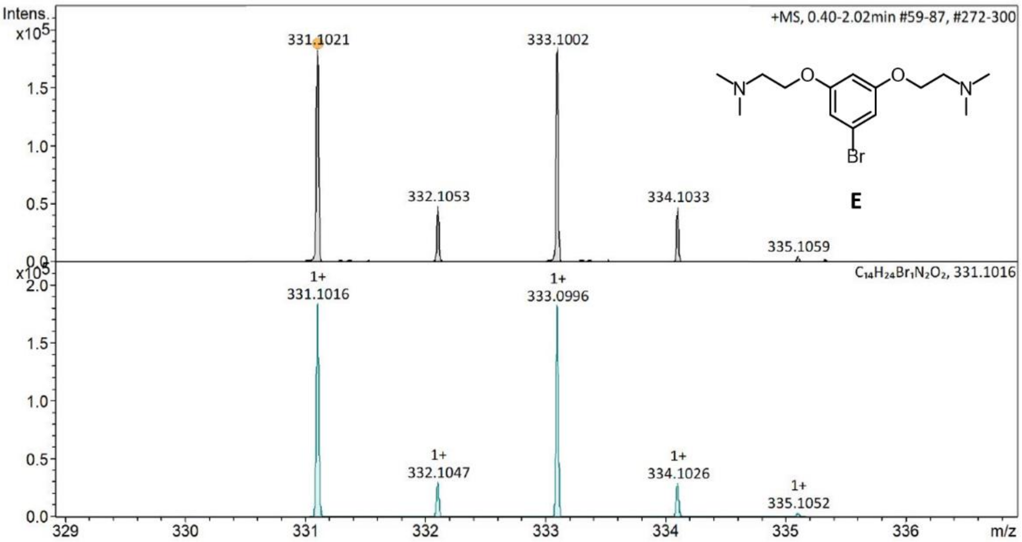

Figure S48: HR-ESIMS spectrum (+MS) of Compound **E** (Exact mass for $[M+H]^+$: 331.1016).

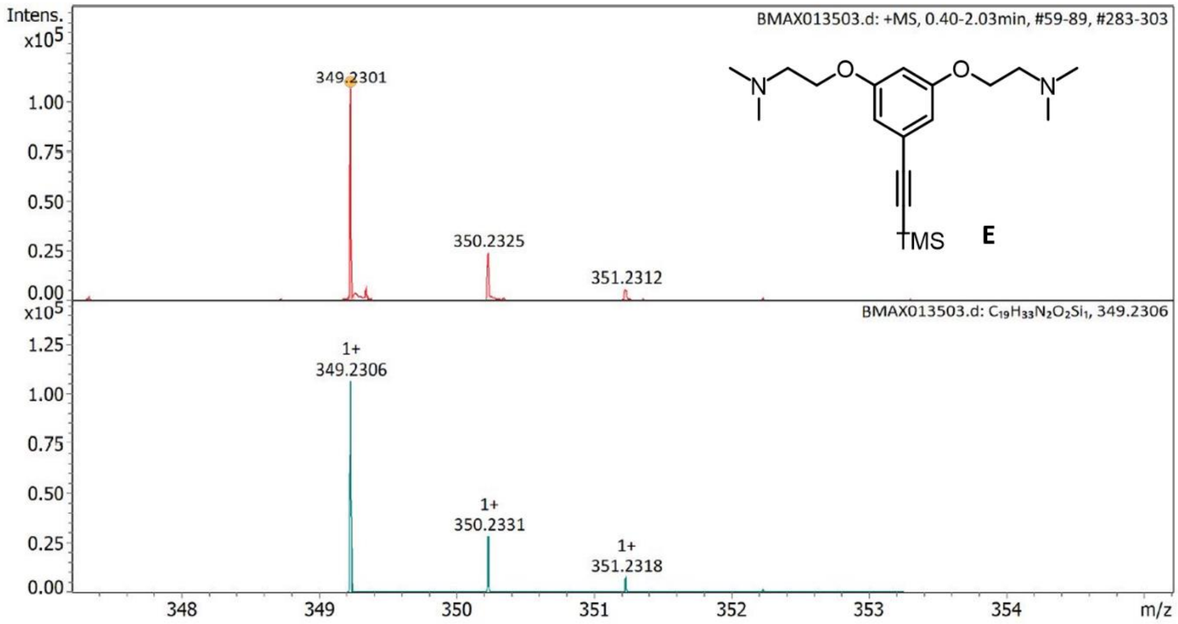

Figure S49: HR-ESIMS spectrum (+MS) of Compound **F** (Exact mass for $[M+H]^+$: 349.2306).

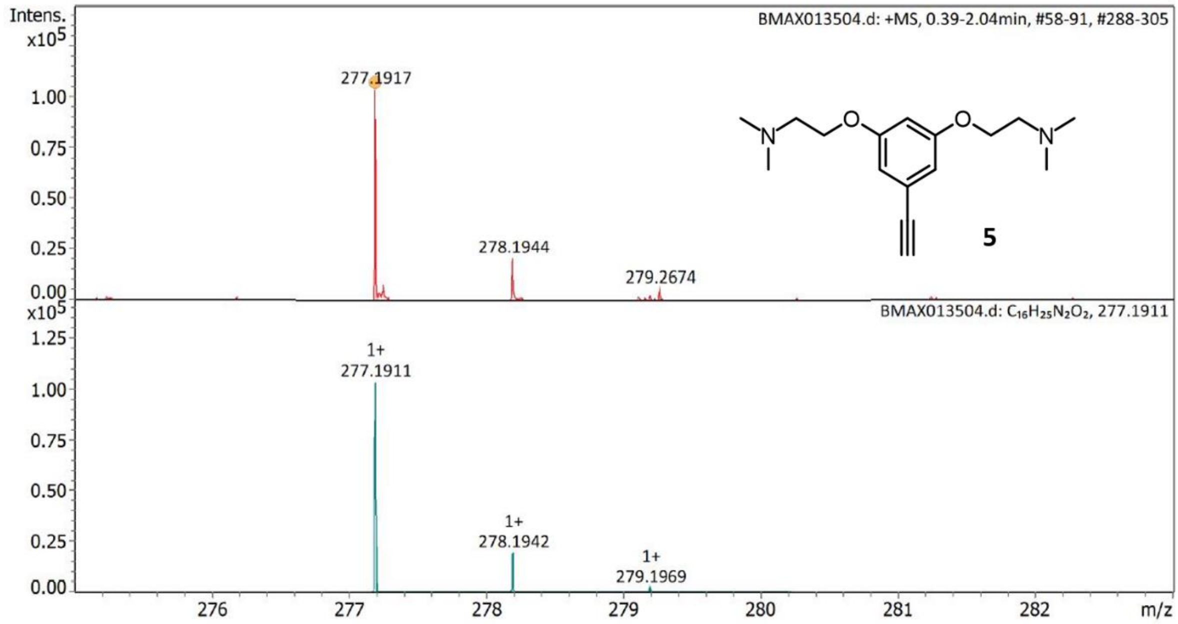

Figure S50: HR-ESIMS spectrum (+MS) of Compound **5** (Exact mass for $[M+H]^+$: 277.1911).

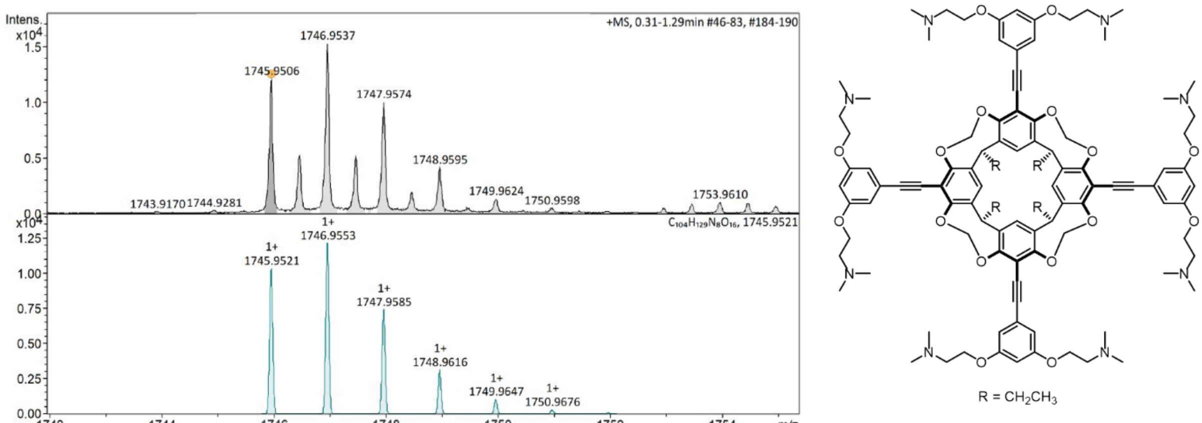

8

Figure S51: HR-MS spectrum (+MS) of Cavitand **8** (Exact mass for $[M+H]^{+}$: 1745.9521).

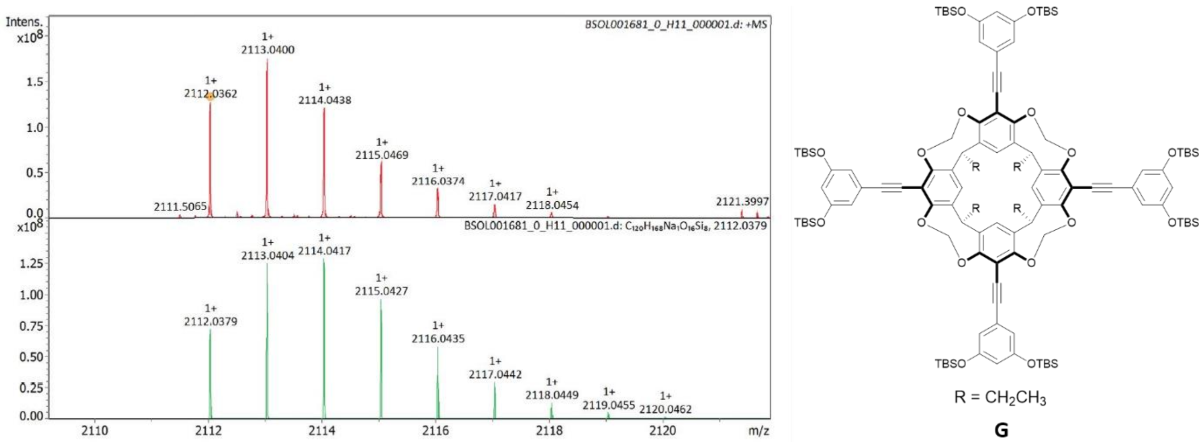

G

Figure S52: HR-ESIMS spectrum (+MS) of Compound **G** (Exact mass for $[M+Na]^{+}$: 2112.0379).

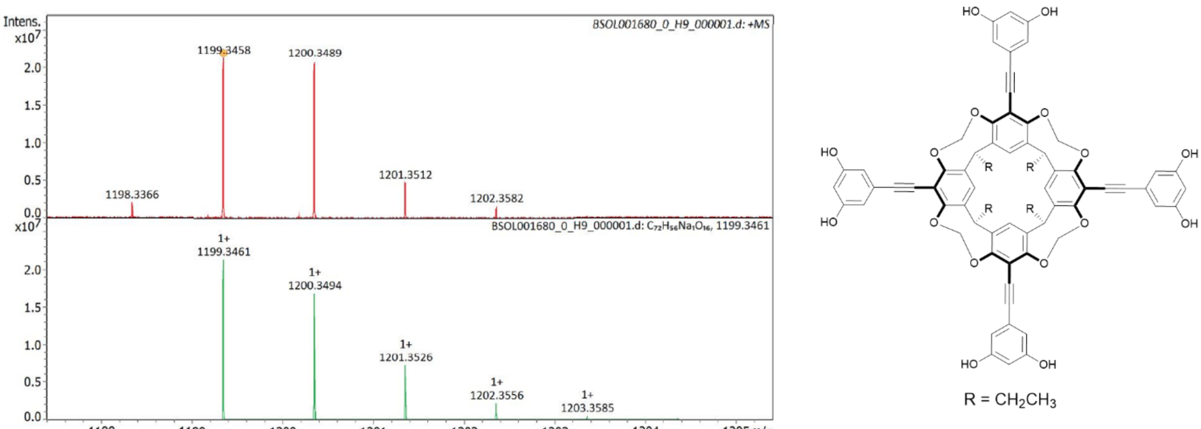

H

Figure S53: HR-ESIMS spectrum (+MS) of Compound **H** (Exact mass for $[M+Na]^{+}$: 1199.3461).

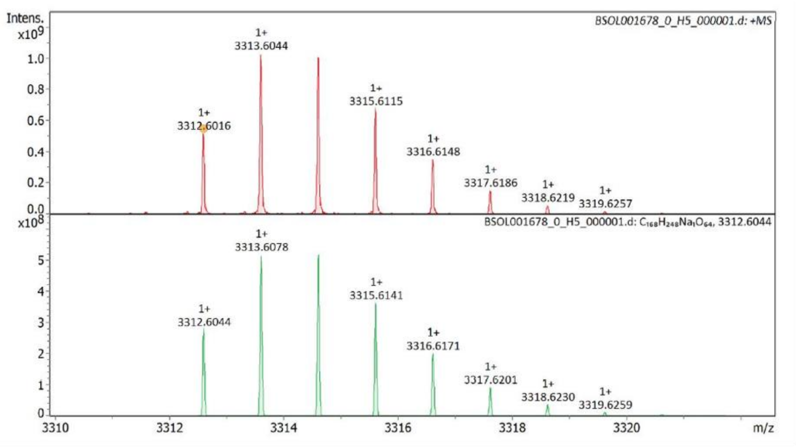

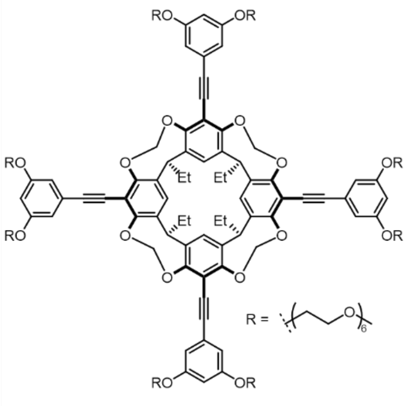

10

Figure S54: HR-ESIMS spectrum (+MS) of Cavitand **10** (Exact mass for $[M+Na]^+$: 3312.6044).

5. Crystallographic Data

Table SI1. Crystal data and structure refinement for **3** (KIC). Deposition Number: CCDC 2430285.

Deposition Number	CCDC 2430285	
Unit Cell Dimensions:	a = 12.2592 (2) Å<br>b = 15.2649 (2) Å<br>c = 15.3994 (2) Å	alpha = 117.222 (1)°<br>beta = 92.589 (1)°<br>gamma = 104.294 (1)°
Bond Precision:	C-C = 0.0026 Å	
Temperature:	100 K	
Volume:	2441.10 (7) Å^3	
Wavelenght:	0.71073	
Space Group:	P -1	
Hall Group:	-P 1	
Moiety Formula:	2 ($C_{40}H_{36}I_4O_8$), 7(C_2H_3N)	
Sum Formula:	$C_{94}H_{93}I_8N_7O_{16}$	
Mr	1295.97	
Density	1.763 g cm^{-3}	
Z	2	
Absorption Coefficient	2.607 mm^{-1}	
F00	1258.0	
Crystal Size	19 x 24 x 24	
Reflections Collected	15899	
N_{ref}	19330	
T_{min}, T_{max}	0.625, 1.000	
$T_{min'}$	0.549	
Correction Method	GAUSSIAN	
Data Completeness	0.905	
Theta (max)	34.902	
R indeces (all data)	R1 = 0.0276 wR2 = 0.0559	
S	1.010	
N_{par}	650	

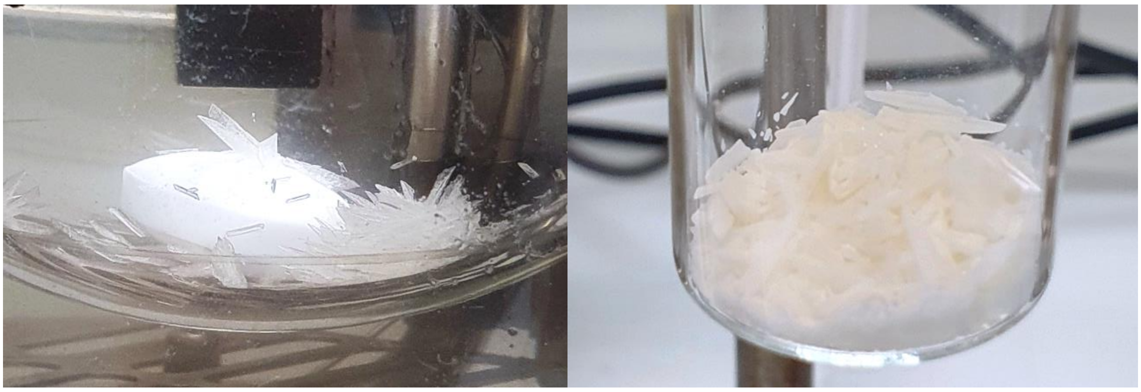

Figure S55. *KIC* (**3**) crystals obtained by slow cooling of an ACN/dioxane solution (left) and after washing/drying (right).

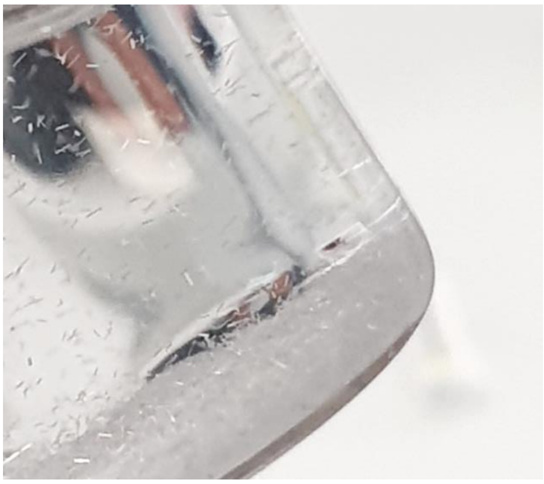

Figure S56. Tiny *KIC* (**3**) crystals obtained from the fast cooling of an ACN solution in a glass vial.

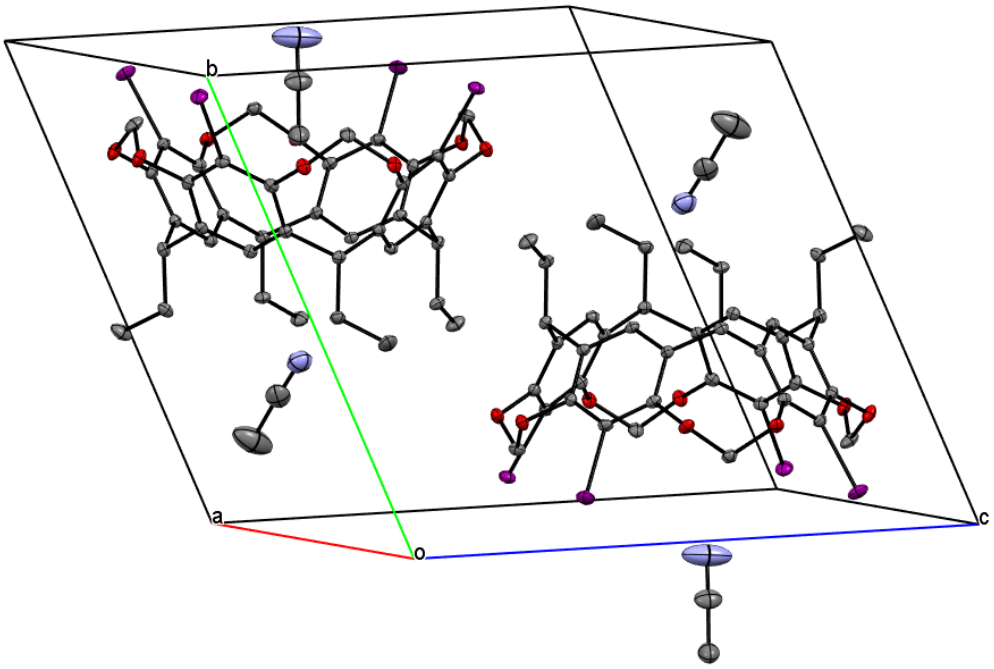

Figure S57. X-ray crystal structure unit cell of compound **3** (KIC) (the ellipsoid contour at 50% probability levels), showing a bound acetonitrile guest within its cavity. Deposition number CCDC 2430285.

Table S2. Crystal data and structure refinement for **D**. Deposition Number: CCDC 2430286.
D crystals were obtained by slow cooling of an ACN/EtOH solution.

Deposition Number	CCDC 2430286	
Unit Cell Dimensions:	a = 16.0210 (2) Å<br>b = 16.5975 (2) Å<br>c = 22.4809 (2) Å	alpha = 71.757 (1)°<br>beta = 75.733 (1)°<br>gamma = 82.576 (1)°
Bond Precision:	C-C = 0.0037 Å	
Temperature:	100 K	
Volume:	5493.49 (11) $Å^3$	
Wavelenght:	1.54184	
Space Group:	P -1	
Hall Group:	-P 1	
Moiety Formula:	$C_{104}H_{104}O_{32}$, C_2H_3N	
Sum Formula:	$C_{106}H_{107}NO_{32}$	
Mr	1906.93	
Density	1.153 g cm^{-3}	
Z	2	
Absorption Coefficient	0.710 mm^{-1}	
F00	2012.0	
Crystal Size	20 x 21 x 28	
Reflections Collected	18483	
N_{ref}	23315	
T_{min}, T_{max}	0.240, 1.000	
$T_{min'}$	0.780	
Correction Method	GAUSSIAN	
Data Completeness	0.949	
Theta (max)	83.066	
R indeces (all data)	R1 = 0.0766 wR2 = 0.2536	
S	1.087	
N_{par}	1298	

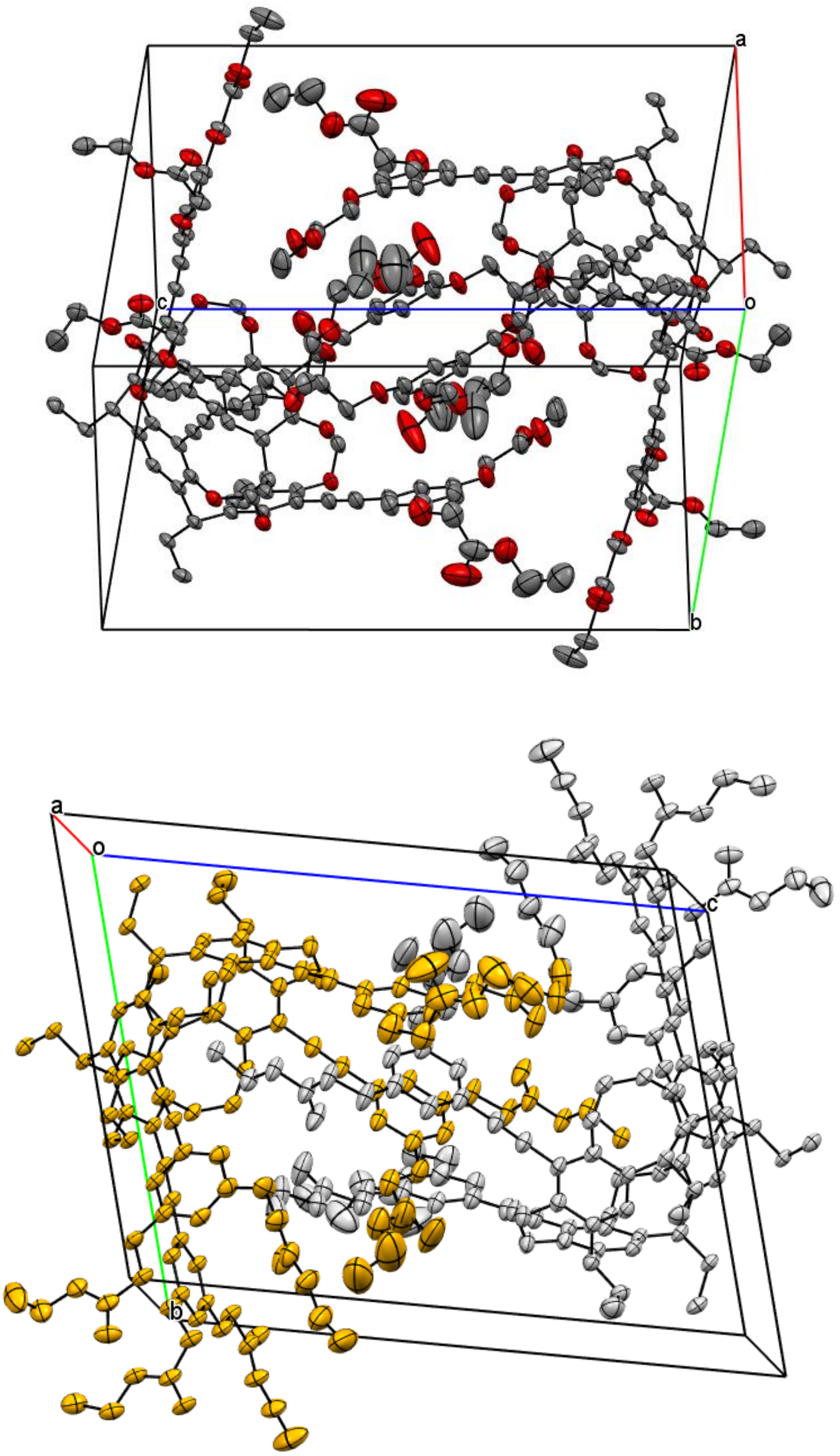

Figure S58. X-ray crystal structure unit cell of octa-ester cavitand **D** (the ellipsoid contour at 50% probability levels), showing the two hosts each with a bound side chain (ester moiety) within its cavity (Top). The two hosts have been marked in yellow and white (Bottom) to highlight the encapsulation of the ester groups.

Table S3. Crystal data and structure refinement for ethylene-bridged cavitand. Deposition Number: CCDC 2430293. Ethylene-bridged cavitand crystals were obtained by slow cooling of an acetone solution.

Deposition Number	CDCC 2430293	
Unit Cell Dimensions:	a = 13.6374 (1) Å<br>b = 16.7670 (2) Å<br>c = 18.8284 (2) Å	alpha = 94.980 (1)°<br>beta = 96.900 (1)°<br>gamma = 106.216 (1)°
Bond Precision:	C-C = 0.0025 Å	
Temperature:	100 K	
Volume:	4071.20 (11) $Å^3$	
Wavelenght:	1.54184	
Space Group:	P -1	
Hall Group:	-P 1	
Moiety Formula:	$C_{84}H_{80}O_{12}$, C_3H_6O	
Sum Formula:	$C_{87}H_{86}NO_{13}$	
Mr	1339.55	
Density	1.093 g cm^{-3}	
Z	2	
Absorption Coefficient	0.581 mm^{-1}	
F00	1424.0	
Crystal Size	17 x 21 x 23	
Reflections Collected	15496	
N_{ref}	17335	
T_{min}, T_{max}	0.448, 1.000	
$T_{min'}$	0.870	
Correction Method	GAUSSIAN	
Data Completeness	0.975	
Theta (max)	79.896	
R indeces (all data)	R1 = 0.0600 wR2 = 0.1852	
S	1.043	
N_{par}	1010	

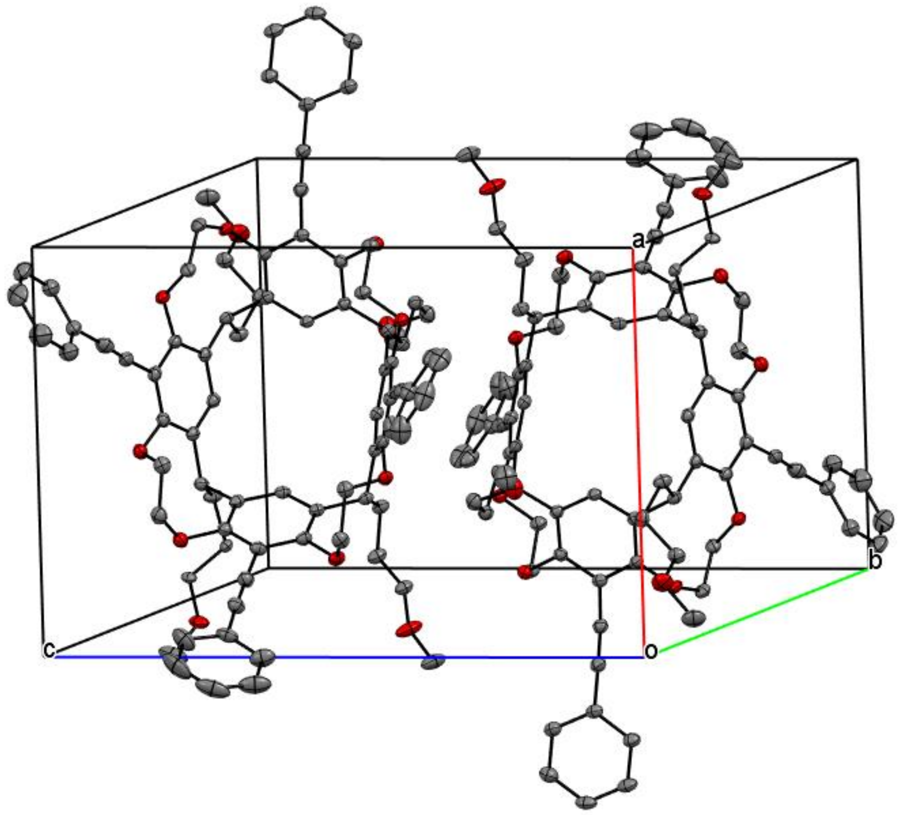

Figure S59. X-ray crystal structure unit cell of a model ethylene-bridged cavitand (the ellipsoid contour at 50% probability levels), functionalized through Sonogashira coupling between a derivative of **3** (KIC with methylether feet instead of ethyl one) with phenylacetylene (Ph-C≡CH), taken in account as model acetylene derivative.

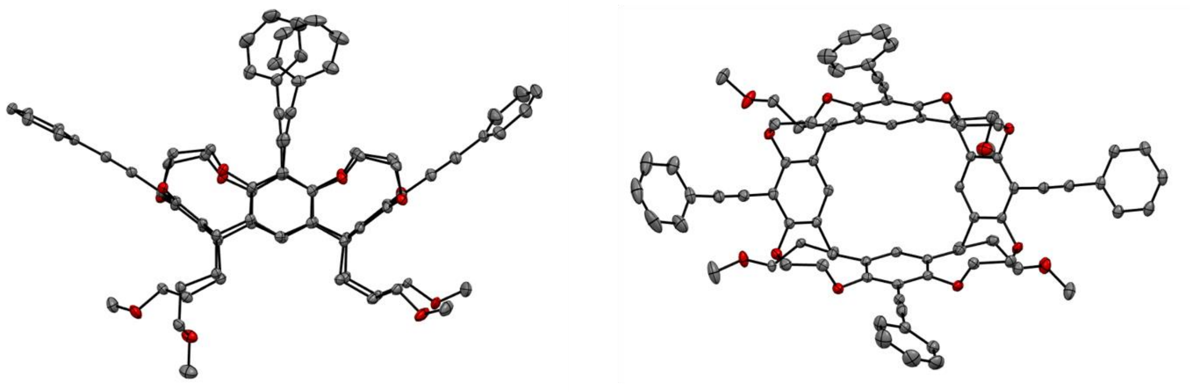

Figure S60. Side (left) and top (right) views of the X-ray crystal structure of model ethylene-bridged cavitand (the ellipsoid contour at 50% probability levels). Ethylene-bridged cavitand crystals were obtained by slow cooling of an acetone solution.

6. Binding Experiments

Preliminary NMR complexation tests revealed slow exchange compared to the chemical shift NMR timescale in the formation of complexes with a 1:1 stoichiometry (see Figure SI58), determined from direct integration of the peaks. The host concentration was kept constant at 1 mM in a volume of 500 µL of D_2O, and dimethyl sulfone ($DMSO_2$) was used as an internal standard for its titration. Each guest was added in concentrations ranging from 0.3 to 0.8 in steps of 0.1 mM, using a stock solution in DMSO-d_6, ACN-d_3 or acetone-d_6. In cases where the ^{1}H NMR spectrum appeared quite broad (e.g. ^{1}H NMR spectrum of **10** in D_2O), only two guest concentrations (0.4 and 0.8 equivalents) were explored for practical reasons. We want to clarify that the purpose of these binding experiments is to demonstrate the ability of host **7**-**10** to encapsulate organic molecules in aqueous solution. More detailed binding studies, along with their discussion and interpretation, are ongoing and will be reported in a separate paper.

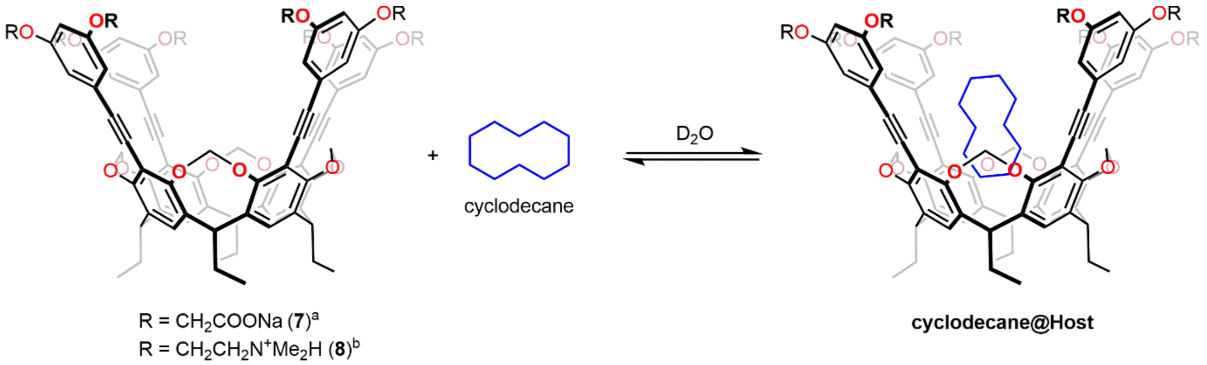

R = CH_2COONa (**7**)[a]
R = $CH_2CH_2N^+Me_2H$ (**8**)[b]
R = $CH_2CH_2N^+Me_3I^-$ (**9**)
R = $(CH_2CH_2O)_6H$ (**10**)

Figure SI61. Formation of the cyclodecane@Host complex in D_2O.
[a]Deprotonated **7** is formed *in situ*, adding 9 eq of NaOD.
[b]Protonated **8** is formed *in situ*, adding 9 eq of HCl.

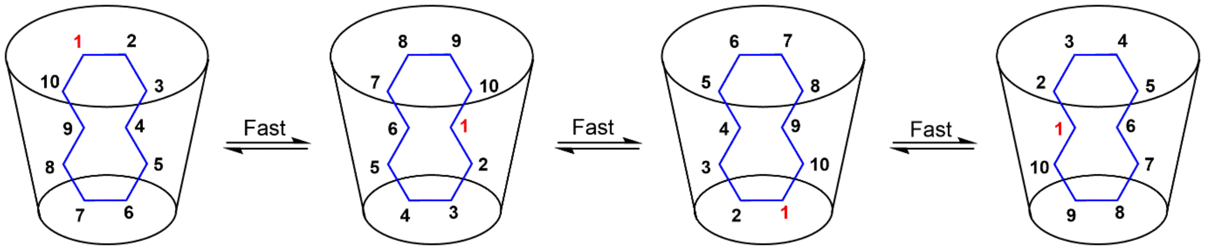

Figure S62. Kinetics of the bound cyclodecane in hosts **7**-**10**.
The rotation inside the cavity is fast with respect to the NMR time scale, leading to observed an averaged peak (singlet) of the bound gust. The guest's peak (singlet) is observed at higher magnetic field due to the anisotropic effect derived by the aromatic walls.

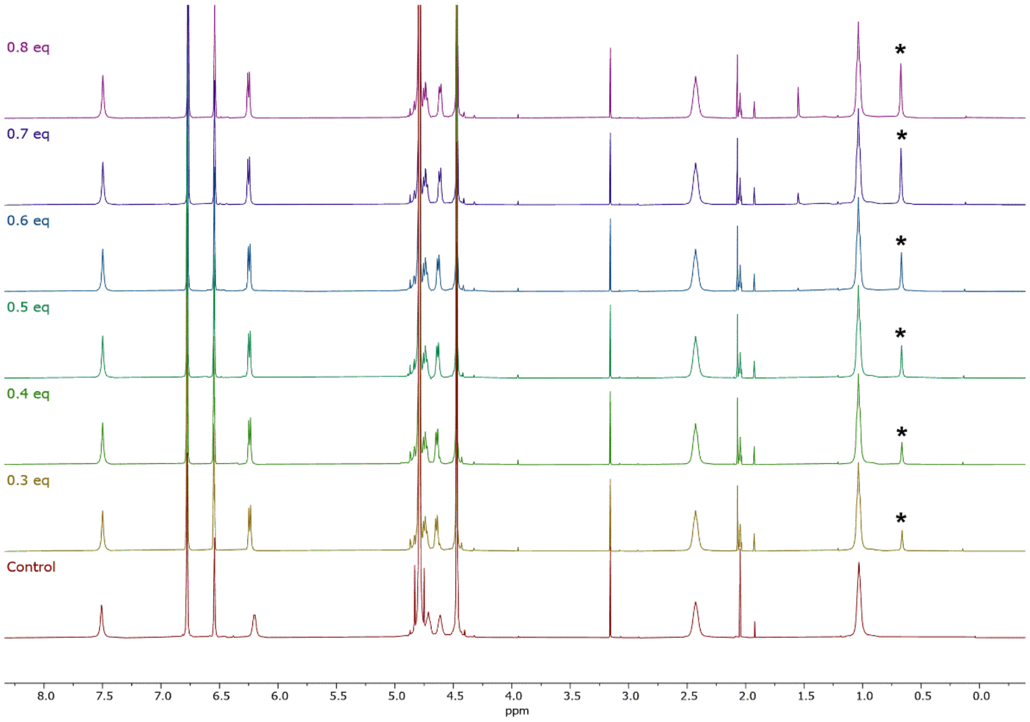

Figure S63. NMR titration of Host **7** with cyclodecane. Cyclodecane's peak (singlet) is marked with a black star. Binding constant (K_a) value for cyclodecane@**7** complex was calculated to be 3.1 x 10^2 M^{-1}.

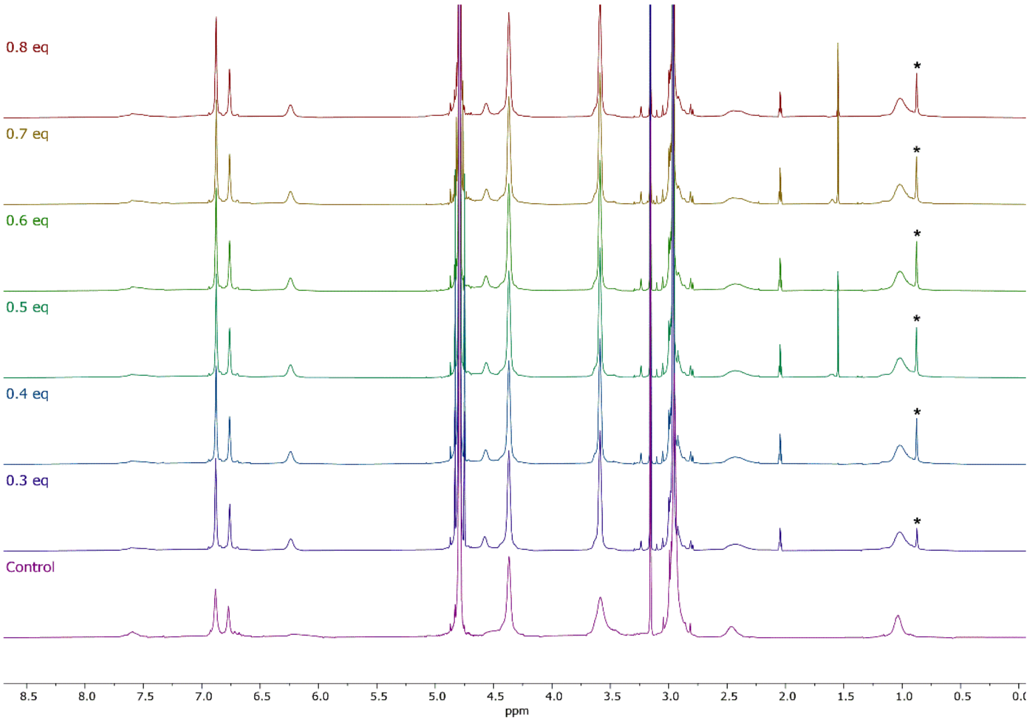

Figure S64. NMR titration of Host **8** with cyclodecane. Cyclodecane's peak (singlet) is marked with a black star. Binding constant (K_a) value for cyclodecane@**8** complex was calculated to be 3.0 x 10^2 M^{-1}.

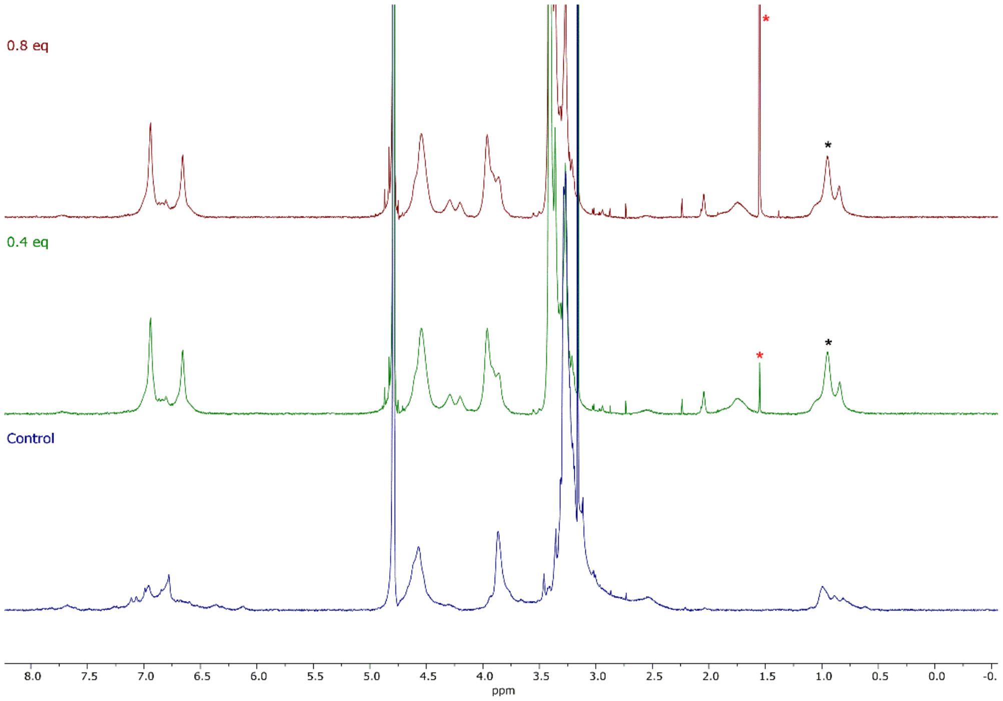

Figure S65. NMR titration of Host **9** with cyclodecane. Bound cyclodecane' and free-guest's peak(s) are marked with a black and red star, respectively.
Binding constant (K_a) value for cyclodecane@**9** complex was qualitatively calculated to ≈ 10^2 M^{-1}.

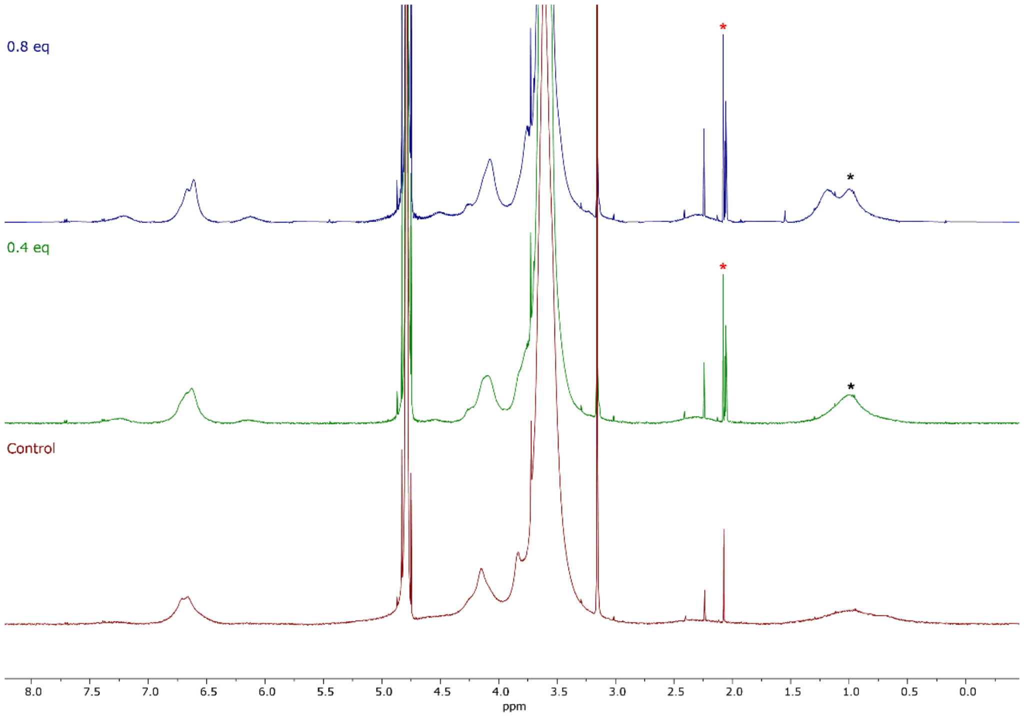

Figure S66. NMR titration of Host **10** with cyclodecane. Bound cyclodecane' and free-guest's peak(s) are marked with a black and red star, respectively.
Binding constant (K_a) value for cyclodecane@**10** complex was qualitatively calculated to ≈ 10^2 M^{-1}.

7. Computational Studies

Ab initio and density functional calculations were performed using the Gaussian09 program package.[6] Optimization of all the systems has been performed at HF/6-31G(d,p) level of theory in gas phase. Frequencies were calculated and checked out to make sure that all of them were not imaginary. Zero-point energy (ZPE) was included in each result.

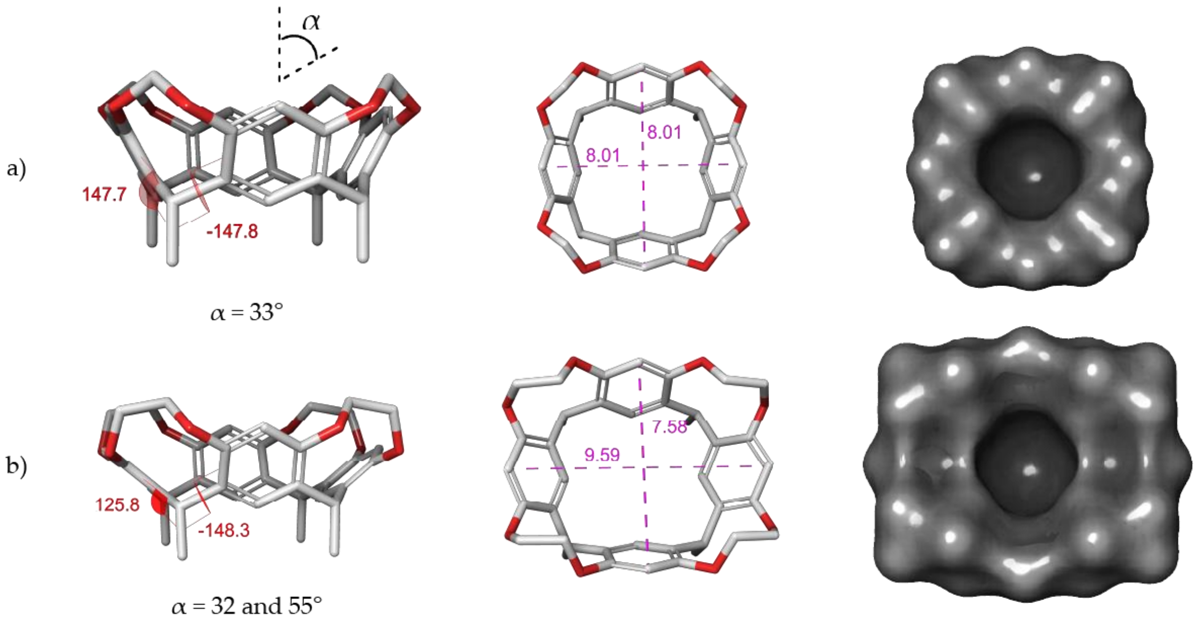

Figure S67. Side (left), top (middle) and surface (right) views of optimized structures of model methylene-bridged (a) and ethylene-bridged (b) cavitands. The angles α, indicative of the openness of the cavity, are calculated from the respective dihedral angles shown in red. The distances in Å between a selection of opposite aromatic carbons are shown in purple.

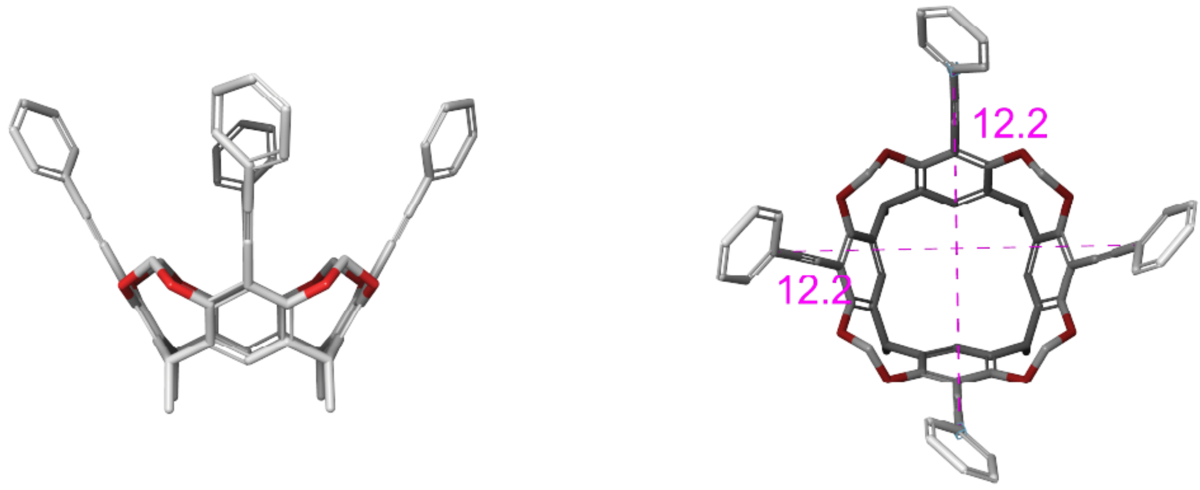

Figure S68. Side (left) and top (right) views of a model methylene-bridged deep-cavitnad optimized at HF level of theory. The length and width (Å) are shown in purple.

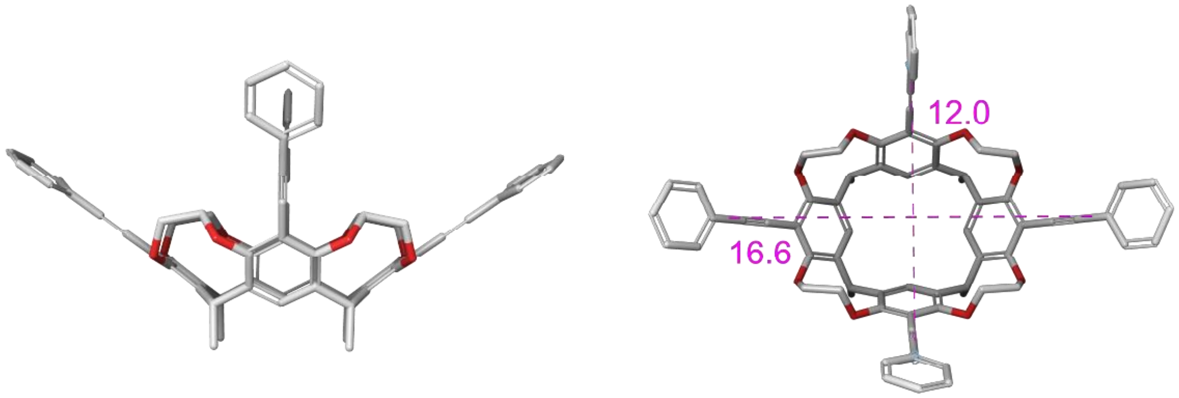

Figure S69. Side (left) and top (right) views of a model Ethylene-bridged deep-cavitnad optimized at HF level of theory. The length and width (Å) are shown in purple.

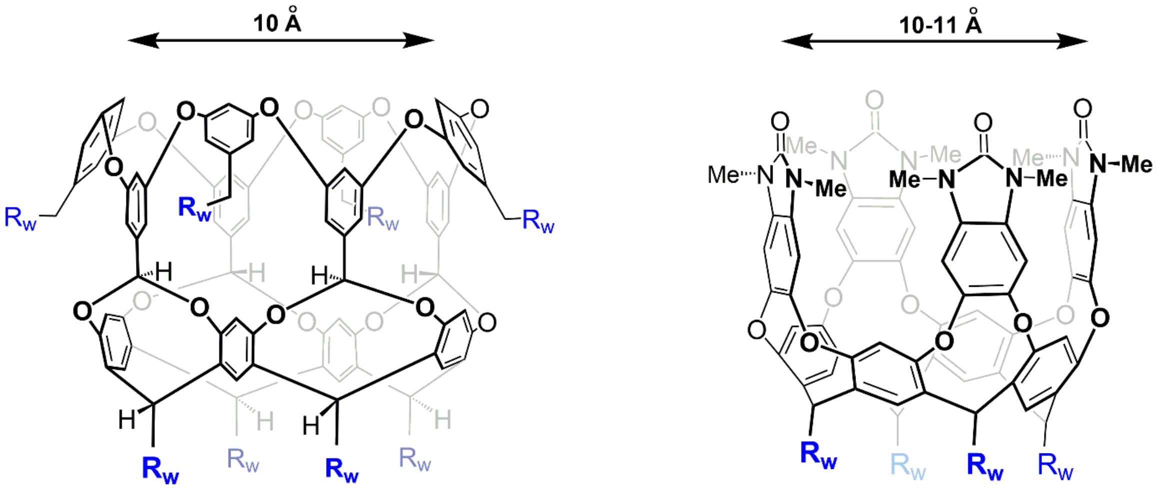

OA
(R_w = COOH)

OMC
(R_w = Imidazolium salt)

Figure S70. Size of the cavity (Å) in Gibb (OA - left) and Rebek cavitand (OMC - right).

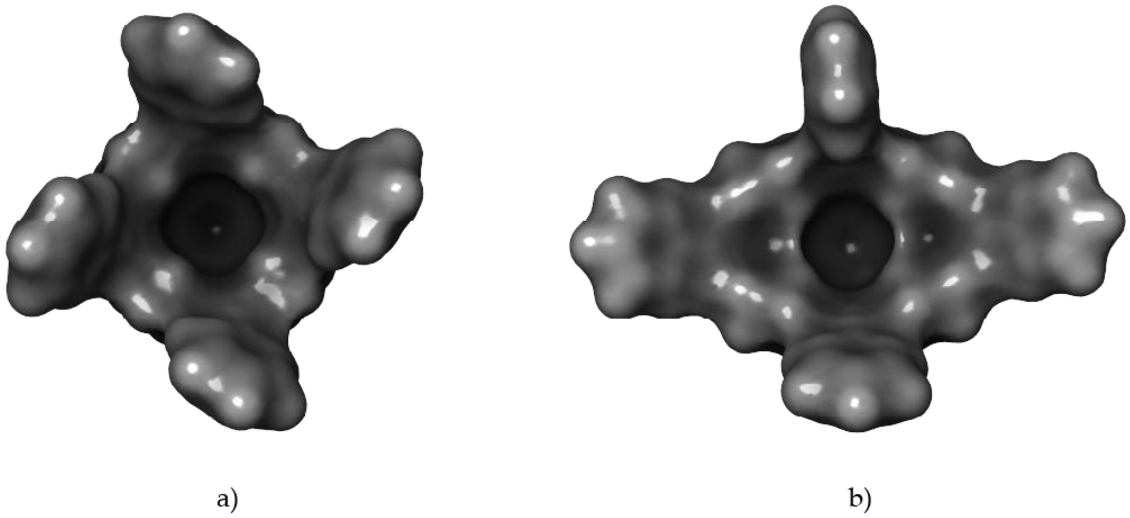

Figure S71. van der Waals (vdW) surfaces of the optimized structures of model methylene-bridged (a) and ethylene bridged deep-cavitand (b).

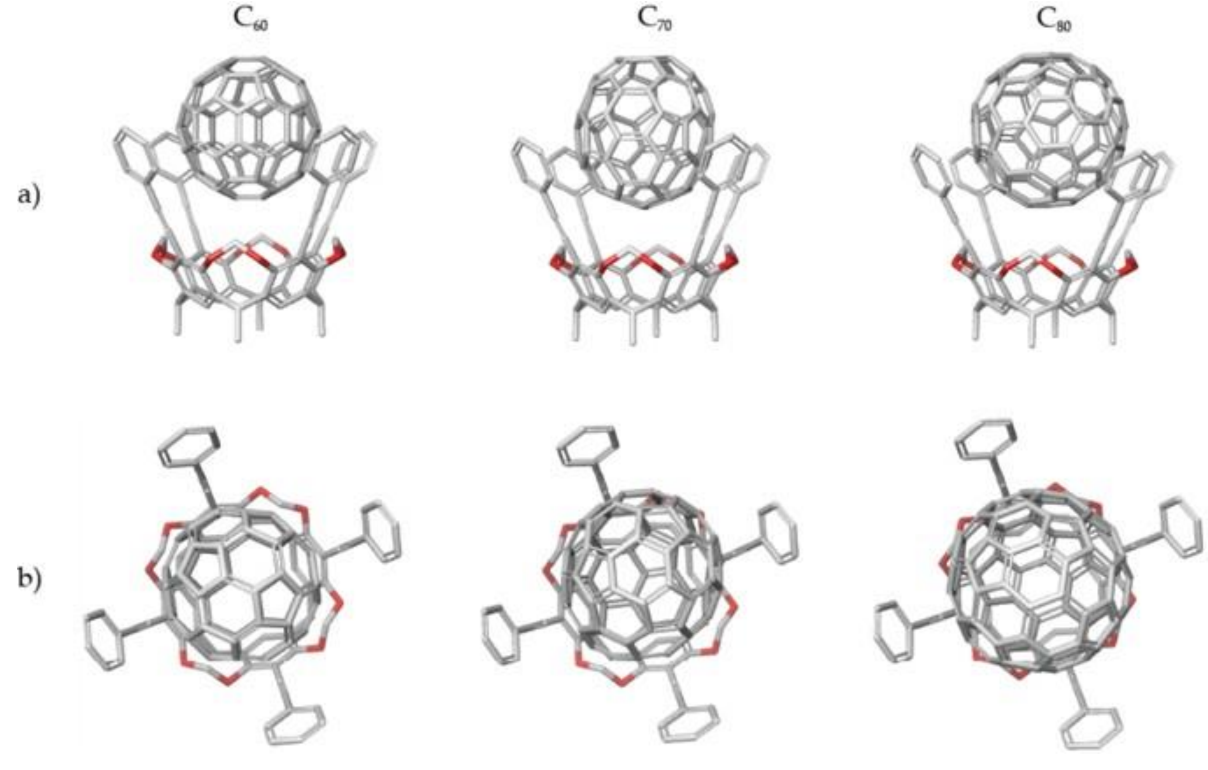

Figure S72. Side (a) and top (b) views of the optimized complexes between model methylene-bridged deep-cavitand and C_{60}, C_{70} and C_{80}.

Z-matrix (Cartesian coordinates) of the optimized model methylene bridged cavitand at PM6 level of theory:

O	14.72198000	13.50775000	16.36741300
O	14.28076600	12.41046800	18.37070300
O	10.38164500	11.79392200	21.11299300
O	8.15896200	12.45267400	20.93848700
O	5.60628800	15.00478200	17.77208200
O	6.05766000	16.13440800	15.78989000
O	9.98060900	16.84084000	13.10652500
O	12.19554500	16.15609200	13.25922500
C	13.34679800	14.73461300	14.82208400
C	13.85889800	14.57413800	16.12775200
C	13.60578000	15.50615400	17.15176200
C	12.82609300	16.63005800	16.85654700
H	12.61076100	17.35197900	17.64651000
C	12.31004400	16.84621200	15.57393200
C	12.59029100	15.90059500	14.57039500
C	11.41668800	18.02274000	15.23528600
H	11.56270300	18.25821700	14.13498100
C	11.74979800	19.29265500	16.01542600
H	11.09582400	20.11901400	15.70712800
H	11.63896300	19.17254300	17.09783100
C	10.87881700	15.75103000	12.86994100
H	10.55873100	14.82375100	13.35904000
H	10.95003500	15.73482600	11.76451000
C	8.04723800	16.36511000	14.45696800
C	9.35089600	16.89635500	14.34783800
C	9.98703000	17.55323800	15.41755600
C	9.28942000	17.69778700	16.62187700
H	9.77308500	18.19992200	17.46170600
C	7.98898100	17.20447100	16.77759900
C	7.38497000	16.54582100	15.69071700
C	7.22528300	17.30361900	18.08269800
H	6.11842800	17.31269000	17.83220100
C	7.51860800	18.58023400	18.86833600
H	6.91593800	18.61777200	19.78531900
H	8.56937700	18.67136500	19.16188400
C	5.80603200	14.84645900	16.36334000
H	4.78325100	14.62110300	16.00237900
H	6.58823400	14.11655600	16.12570800
C	6.95364700	13.65774900	19.24041000
C	6.71884600	14.89582700	18.60273100
C	7.50768500	16.03280600	18.85816600
C	8.55175200	15.92773300	19.78412900
H	9.17981700	16.79893100	19.98021600
C	8.81361200	14.73039600	20.45900900
C	8.00068600	13.61406600	20.18678900
C	9.96920100	14.56982300	21.42625100
H	9.67758700	13.77264600	22.17924200
C	10.29242400	15.83684300	22.21509100
H	10.59332700	16.67415500	21.57725400
H	9.42108800	16.16302600	22.79802800
C	9.12526000	11.49383500	20.49503400
H	8.81090800	10.57663800	21.03011100
H	9.19340300	11.43562100	19.40282200
C	12.26680800	12.07124600	19.64316100
C	11.24702500	12.64653400	20.43162900

C	11.13945700	14.03667200	20.62405000
C	12.08939200	14.86964400	20.02294100
H	12.01457600	15.95016900	20.15971800
C	13.12801400	14.34978500	19.24202300
C	13.20213500	12.95618000	19.06342700
C	14.14511300	15.22658400	18.53990600
H	15.09673600	14.61955500	18.42082600
C	14.51136900	16.48660100	19.32211900
H	13.65529800	17.14492500	19.50147400
H	14.93592300	16.22516600	20.30031800
C	14.17210700	12.31894900	16.94552100
H	14.93806300	11.55826800	16.69677000
H	13.14788900	12.11977900	16.61067700
C	13.57382700	13.78193200	13.80821600
C	13.75689700	12.97055100	12.92860200
C	13.95191800	12.02665700	11.89027900
C	13.62512800	12.36692600	10.56333100
H	13.23800600	13.36000700	10.33860500
C	13.80616900	11.43166800	9.54285500
C	14.31152300	10.15996400	9.83389700
H	14.44982000	9.43377900	9.03489400
C	14.64232400	9.82246300	11.15104500
C	14.46616600	10.74865600	12.18062100
H	14.72897500	10.49065800	13.20575500
C	7.43466400	15.69515500	13.37861800
C	6.90924200	15.12829000	12.44700500
C	6.29813600	14.46748900	11.35297100
C	4.96167700	14.03329400	11.44726100
H	4.39709400	14.21059600	12.36214900
C	4.36638500	13.38391300	10.36430300
C	5.09229100	13.16400800	9.18783300
H	4.62284700	12.65766800	8.34655600
C	6.41937500	13.59693000	9.09182100
C	7.02626100	14.24828500	10.16753500
H	8.05682700	14.59218700	10.09192600
C	6.17839000	12.51763400	18.94659600
C	5.51498400	11.53801700	18.69055500
C	4.74624100	10.38800000	18.38507300
C	4.97038100	9.18417000	19.07951100
H	5.72665300	9.14194200	19.86123300
C	4.21520500	8.05231400	18.76621500
C	3.23737900	8.11182000	17.76783600
H	2.65155100	7.22631300	17.52674300
C	3.00974100	9.30903200	17.08041500
C	3.75812900	10.44772400	17.38370200
H	3.57919000	11.38186700	16.85485500
C	12.35948800	10.67681900	19.45836800
C	12.46238900	9.47838500	19.32278300
C	12.59011900	8.07670400	19.16160700
C	11.48425600	7.23290000	19.37539200
H	10.52573100	7.65875700	19.66729100
C	11.62456400	5.85228800	19.21738100
C	12.85877000	5.30707300	18.84902100
H	12.96337900	4.22978200	18.72832900
C	13.96017900	6.14440900	18.63830200
C	13.83281700	7.52572400	18.79255700
H	14.68965100	8.18041200	18.63633600

H	11.11004400	15.65300600	22.92435500
H	15.26884600	17.07068100	18.78293600
H	12.78449300	19.60526300	15.82267800
H	7.26481100	19.46765200	18.27372100
H	2.24459800	9.35424400	16.30665500
H	4.38788800	7.12064400	19.30326000
H	10.76913600	5.19875900	19.38392700
H	14.92131900	5.71691300	18.35539800
H	15.03957800	8.83305400	11.37440300
H	13.55358300	11.69400700	8.51599700
H	6.98209700	13.42774800	8.17412000
H	3.33185800	13.04957000	10.43520100

Z-matrix (Cartesian coordinates) of the optimized model ethylene bridged cavitand at PM6 level of theory:

O	6.37575300	12.39584300	2.76705200
O	4.00817200	11.65050100	1.12511500
O	0.40858700	8.51544900	0.72253400
O	-0.82497100	6.08729300	1.93622000
O	-0.84287200	5.44666200	6.74605700
O	0.35824800	7.49734100	8.54388900
O	3.91472600	10.68023300	8.94644900
O	6.30757800	11.81060200	7.58406700
C	6.68393000	7.92844200	7.33113600
H	6.60451600	7.31991900	8.24064200
H	6.51632500	7.27017900	6.47332000
C	6.72740800	8.56341200	2.08182500
H	6.66083700	8.19004500	1.05239000
H	6.54141500	7.72108400	2.75500100
C	2.98921700	5.29654300	1.63317000
H	3.75871700	5.28379600	2.41114700
H	3.49174600	5.43222900	0.66740600
C	2.97596600	4.63033300	6.87615100
H	3.75203800	4.82164100	6.12941700
H	3.46872500	4.51861500	7.85004400
C	1.20204600	5.87539000	5.59484800
C	-0.20334800	5.78124100	5.55942700
C	-0.19377000	6.09636800	3.17369900
C	1.67922800	8.80916000	1.21216400
C	1.62504200	7.91832900	8.14853200
C	1.21236300	6.19093000	3.17470900
C	5.82771900	9.97318100	6.13859700
C	1.89026300	6.08664300	4.39486900
H	2.97756100	6.17684200	4.41089300
C	3.79494000	8.10748600	2.13136500
H	4.42818100	7.32628400	2.55646800
C	-4.96775300	5.70540100	4.32728400
C	-0.93691000	5.91688600	4.36049300
C	3.49281500	10.38986700	1.41698900
C	2.15185900	10.13074200	1.05552100
C	1.91093600	5.72872100	6.92630700
H	1.13898500	5.40488500	7.69469800
C	-3.55324500	5.77750500	4.33747100
C	2.44952400	7.06736500	7.38662900
C	2.07831800	9.16897200	8.62335800
C	4.32491300	9.39272700	1.96155100
C	1.32686300	11.14969500	0.53502200

C	3.42000800	9.52070500	8.35450700
C	4.27159900	8.69095800	7.59965500
C	5.85544000	10.26921800	3.71587500
C	1.23308200	10.02681800	9.35763700
C	6.08321700	11.35029500	6.29265800
C	-1.52936800	6.52039900	7.43897600
H	-1.71175200	6.03137300	8.41914900
H	-2.48135100	6.71416100	6.91151300
C	-0.70207500	7.79346700	7.58656100
H	-0.28693700	8.14647100	6.62834300
H	-1.28627500	8.60488700	8.06158400
C	6.20740200	12.22116200	5.18793400
C	2.48634400	7.78755100	1.74977400
C	-2.34438000	5.84451900	4.34687200
C	-5.67517000	5.90288500	3.12610500
H	-5.13205500	6.09418500	2.20254000
C	1.93251900	6.38199200	1.85511500
H	1.16510700	6.27012600	1.02483400
C	6.44550000	13.59936900	5.36033700
C	0.51227700	10.76891100	9.98646700
C	6.11475800	11.64141600	3.90387100
C	0.62673900	12.02596500	0.07965800
C	-5.66800700	5.43845500	5.51911300
H	-5.11956200	5.27092600	6.44447400
C	-1.49483400	7.31283300	1.54322300
H	-2.44472300	7.37855800	2.10478000
H	-1.68226500	7.09515100	0.47064300
C	5.22194200	12.54630700	8.20389700
H	5.20095500	13.56134400	7.76570200
H	5.58237800	12.57593400	9.25393700
C	3.94620200	12.59621900	2.23415200
H	3.66522000	12.09142800	3.17286000
H	3.15401800	13.31352100	1.94481500
C	3.86417000	11.86012100	8.08910900
H	3.05645800	12.48256400	8.51971500
H	3.61090000	11.58706800	7.05158800
C	5.31219500	13.27128300	2.31289100
H	5.69852600	13.53937100	1.30713900
H	5.28973600	14.15597800	2.97530600
C	-7.76498500	5.58455200	4.31226700
H	-8.85258800	5.54011800	4.30673600
C	-0.64827600	8.56853700	1.72737700
H	-1.21990000	9.48369400	1.48099500
H	-0.22796900	8.65454900	2.74301000
C	-7.06291600	5.38060800	5.50492100
H	-7.60573800	5.17380800	6.42664200
C	6.89075000	16.16685800	5.69440300
C	6.65143700	14.78275700	5.51282700
C	-7.07015800	5.84245500	3.12569300
H	-7.61818000	5.99506000	2.19733200
C	-0.32124400	11.64774100	10.72051900
C	-1.68538600	11.34862400	10.89872500
H	-2.09856500	10.43310400	10.47923600
C	-0.19529900	13.04842300	-0.45397400
C	0.30807400	14.35627300	-0.59258700
H	1.33234900	14.57541700	-0.29681900
C	-0.50723700	15.36105300	-1.11693000
H	-0.11781500	16.37253000	-1.22589600

C	-1.81977900	15.07296300	-1.50538800
H	-2.45081700	15.85981500	-1.91416400
C	-2.31994200	13.77303100	-1.37219300
H	-3.34009300	13.54998400	-1.68055900
C	-1.51523900	12.75912600	-0.84914000
H	-1.89892400	11.74495100	-0.75243700
C	0.21690800	12.82510700	11.27518600
H	1.27466100	13.04879100	11.14559300
C	7.07722800	16.68273100	6.99138900
H	7.04931700	16.01379500	7.85015500
C	-2.49859900	12.22439600	11.62120100
H	-3.55378800	11.99307000	11.76137400
C	-1.96317200	13.39546200	12.16812900
H	-2.60086900	14.07489800	12.73134200
C	6.94366600	17.02808500	4.58217100
H	6.81303000	16.62668300	3.57820700
C	7.30764200	18.04828300	7.16740200
H	7.45379600	18.44780400	8.17083600
C	7.35468800	18.90382800	6.06155800
H	7.53465400	19.96792300	6.20416200
C	-0.60684200	13.69235600	11.99522900
H	-0.19046500	14.60207200	12.42599300
C	7.17463600	18.39183000	4.77210100
H	7.21767000	19.05852800	3.91162300
C	5.71009200	9.10876900	7.37800700
H	6.00625900	9.74585500	8.27126800
C	3.75889300	7.48175700	7.11411700
H	4.40643100	6.83303400	6.52048000
C	5.70956700	9.45398000	4.84425000
H	5.49397000	8.39266900	4.71215700
C	5.76122700	9.72971100	2.30276700
H	6.07912400	10.56021000	1.59524700
H	2.52162100	4.30298200	1.62262000
H	2.51782500	3.66358900	6.62787900
H	7.71946800	8.28826100	7.26699200
H	7.76368900	8.88774700	2.24662400

Z-matrix (Cartesian coordinates) of the optimized HG complex between model methylene bridged cavitand and model fullerene derivative (C_{60}) at PM6 level of theory:

O	14.74920100	13.38605300	16.44998800
O	14.31642100	12.34823400	18.48775900
O	10.44084000	11.84472000	21.28926500
O	8.22220500	12.53997700	21.17217100
O	5.61357800	15.07926200	18.03200200
O	6.04690500	16.11710700	15.99456000
O	9.91935300	16.60897300	13.18734200
O	12.13774100	15.91438000	13.30300100
C	13.36994900	14.57525500	14.87695400
C	13.88912900	14.44952400	16.18423800
C	13.64304100	15.40806200	17.18470800
C	12.85650300	16.52065300	16.86634700
H	12.65310400	17.26800800	17.63575300
C	12.31795400	16.69289000	15.58643800
C	12.58202600	15.71607700	14.60864900
C	11.42658000	17.86290000	15.22303200
H	11.54404200	18.05014800	14.10985600

C	11.79703000	19.16090200	15.93803300
H	11.14404200	19.98171000	15.61336600
H	11.71677900	19.08891400	17.02742800
C	10.80333900	15.50000800	12.98791800
H	10.49437800	14.60632800	13.54393100
H	10.83528700	15.41727200	11.88344600
C	8.00186600	16.25484900	14.59782200
C	9.31929200	16.73811900	14.43830100
C	9.99730900	17.42195500	15.46438100
C	9.33744000	17.62719400	16.68112400
H	9.85576600	18.14938800	17.48768700
C	8.03143600	17.17054400	16.89037100
C	7.38059800	16.49132900	15.84380500
C	7.30784000	17.33460400	18.21141200
H	6.19498600	17.36555000	17.99093000
C	7.65628700	18.62823900	18.94512200
H	7.08053900	18.71228100	19.87632000
H	8.71673500	18.70091500	19.20705700
C	5.77939900	14.86009200	16.62625500
H	4.74420000	14.64361000	16.29604000
H	6.54058100	14.10257300	16.40209000
C	6.95344500	13.74855600	19.52349700
C	6.74359100	14.96622200	18.83929100
C	7.57885300	16.08371300	19.02231800
C	8.65141200	15.97773100	19.91479800
H	9.31084100	16.83557200	20.06065300
C	8.90289200	14.79461300	20.61821100
C	8.04967900	13.69466600	20.41189300
C	10.08128200	14.63451600	21.55693600
H	9.79684400	13.85825800	22.33449100
C	10.44271300	15.91306400	22.31057900
H	10.73559700	16.73374200	21.64791800
H	9.59222400	16.26206000	22.91102500
C	9.16476700	11.57002600	20.70037600
H	8.84876000	10.65284100	21.23581100
H	9.20588500	11.51660600	19.60544500
C	12.32083900	12.06331500	19.80270800
C	11.31223700	12.67076100	20.58227100
C	11.22392000	14.06638700	20.74015800
C	12.17085100	14.87342700	20.10010600
H	12.10841400	15.95794400	20.20866500
C	13.19103500	14.32212500	19.31693300
C	13.25236000	12.92311200	19.17993900
C	14.20206400	15.17028800	18.57271400
H	15.14889500	14.55486200	18.45991100
C	14.58558700	16.45335300	19.30741600
H	13.73660300	17.12505900	19.47004300
H	15.01577800	16.22247100	20.29085400
C	14.19025700	12.22041900	17.06687400
H	14.94320300	11.44268300	16.83038800
H	13.15880200	12.02537300	16.74863200
C	13.65016000	13.62209300	13.87714200
C	13.89321600	12.80144000	13.02129700
C	14.17847500	11.83880400	12.02216900
C	13.67284800	12.00456700	10.71840400
H	13.07376900	12.88099500	10.47714700
C	13.95009400	11.04720000	9.74089900

C	14.72885100	9.92720300	10.05181700
H	14.94270900	9.18388400	9.28589700
C	15.23686800	9.76426600	11.34523400
C	14.96693800	10.71413800	12.33207900
H	15.36724900	10.59358100	13.33738700
C	7.33040400	15.58604200	13.55434600
C	6.74774300	15.02588100	12.65310800
C	6.06906800	14.36305900	11.60097100
C	4.75522000	13.89495800	11.79436700
H	4.25693500	14.05785900	12.74876200
C	4.09644000	13.23152400	10.75748200
C	4.73599400	13.03249400	9.52903800
H	4.21785700	12.51479900	8.72362500
C	6.03930100	13.50237200	9.33348000
C	6.70930100	14.16717900	10.36225200
H	7.72087000	14.54034000	10.21012700
C	6.09137100	12.64767700	19.34573700
C	5.33576800	11.71238700	19.20572900
C	4.45424100	10.61695900	19.03312500
C	4.68763300	9.40815800	19.71613400
H	5.53724300	9.32293000	20.39136900
C	3.82271200	8.32814700	19.52861400
C	2.72589700	8.44423400	18.66790900
H	2.05475700	7.59890300	18.52459800
C	2.48832100	9.64739300	17.99459000
C	3.34556900	10.73488600	18.17309200
H	3.15845700	11.67488500	17.65655100
C	12.41125900	10.66280500	19.67081700
C	12.50541500	9.45997200	19.57218500
C	12.61881800	8.05313600	19.44901500
C	11.58193900	7.21735700	19.90580300
H	10.69739300	7.65364300	20.36699000
C	11.69755600	5.83256700	19.77032500
C	12.83963400	5.27423100	19.18578800
H	12.92530200	4.19387800	19.08286500
C	13.87375600	6.10328200	18.73736800
C	13.77009600	7.48954700	18.86584500
H	14.57671400	8.13695200	18.52439200
H	11.27774700	15.73290000	23.00038100
H	15.34350600	17.01146300	18.74194900
H	12.82930800	19.45165200	15.70236800
H	7.40896300	19.50202700	18.32795400
H	1.62978800	9.73784900	17.33051900
H	4.00181500	7.39273600	20.05715800
H	10.89587100	5.18499500	20.12368700
H	14.76511700	5.66613800	18.28854800
H	15.84810000	8.89476700	11.58334500
H	13.56090900	11.17528000	8.73160200
H	6.53323900	13.35163600	8.37424400
H	3.07956000	12.86999300	10.90571200
C	7.71235200	7.57521300	17.34480100
C	9.16880700	7.73010400	17.45757300
C	9.73448900	8.99494000	17.43686200
C	8.87902500	10.18138300	17.30427900
C	7.50555000	10.03395100	17.19575700
C	6.90463300	8.69364800	17.21808200
C	7.44406800	6.37979500	16.53412000

C	8.73494000	5.79608700	16.14591400
C	9.80103700	6.63017400	16.71710200
C	10.96340800	6.85743600	15.99778700
C	10.96638800	9.23580700	16.67416900
C	9.58182700	11.15513900	16.45826200
C	8.87100900	11.92770900	15.55382800
C	7.41432500	11.77375500	15.44111700
C	6.75192800	10.85320800	16.23709700
C	5.68516400	10.01935500	15.66703100
C	5.77999400	8.68439700	16.27279600
C	5.52641200	7.55699900	15.50838800
C	6.38334700	6.37141500	15.64258200
C	8.89183600	5.23649300	14.88798800
C	7.76740300	5.22734100	13.94283300
C	6.54972900	5.77812300	14.30895400
C	5.79621700	6.59720500	13.35044400
C	5.16408900	7.69692100	14.09163500
C	5.07447800	8.95589800	13.51994600
C	5.34305800	10.15113400	14.33097600
C	6.04623500	11.12514400	13.48553900
C	7.05204200	11.91130800	14.02458500
C	10.87237900	10.57103700	16.06979100
C	9.73107200	11.00897600	11.61542600
C	10.79131800	11.01757600	12.50716800
C	11.64746600	9.83147300	12.64158300
C	11.39594400	8.70454600	11.87579100
C	10.27108900	8.69548500	10.93094900
C	8.00660400	9.65960700	10.69129900
C	7.37442100	10.75946100	11.43246700
C	8.44048900	11.59307200	12.00385800
C	8.28423700	12.15194100	13.26222700
C	9.40833500	12.16327200	14.20735000
C	10.62540200	11.61103700	13.84071000
C	12.01104200	9.69240900	14.05809600
C	12.10050200	8.43333000	14.62921000
C	11.83273800	7.23789600	13.81811600
C	11.49040900	7.36988900	12.48194300
C	10.42426500	6.53560300	11.91096900
C	9.67078500	7.35474800	10.95240200
C	8.29684800	7.20923900	10.84608700
C	7.44039300	8.39505600	10.71152200
C	6.21205400	10.53224500	12.15175800
C	5.61188200	9.19140600	12.17328000
C	6.20810500	8.15411200	11.47416200
C	6.30302900	6.81938600	12.08007300
C	7.59389700	6.23525700	11.69181500
C	8.30462600	5.46260400	12.59596900
C	9.76123000	5.61710900	12.70886600
C	10.12412400	5.47739200	14.12537500
C	11.12969000	6.26416000	14.66388800
C	11.37927400	10.79234200	14.79910300
C	11.56321800	8.19815500	15.97597800
C	9.46313200	9.81397300	10.80395600

8. References

1. J. N. Smith, N. T. Lucas, Rigid tetraarylene-bridged cavitands from reduced-symmetry resorcin[4]arene derivatives, *Chem. Comm.*, **2018**, 54, 4716-4719.
2. A fast and smooth synthetic methodology for its synthesis is reported in Scheme SI7. The reported synthesis has been followed to get the target molecule during the pandemic due to the long shipment time.
3. C. B. Aekeröy, N. Schultheiss, J. Desper, *C*-Pentyltetra(3-pyridyl)cavitand: A Versatile Building Block for the Directed Assembly of Hydrogen-Bonded Heterodimeric Capsules, *Org. Lett.*, **2006**, 8 (12), 2607-2610.
4. F. Gruppi, F. Boccini, L. Elviri, E. Dalcanale, Self-assembly of a cavitand-based heteronuclear coordination cage, *Tetrahedron*, **2009**, 65 (35), 7289-7295.
5. L. Sebo, F. Diederich, V. Gramlich, Tetrakis(phenylamidinium)-Substituted Resorcin[4]arene Receptors for the Complexation of Dicarboxylates and Phosphates in Protic Solvents. *Helvetica Chimica Acta*, **2000**, 83 (1), 93-113.
6. M. J. Frisch, G. W. Trucks, H. B. Schlegel, G. E. Scuseria, M. A. Robb, J. R. Cheeseman, G. Scalamani, V. Barone, B. Mennucci, G. A. Peterson, H. Nakatsuji, M. Caricato, X. Li, H. P. Hratchian, A. F. Izmaylov, J. Bloino, G. Zheng, J. L. Sonnenberg, M. Hada, M. Ehara, K. Toyota, TR. Fukuda, J. Hasegawa, M/ Ishida, T. Nakajima, Y. Honda, O. Kitao, H. Nakai, T. Vreven, K. Throssell, J. A. Montgomery, Jr., J. E. Peralta, F. Ogliaro, M. Bearpark, J. J. Heyd, E. Brothers, K. N. Kudin, V. N. Staroverov, R. Kobayashi, J. Normand, K. Raghacachari, A. Rendell, J. C. Burant, S. S. Iyengar, J. Tomasi, M. Cossi, N/ Rega, J. M. Millam, M. Klene, J. E. Knox, J. B. Cross, V. Bakken, C. adamo, J. Jaramillo, R. E. Stratmann, R. Gomperts, O. Yazyev, A. J. Austin, R. Cammi, C. Pomelli, J. W. Ochterski, R. L. Martin, K. Morokuma, V. G. Zakrzewski, G. A. Voth, P. Salvador, J. J. Dannenberg, S. Dapprich, A. D. Daniels, Ö. Farkas, J. B. Foresman, J. V. Ortiz, J. Cioslowski and D. J. Fox, Gaussian 09, Revision C.01; Gaussian, Inc.: Wallingford, CT, **2009**.
